# Complete chloroplast of four *Sanicula* taxa (Apiaceae) endemic to China: lights into genome structure, comparative analysis, and phylogenetic relationships

**DOI:** 10.1186/s12870-023-04447-w

**Published:** 2023-09-21

**Authors:** Huimin Li, Mingsong Wu, Qiang Lai, Wei Zhou, Chunfeng Song

**Affiliations:** 1 Jiangsu Key Laboratory for the Research and Utilization of Plant Resources, Institute of Botany, Jiangsu Province and Chinese Academy of Sciences (Nanjing Botanical Garden Mem. Sun Yat-Sen), Nanjing, 210014 Jiangsu China; 2 Hainan Provincial Key Laboratory of Resources Conservation and Development of Southern Medicine, Hainan Branch of the Institute of Medicinal Plant Development, Chinese Academy of Medical Sciences and Peking Union Medical College, Haikou, 570311 China; 3 Key Laboratory for Bio-Resources and Eco-Environment, College of Life Science, Sichuan University, Chengdu, 610065 China

**Keywords:** Apiaceae, China, Chloroplast genome, Comparative analysis, Phylogeny, *Sanicula*

## Abstract

**Background:**

The genus *Sanicula* comprises ca. 45 taxa, widely distributed from East Asia to North America, which is a taxonomically difficult genus with high medicinal value in Apiaceae. The systematic classification of the genus has been controversial for a long time due to varied characters in key morphological traits. China is one of the most important distributed centers, with ca. 18 species and two varieties. At present, chloroplast genomes are generally considered to be conservative and play an important role in evolutionary relationship study. To investigate the plastome evolution and phylogenetic relationships of Chinese *Sanicula*, we comprehensively analyzed the structural characteristics of 13 Chinese *Sanicula* chloroplasts and reconstructed their phylogenetic relationships.

**Results:**

In present study, four newly complete chloroplast genome of *Sanicula* taxa by using Illumina sequencing were reported, with the typical quadripartite structure and 155,396–155,757 bp in size. They encoded 126 genes, including 86 protein-coding genes, 32 tRNA genes and 8 rRNA genes. Genome structure, distributions of SDRs and SSRs, gene content, among *Sanicula* taxa, were similar. The nineteen intergenic spacers regions, including *atp*H-*atp*I, *ndh*C-*trn*M, *pet*B-*pet*D, *pet*D-*rpo*A, *pet*N-*psb*M, *psa*J-*rpl*33, *rbc*L-*acc*D, *rpo*B-*trn*C, *rps*16-*trn*Q, *trn*E-*psb*D, *trn*F-*ndh*J, *trn*H-*psb*A, *trn*N-*ndh*F, *trn*S-*psb*Z, *trn*S-*trn*R, *trn*T-*trn*F, *trn*V-*rps*12, *ycf*3-*trn*S and *ycf*4-*cem*A, and one coding region (*ycf*1 gene) were the most variable. Results of maximum likelihood analysis based on 79 unique coding genes of 13 Chinese *Sanicula* samples and two *Eryngium* (Apiaceae-Saniculoideae) species as outgroup taxa revealed that they divided into four subclades belonged to two clades, and one subclade was consistent with previously traditional *Sanicula* section of its system. The current classification based on morphology at sect. *Sanicla* and Sect. *Tuberculatae* in Chinese *Sanicula* was not supported by analysis of cp genome phylogeny.

**Conclusions:**

The chloroplast genome structure of *Sanicula* was similar to other angiosperms and possessed the typical quadripartite structure with the conserved genome arrangement and gene features. However, their size varied owing to expansion/contraction of IR/SC boundaries. The variation of non-coding regions was larger than coding regions of the chloroplast genome. Phylogenetic analysis within these Chinese *Sanicula* were determined using the 79 unique coding genes. These results could provide important data for systematic, phylogenomic and evolutionary research in the genus for the future studies.

**Supplementary Information:**

The online version contains supplementary material available at 10.1186/s12870-023-04447-w.

## Introduction

*Sanicula* L. (Apiaceae-Saniculoideae), consists of ca. 45 taxa, is widely distributed from East Asia to North America [1, 2]. China is one of the most important distributed centers, with ca. 18 species and two varieties [3–5]. It was known as the considerably complex taxonomic genus, with its varied morphological characters in rhizomes, leaves, inflorescences and fruits, placed comparatively primitive within Subfam. Saniculoideae Burnett in primitive of Apiaceae [6–10]. Traditionally, based on the features of leaves, flower and fruits, Shan [6] divided the species of world *Sanicula* into five sections, i.e. *Tuberculatae*, *Pseudopetagnia*, *Sanicla*, *Sandwicenses* and *Sanicoria*, and demonstrated that the Chinese *Sanicula* taxa belonged to the former three sections. A classification was accepted by many later authors [3, 4, 11].

*Sanicula* had consistently been viewed as a relatively natural genus within the family Apiaceae [12–14]. Molecular phylogenetic analyses had also suggested that the genus was a monophyletic group yet based on few *Sanicula* samples by using the nuclear ribosomal internal transcribed spacer (ITS) region and chloroplast DNA (cpDNA) *rpl*16 intron, *rpo*C1 intron, *trn*Q-*rps*16 and *rps*16-*trn*K intergenic spacers[8, 13–17]. Then, a revised phylogeny of Apioideae and Saniculoideae in Apiaceae based on the 90 whole plastome sequences, including only four Chinese *Sanicula* species, suggested that sectional relationships in *Sanicula* were distinct from the traditional classification system [2].

Furthermore, based on recent wild observations in eastern, southern, and western China, the interspecific relationships of some groups in the genus were extremely perplexing [9, 10]. It was also mentioned by many authors, including Chen et al. [14, 17], Shan & Constance [6], and Yang et al. [2]. In addition, numerous species and varieties in *Sanicula* were poorly defined due to a lack of field studies and consistent characteristics for diagnostic methods in the literature [2, 6, 14, 17]. Therefore, further exploration into more stable genetic variations and effective markers is critical for utilizing and protecting the *Sanicula* plants.

Chloroplast (cp) genomes were highly conserved in terms of the genetic replication mechanisms in uniparental inheritance and possess the relatively high level of genetic variation resulting from the low selective pressure [18]. In addition to its low sequencing cost caused by rapid development of illumine and assembly technologies, the cp genome had been relatively more successful than fragments in resolving the relationship between many species at different taxonomic levels in many species [3, 19].

The plastomes of six Chinese *Sanicula* species were reported previously, including *S. astrantiifolia* H. Wolff ex Kretschmer, *S. chinensis* Bunge, *S. flavovirens* Z. H. Chen, D. D. Ma & W. Y. Xie, *S. giraldii* R. H. Shan & S. L. Liou, *S. lamelligera* Hance, *S. orthacantha* S. Moore and *S. rubriflora* F. Schmidt [2, 17, 20, 21]. However, it seemed that the samples of *S. chinensis* and *S. orthacantha* used in previous study were likely mixed up due to their same voucher information. Additionally, few *Sanicula* species had been involved in molecular studies [2, 22] and fewer effective markers were discovered to deal with their inter- and intra- specific relationships.

The aim of this study was to 1) determine the whole plastome sequence of 13 Chinense *Sanicula* taxa, including the four newly sequenced taxa; 2) compare the global structural patterns of available Chinese *Sanicula* cp genomes; 3) examine variations in the SSRs and repeat sequences among 13 *Sanicula* cp genomes; 4) to reconstruct the phylogeny of Chinese *Sanicula* taxa, and improve the understanding of the relationship and evolution in Chinese *Sanicula* taxa.

## Results

### Chloroplast genome structures of four taxa in Chinenese *Sanicula *L. and one species in *Eryngium* L

**Fig. 1:**
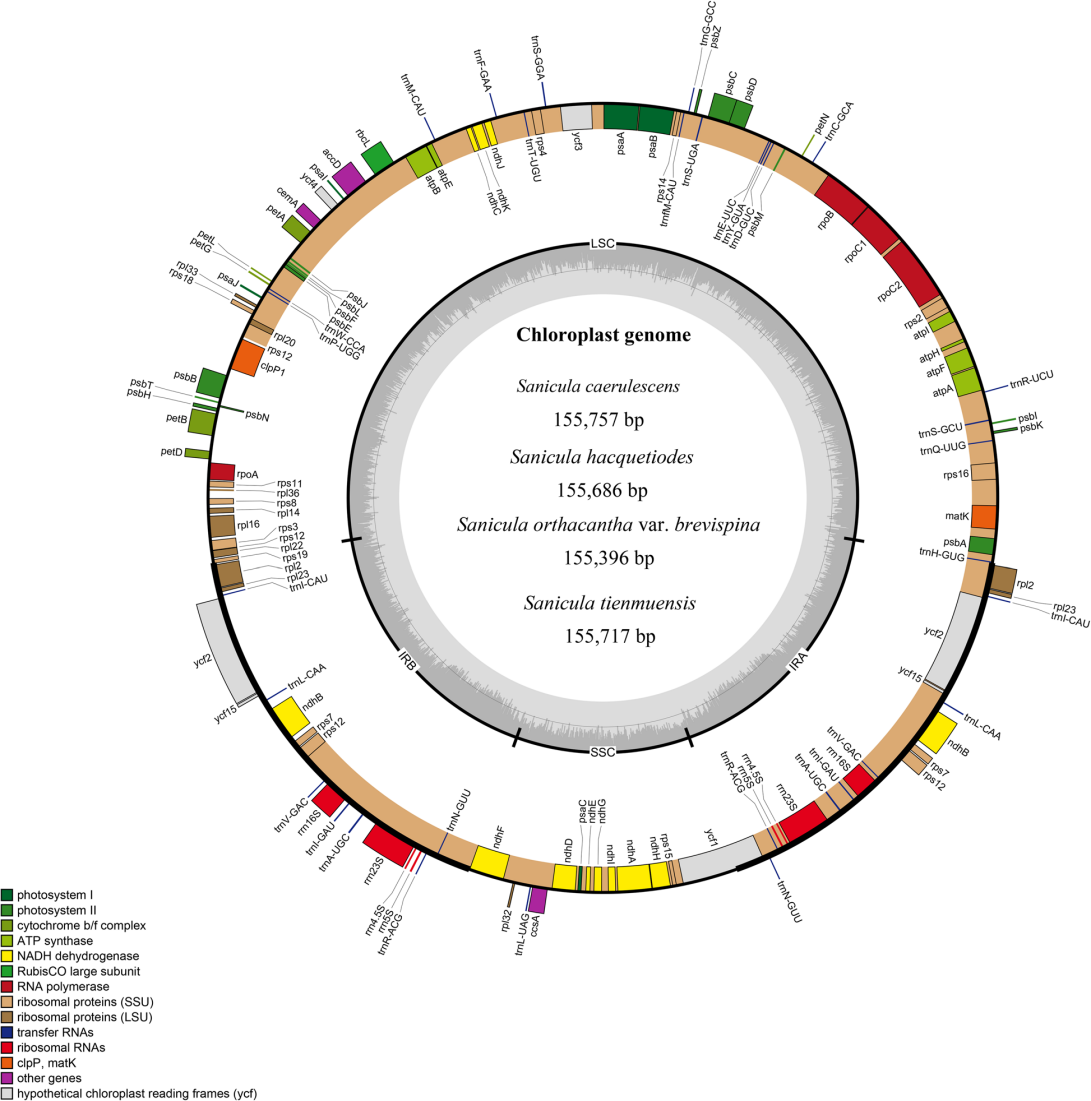
A circular gene map of four newly sequenced *Sanicula* chloroplast genomes. Genes shown outside are transcribed clockwise, and inside the circle are transcribed counterclockwise. Genes are color-coded to distinguish different functional groups. The dark grey and the light grey plots in the inner circle correspond to the GC content and AT content, respectively

All five new *Sanicula* cp genomes (Table 1) were similar to other species of *Sanicula* or other genera in Apiaceae [17]. The size of four new cp genome in *Sanicula* ranged from 155,396 bp in *S. orthacantha* var. *brevispina* to 155,757 bp in *S. caerulescens*, exhibiting a typical quadripartite structure with two single copy regions (LSC and SSC) which were separated by a pair of inverted repeats (IRa and IRb) (Fig. 1). The length of the large single-copy (LSC) ranged from 85,818 bp (*S. orthacantha* var. *brevispina*) to 86,209 bp (*S. caerulescens*), the small single-copy (SSC) ranged from 17,089 bp (*S. hacquetiodes*) to 17,106 bp (*S. tienmuensis*), and IR regions ranged from 26,225 bp (*S. caerulescens*) to 26,332 bp (*S. hacquetiodes*) (Table 1). The overall GC content ranged from 38.16% (*S. caerulescens*) to 38.21% (*S. hacquetiodes*).

**Table 1:** Summary of chloroplast genome features in this study, including four new chloroplast genomes of the *Sanicula* taxa and one newly in *Eryngium*, ^a^ showing the new chloroplast genome reported in this study

Species Name	GenBank Accession	Genome Size (bp)	LSC Length (bp)	SSC Length (bp)	IR Length (bp)	Overall GC content (%)	Coding regions size (%)	CDS regions size (%)	RNA regions size (%)	Noncoding regions size (%)	Number of unique genes	Number of unique genes			Number of total genes	Number of total genes			Reference
												**PCGs**	**tRNAs**	**rRNAs**		**PCGs**	**tRNAs**	**rRNAs**	
*Eryngium foetidum* (^a^)	OP703171	155,270	85,874	17,074	26,161	38.13	56.14%	50.31%	5.83%	43.86%	104	79	21	4	127	86	33	8	This Study
*E. planum*	MT561039	154,979	85,993	17,880	25,553	38.21	56.17%	50.34%	5.83%	43.83%	104	79	21	4	127	86	33	8	Wen et al. 2021 [22]
*Sanicula caerulescens* (^a^)	OP703178	155,757	86,209	17,098	26,225	38.16	55.92%	50.11%	5.81%	44.08%	103	79	20	4	126	86	32	8	This Study
*S. chinensis*	OP696651	155,378	85,660	17,074	26,322	38.25	56.06%	50.24%	5.82%	43.94%	103	79	20	4	126	86	32	8	This Study
*S. flavovirens*	OP703176	155,335	85,682	17,049	26,302	38.2	56.04%	50.22%	5.82%	43.96%	103	79	20	4	126	86	32	8	This Study
*S. flavovirens*	NC_061752	155,335	85,852	17,049	26,217	38.2	56.02%	50.19%	5.83%	43.98%	103	79	20	4	126	86	32	8	Yang et al*.* 2022 [2]
*S. giraldii*	OP703177	155,598	85,846	17,086	26,333	38.22	56.00%	50.19%	5.81%	44.00%	103	79	20	4	126	86	32	8	This Study
*S. hacquetiodes* (^a^)	OP703172	155,686	85,933	17,089	26,332	38.21	55.96%	50.15%	5.81%	44.04%	103	79	20	4	126	86	32	8	This Study
*S. lamelligera*	OP703174	155,764	86,174	17,106	26,242	38.17	55.94%	50.13%	5.81%	44.06%	103	79	20	4	126	86	32	8	This Study
*S. orthacantha*	OP703173	155,662	86,072	17,114	26,238	38.17	55.97%	50.16%	5.81%	44.03%	103	79	20	4	126	86	32	8	This Study
*S. orthacantha* var. *brevispina* (^a^)	OP703179	155,396	85,818	17,094	26,242	38.18	56.07%	50.25%	5.82%	43.93%	103	79	20	4	126	86	32	8	This Study
*S. orthacantha var. stolonifera*	MT561028	155,394	85,824	17,094	26,238	38.18	56.07%	50.25%	5.82%	43.93%	103	79	20	4	126	86	32	8	Wen et al. 2021 [22]
*S. rubriflora*	MT528260	155,721	85,981	17,060	26,340	38.18	55.92%	50.11%	5.81%	44.08%	103	79	20	4	126	86	32	8	Yang et al*.* 2022 [2]
*S. rubriflora*	NC_060324	155,700	85,981	17,053	26,333	38.18	56.00%	50.18%	5.82%	44.00%	103	79	20	4	126	86	32	8	Wang et al*.* 2021 [22]
*S. tienmuensis* (^a^)	OP703175	155,717	86,075	17,106	26,268	38.17	56.00%	50.19%	5.81%	44.00%	103	79	20	4	126	86	32	8	This Study

**Fig. 2:**
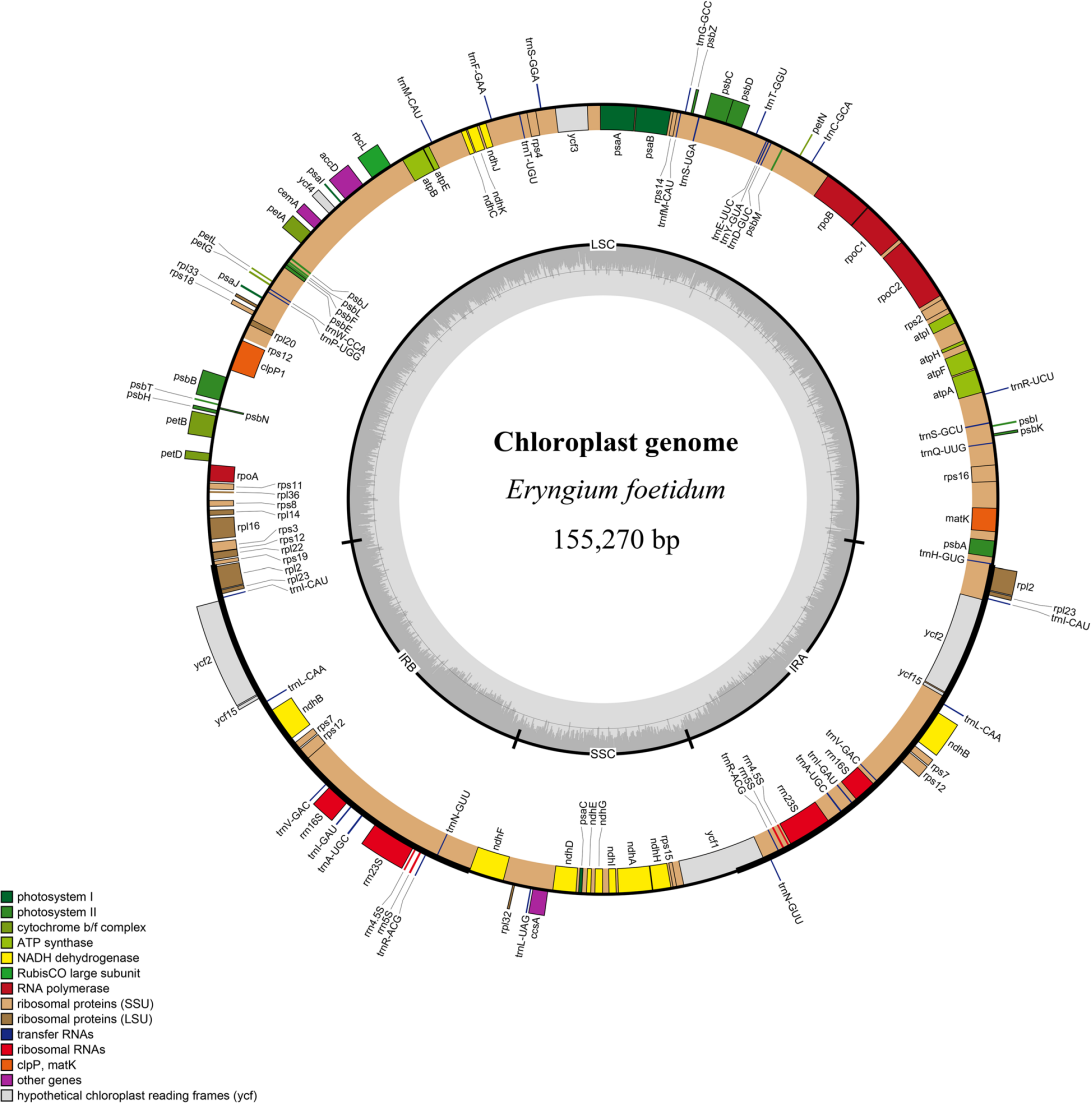
A circular gene map of one newly sequenced chloroplast genomes of *Eryngium foetidum*. Genes shown outside are transcribed clockwise, and inside the circle are transcribed counterclockwise. Genes are color-coded to distinguish different functional groups. The dark grey and the light grey plots in the inner circle correspond to the GC content and AT content, respectively

All four newly sequenced *Sanicula* cp genomes here encoded 103 unique genes, including 79 unique protein-coding genes (PCGs), 20 unique tRNA genes and four unique rRNA genes, and 23 of these were duplicated, with a total of 126 genes (Table 1; * showing the new chloroplast genomes reported in this study). 13 genes contain one (*atp*F, *ndh*A, *ndh*B, *pet*B, *rpl*16, *rpl*2, *rpo*C1, *rps*16, *trn*A-UGC, *trn*I-GAU) or two (*clp*P1, *rps*12, *ycf*3) introns, and two of these were tRNA genes (Table 2, Fig. 1). The cp genome contained coding regions ranging from 55.92% to 56.07% and non-coding regions ranging from 43.93% to 44.08%, including both intergenic spacers and introns (Table 2). They were divided into four categories, consisting of photosynthesis, self-replication, other genes, and function unknown genes (Table 2). The length of *Eryngium foetidum* L. was 155,270 bp, consisting of a LSC region of 85,874 bp, an SSC region of 17,074 bp, and a pair of inverted repeats region of 26,161 bp (Fig. 2). The overall GC content was 38.13%. It contained 127 genes, including 86 PCGs, 33 tRNA genes and 8 rRNA genes (Table 1; ^(a)^ showing the new chloroplast genomes reported in this study), and divided into four categories, consisting of photosynthesis, self-replication, other genes, and function unknown genes (Table 2).

**Table 2:** List of annotated genes in the chloroplast genomes of four newly sequenced *Sanicula* taxa and one sample of *Eryngium foetidum*

Category	Gene group	Gene name
Photosynthesis	Subunits of photosystem I	*psa*A, *psa*B, *psa*C, *psa*I, *psa*J
	Subunits of photosystem II	*psb*A, *psb*B, *psb*C, *psb*D, *psb*E, *psb*F, *psb*H, *psb*I, *psb*J, *psb*K, *psb*L, *psb*M, *psb*N, *psb*T, *psb*Z
	Subunits of NADH dehydrogenase	*ndh*A*, *ndh*B*^(2)^, *ndh*C, *ndh*D, *ndh*E, *ndh*F, *ndh*G, *ndh*H, *ndh*I, *ndh*J, *ndh*K
	Subunits of cytochrome b/f complex	*pet*A, *pet*B*, *pet*D, *pet*G, *pet*L, *pet*N
	Subunits of ATP synthase	*atp*A, *atp*B, *atp*E, *atp*F*, *atp*H, *atp*I
	Large subunit of rubisco	*rbc*L
Self-replication	Proteins of large ribosomal subunit	*rpl*14, *rpl*16*, *rpl*2*^(2)^, *rpl*20, *rpl*22, *rpl*23^(2)^, *rpl*32, *rpl*33, *rpl*36
	Proteins of small ribosomal subunit	*rps*11, *rps*12**^(2)^, *rps*14, *rps*15, *rps*16*, *rps*18, *rps*19, *rps*2, *rps*3, *rps*4, *rps*7^(2)^, *rps*8
	Subunits of RNA polymerase	*rpo*A, *rpo*B, *rpo*C1*, *rpo*C2
	Ribosomal RNAs	*rrn*16S^(2)^, *rrn*23S^(2)^, *rrn*4.5S^(2)^, *rrn*5S^(2)^
	Transfer RNAs	*trn*A-UGC*^(2)^, *trn*C-GCA, *trn*D-GUC, *trn*E-UUC, *trn*F-GAA, *trn*G-GCC, *trn*H-GUG, *trn*I-CAU^(2)^, *trn*I-GAU*^(2)^, *trn*L-CAA^(2)^, *trn*L-UAG, *trn*M-CAU, *trn*N-GUU^(2)^, *trn*P-UGG, *trn*Q-UUG, *trn*R-ACG^(2)^, *trn*R-UCU, *trn*S-GCU, *trn*S-GGA, *trn*S-UGA, *trn*T-GGU^▲^, *trn*T-UGU, *trn*V-GAC^(2)^, *trn*W-CCA, *trn*Y-GUA, *trnf*M-CAU
Other genes	Maturase	*mat*K
	Protease	*clp*P1**
	Envelope membrane protein	*cem*A
	Acetyl-CoA carboxylase	*acc*D
	c-type cytochrome synthesis gene	*ccs*A
Genes of unknown function	Conserved hypothetical gene	*ycf*1, *ycf*15, *ycf*2^(2)^, *ycf*3**, *ycf*4

Notes: Gene ^▲^ indicates only in *Eryngium foetidum*; Gene* indicates gene with one introns; Gene** indicates gene with two introns; Gene(2) indicates number of repeat units is 2

### Inverted repeats expansion, contraction, and interspecific comparison

**Fig. 3:**
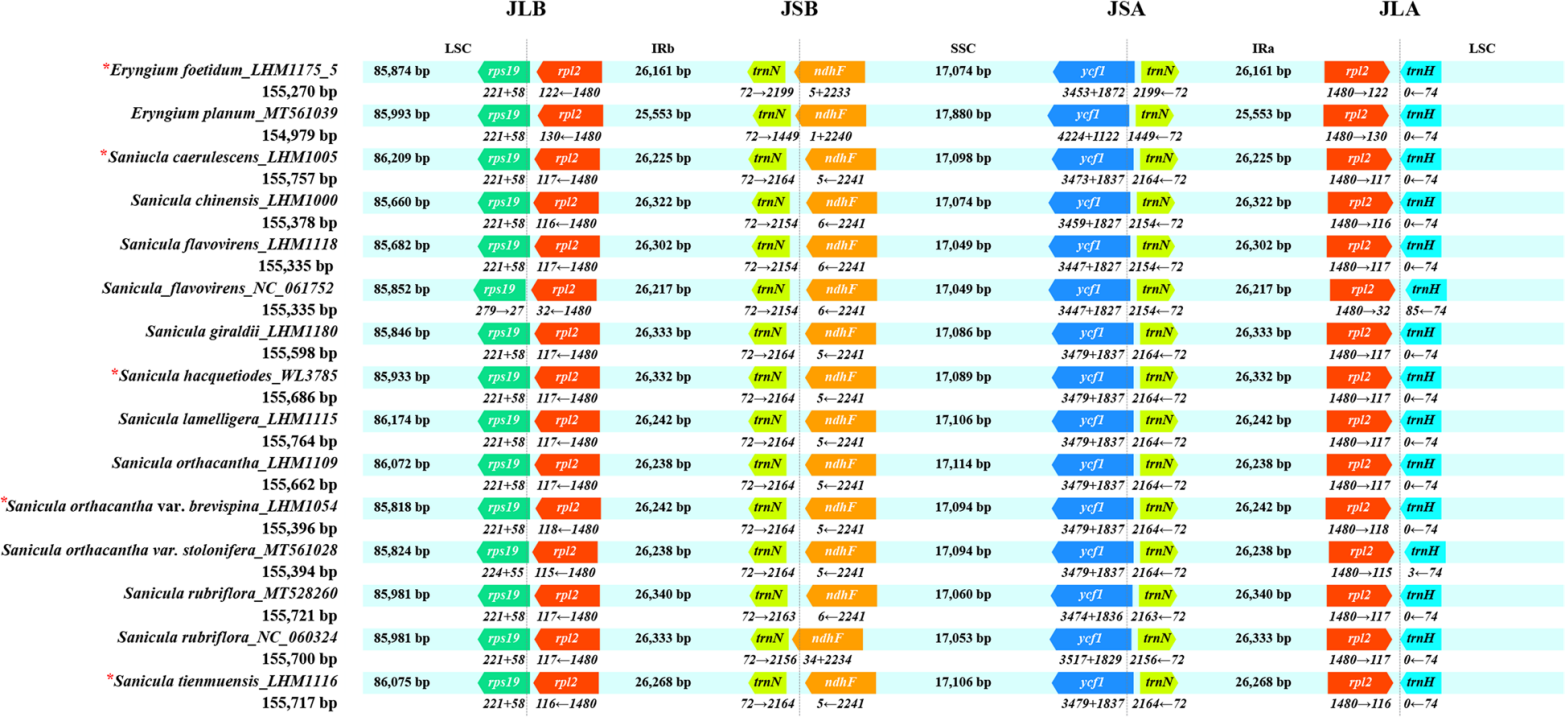
Comparison of the SC/IR junctions among the 15 chloroplast genomes, including 13 *Sanicula* and two *Eryngium* chloroplast genomes. JLA indicates LSC/IRa boundary; JSA indicates SSC/IRa boundary; JSBindicates SSC/IRb boundary; JLB indicates LSC/IRb boundary

In total, we analyzed and compared 15 cp genomes’ IR/LSC and IR/SSC boundary structures (including four *Sanicula* and one *Eryngium* samples from GenBank, four newly sequenced chloroplast genomes of *Sanicula* and one newly of *Eryngium*; Fig. 3, * showing the new chloroplast genomes reported in this study). The IRb/LSC boundary was located within the *rps*19 gene (with the 5′ end of the *rps*19 located in the IRb region while 3′ end located in the LSC), except in *S. flavovirens* sample (NC_061752), with an expansion length of 55 or 58 bp. The IRa/SSC boundary was in the *ycf*1 gene (the 5′ end of the *ycf*1 located in the IRa region while the 3′ end located in the SSC), with spanned 1122–1872 bp in the IRa region. The IRb/SSC boundary obviously varied: three samples were located within *ndh*F, with expanded 1–34 bp to the IRb region, while other 12 samples with 5 or 6 bp away from the IRb/SSC boundary.

**Fig. 4:**
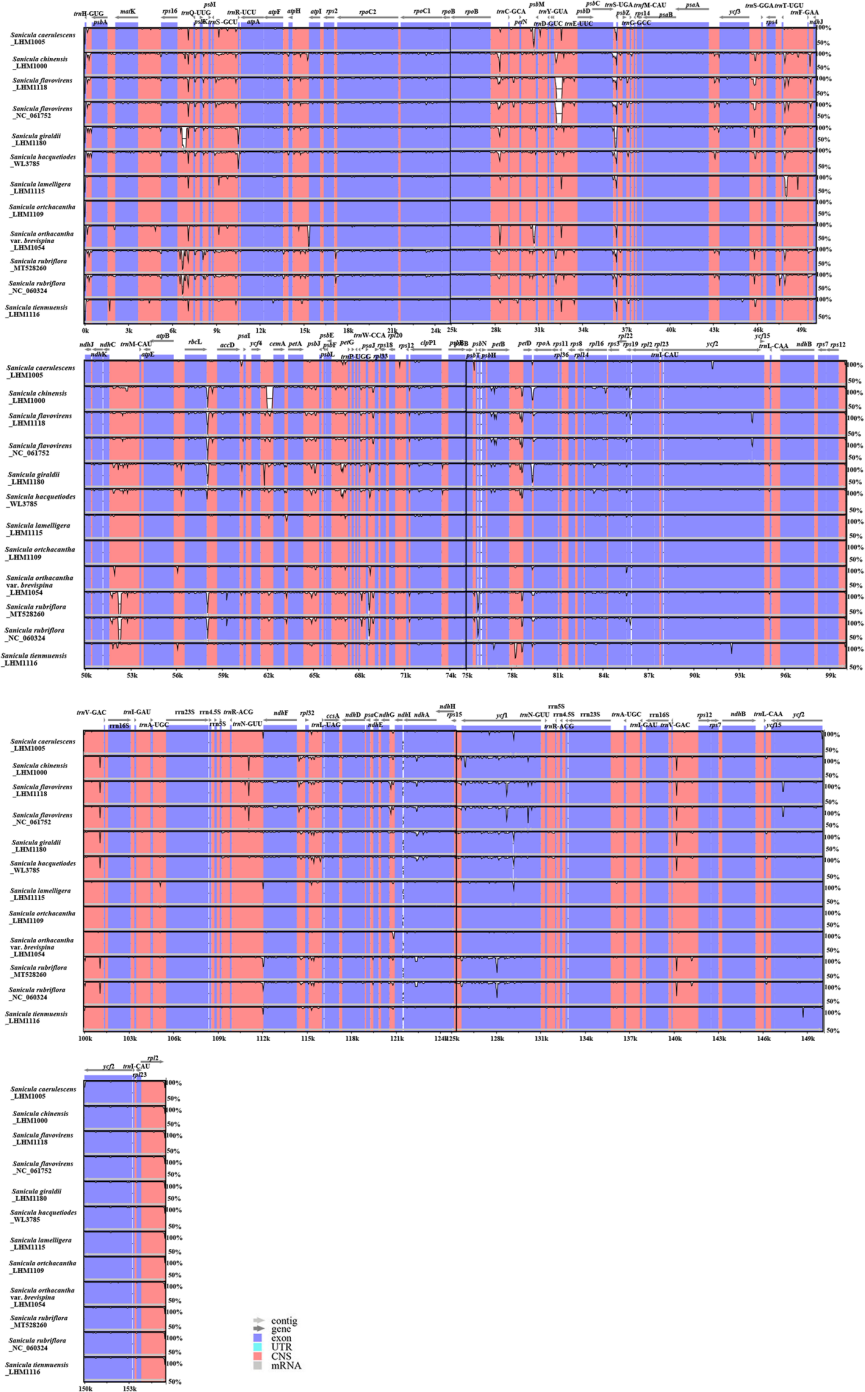
Plots of percent sequence identity of the chloroplast genomes of 12 *Sanicula* taxa with *S. orthacantha* var. *stonifera* (NCBI accession no. MT561028) as a reference

The mVISTA result showed that the non-coding regions were more variable than the coding regions, the LSC and SSC regions had higher level of sequence divergence than the two IR regions, and intergenic spacers (IGS) regions were the most divergent regions (Fig. 4). The highly divergent regions among the 13 chloroplast genomes occurred in 19 of the intergenic spacers, 17 in the LSC regions, including *atp*H-*atp*I, *ndh*C-*trn*M, *pet*B-*pet*D, *pet*D-*rpo*A, *pet*N-*psb*M, *psa*J-*rpl*33, *rbc*L-*acc*D, *rpo*B-*trn*C, *rps*16-*trn*Q, *trn*E-*psb*D, *trn*F-*ndh*J, *trn*H-*psb*A, *trn*S-*psb*Z, *trn*S-*trn*R, *trn*T-*trn*F, *ycf*3-*trn*S, *ycf*4-*cem*A; and one in the boundary between IRa and SSC region: *trn*N-*ndh*F; one in IR regions: *trn*V-*rps*12. Apart from these regions, one coding region *ycf*1 also showed high sequence variation (Fig. 4).

**Fig. 5:**
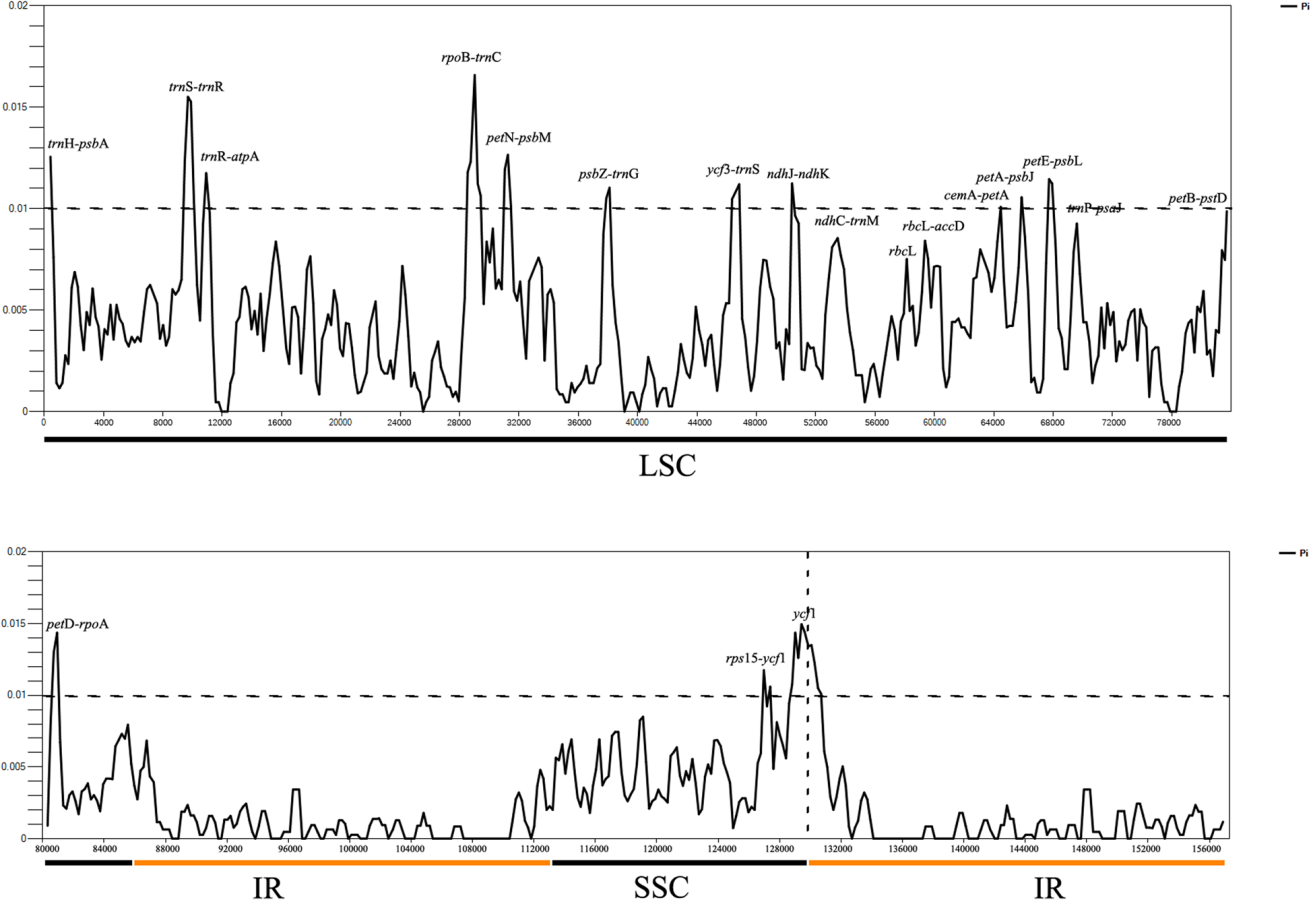
The nucleotide diversity of the whole chloroplast genomes of the 13 *Sanicula* taxa. LSC indicates large single copy region, IR indicates inverted repeat region, SSC indicates small single copy region

The value of nucleotide diversity (Pi) ranged from 0 to 0.01658, with average value of 0.003326 among the whole chloroplast (Fig. 5; Additional File 1: Table S1). The IR region were observed to have lower Pi value than LSC and SSC regions. The LSC region showed the highest nucleotide diversity (Pi = 0.01658), while the lowest Pi is in the IR regions (Pi = 0). 12 hypervariable sites with Pi more than 0.01 in LSC regions were screened (Fig. 5), namely *cem*A-*pet*A (Pi = 0.01009), *ndh*J-*ndh*K (Pi = 0.01124), *pet*A-*psb*J (Pi = 0.01059), *pet*D-*rpo*A (Pi = 0.01436), *pet*E-*psb*L (Pi = 0.01145), *pet*N-*psb*M (Pi = 0.01265), *psb*Z-*trn*G (Pi = 0.01103), *rpo*B-*trn*C (Pi = 0.01658), *trn*H-*psb*A (Pi = 0.0145), *trn*R-*atp*A (Pi = 0.01175), *trn*S-*trn*R (Pi = 0.01551), *ycf*3-*trn*S (Pi = 0.01047). Two hypervariable sites, *rps*15-*ycf*1 (Pi = 0.01128) and *ycf*1 (Pi = 0.01389), with high Pi value more than 0.01 in SSC regions were also screened in Fig. 5.

### Repeat structures and simple sequence repeats

**Fig. 6:**
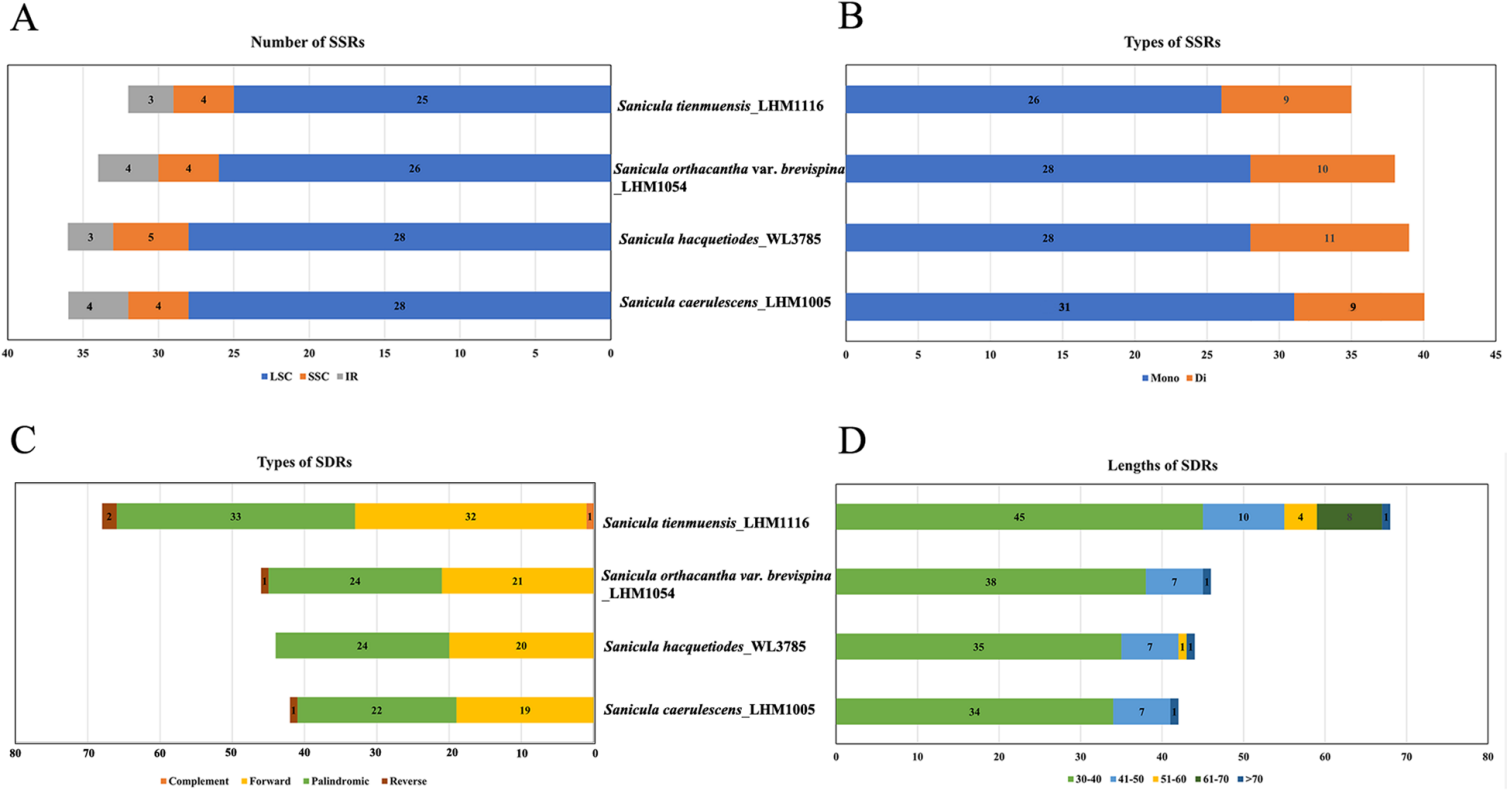
Statistics of repeats in four newly sequenced *Sanicula* taxa samples. **A**. Number of SSRs distributed in LSC, SSC and IR regions. **B**. Number of SSRs types. **C**. Number of four types SDRs. **D**. Number of different lengths of SDRs

The characteristics of Simple sequence repeats (SSRs) in four newly sequenced *Sanicula* cp genomes (*S. caerulescens*, *S. hacquetiodes*, *S. orthacantha* var. *brevispina* and *S. tienmuensis*) were analyzed, and the patterns of SSRs distribution were shown in Additional File 2: Table S2; Fig. 6A, B. A total of 40, 39, 38 and 35 SSRs loci were detected in these four newly sequenced *Sanicula* cp genome, respectively. The most abundant SSRs were A or T nucleotide repeats, which accounted for 28.95% to 35% of the total. SSRs were mainly distributed in LSC regions (76.47%–78.13%), and were significantly lower in the SSC (11.11%–13.89%) and IR (8.33%–11.76%) regions. Furthermore, they were only having mono- and di-nucleotide repeats. Among them, mono-nucleotide repeats were the most common SSR, accounting for 77.5%, 71.79%, 73.68% and 74.29% respectively, followed by 22.5%, 28.21%, 26.32%, and 25.71% in di-nucleotide repeats.

The REPuter screening discovered 42 to 68 dispersed repeats of 30 bp or longer among the four newly sequenced *Sanicula* cp genomes examined (Additional File 3: Table S3; Fig. 6C, D). The number of categories and the total number in repeats of *S. tienmuensis* (4; 68) were higher than *S. caerulescens* (3; 42), *S. hacquetiodes* (2; 44), and *S. orthacantha* var. *brevispina* (3; 46) (Fig. 6C). Only one, two and one reverse repeats were found in *S. caerulescens*, *S. tienmuensis* and *S. orthacantha* var. *brevispina*, respectively, while no reverse repeats was discovered in *S. hacquetiodes*. The complement repeat accounted for one only in *S. tienmuensis* (Fig. 6C). Among these four newly sequenced *Sanicula* cp genomes, the number of repeats with length between 30–40 bp exceeded those with lengths of  41–50 bp, 51–60 bp, 61–70 bp and over 70 bp (Fig. 6D).

### Statistics of codon usage

**Fig. 7:**
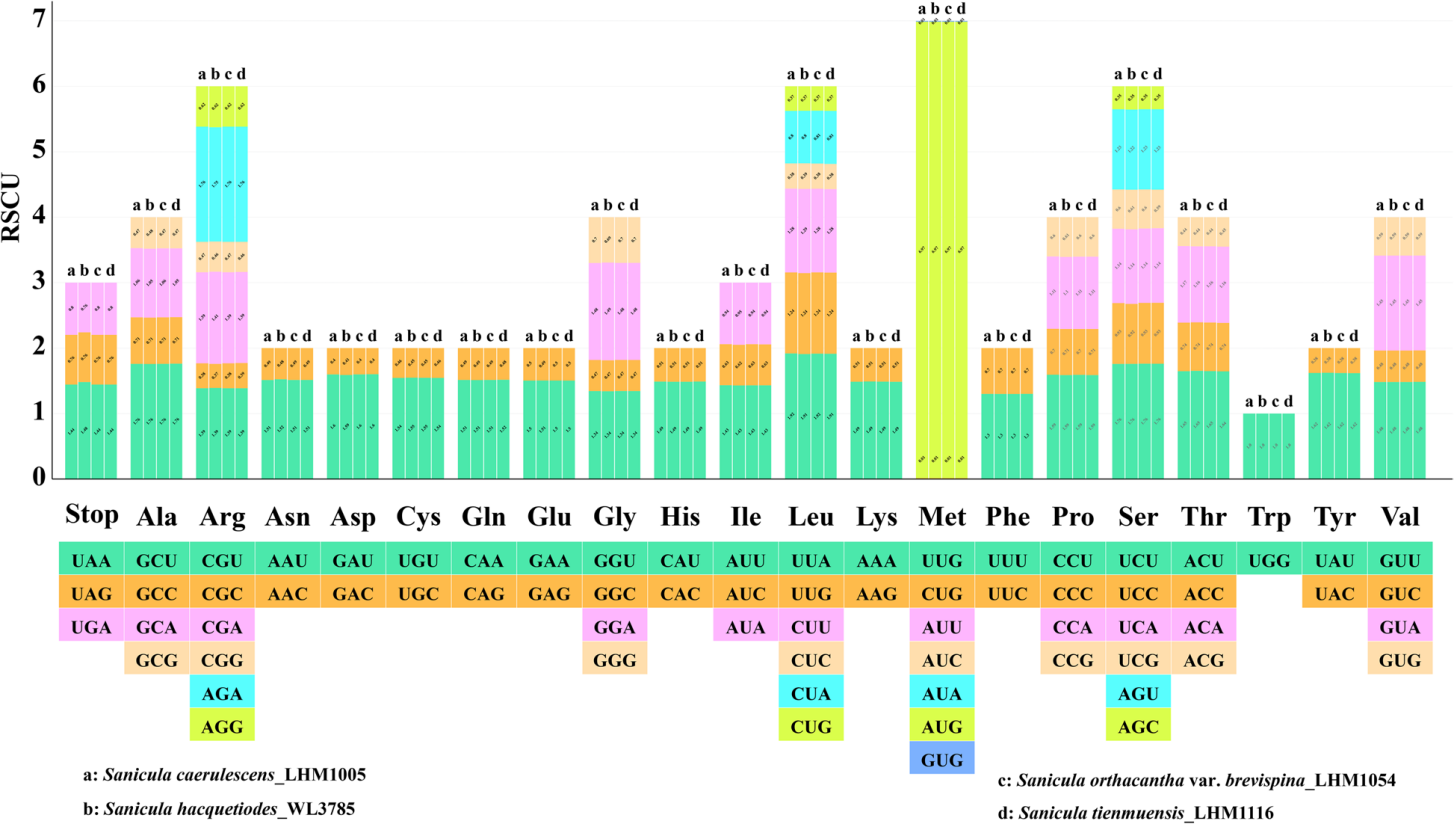
Codon content of 20 amino acids and stop codons in *Sanicula caerulescens* (**a**), *S. hacquetiodes* (**b**), *S. orthacantha* var. *brevispina* (**c**) and *S. tienmuensis* (**d**)

According to the codon usage analysis, the total sequence sizes of the PCGs were 67,857–67,863 bp in the four newly sequenced *Sanicula* taxa genomes; 22,673–22,690 codons were encoded (Additional File 4: Table S4). Leucine encoded with the maximum number of codons ranged from 2382 to 2390, followed by isoleucine, with the number of codons ranged from 1909 to 1918. Cysteine was the least with 237–239. The relative synonymous codon usage (RSCU) values varied slightly among the four newly sequenced *Sanicula* genomes (Fig. 7). Thirty-two codons were used frequently with RSCU ≥ 1 and 34 codons used less frequently with RSCU < 1. AUG showed a preference in all the four cp genomes. The frequency of use for the codon UGG, encoding the tryptophan (Trp), showed no bias (RSCU = 1).

### Phylogenetic analysis

**Fig. 8:**
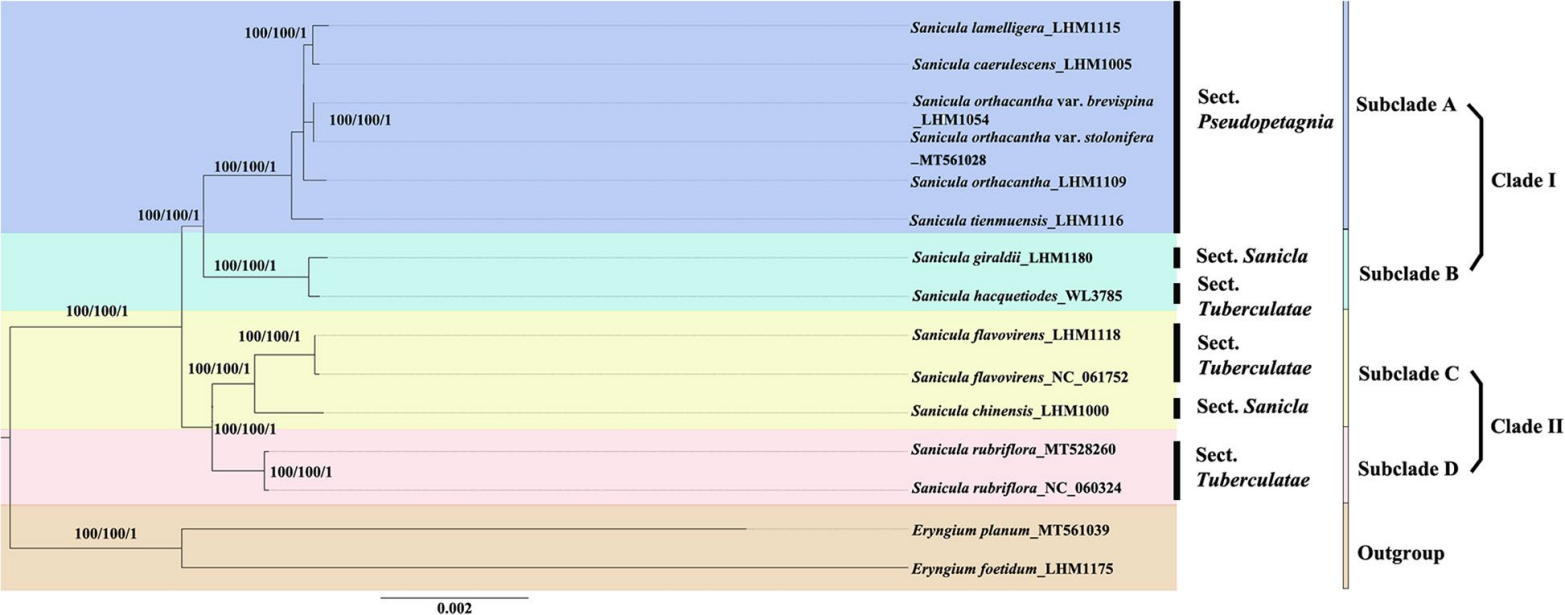
Phylogenetic tree based on complete cp genomes resulting from ML, MP and BI analysis of 13 *Sanicula* samples and two *Eryngium* species as references based on concatenation of 79 unique coding genes. The bootstrap support values and posterior probability values are displayed on the branches in the order ML/MP/BI, and values less than 50/50/0.5 are not shown

A total of three datasets, including the whole cp genomes sequences (Additional File 5: Fig. S1A), concatenation of 126 unique IGS regions (Additional File 5: Fig. S1B), concatenation of the unique 79 unique PCGs regions (Fig. 8) were constructed to investigate the phylogenetic relationships among 13 *Sanicula* taxa, with *Eryngium planum* L. and *E. foetidum* as outgroup taxa. By using the maximum likelihood (ML) method, three phylogenetic trees were built based on the three respective datasets, which exhibited highly concordant between one another. Therefore, only the ML topology of concatenation of the 79 unique PCGs regions among 13 *Sanicula* taxa, which also by using the Bayesian inference (BI) and Maximum Parsimony (MP) analyses, were shown here with the ML/MP/BI support [bootstrap support (bs) / bs / posterior probability (pp)] values added at each node with only slight differences (Fig. 8).

Our analyses confirmed that the genus *Sanicula* was monophyletic with strongly supported. Two major clades (clade I and II) were resolved within the monophyletic genus. The clade I comprised two fully supported subclades (A and B). Subclade A was consistent with Sect. *Pseudopetagnia* Wolff. Subclade B contained two species belonging to two different sections in the genus *Sanicula*, namely sect. *Sanicla* DC. and Sect. *Tuberculatae* Drude. Clade II divided into two subclades (C and D) with fully supply supported. Subclade C included three samples representing *S. flavovirens* and *S. chinensis*, respectively. However, they belonged to two different sections, i.e., Sect. *Tuberculatae* and Sect. *Sanicla*. Subclade D contained two samples representing *S. rubriflora* in Sect. *Tuberculatae*. These results showed that Sect. *Tuberculatae* and *Sect. Sanicla* were not natural monophyletic sections.

A total of 20 highly divergent regions (*atp*H-*atp*I, *ndh*C-*trn*M, *pet*B-*pet*D, *pet*D- *rpo*A, *pet*N-*psb*M, *psa*J-*rpl*33, *rbc*L-*acc*D, *rpo*B-*trn*C, *rps*16-*trn*Q, *trn*E-*psb*D, *trn*F-*ndh*J, *trn*H-*psb*A, *trn*N-*ndh*F, *trn*S-*psb*Z, *trn*S-*trn*R, *trn*T-*trn*F, *trn*V-*rps*12, *ycf*3-*trn*S, *ycf*4-*cem*A and *ycf*1) and concatenation of 20 highly divergent regions were also evaluated for phylogenetic analysis in our study (Additional Files 6, 7, 8, 9, 10, 11: Figs. S2–S7). Three molecular fragments, including *trn*E-*psb*D (Additional File 8: Fig. S4B), *trn*S-*trn*R (Additional File 9: Fig. S5C) and the concatenation regions (Additional File 11: Fig. S7), yielded similar topological results. However, compared to the three topological trees constructed by the whole cp genomes sequences (Additional File 5: Fig. S1A), concatenation of 126 unique IGS regions (Additional File 5: Fig. S1B), concatenation of 79 unique PCGs regions (Fig. 8), the supporting values were different observed from the nodes based on different sequences dataset. For example, the nodes in clades I derived from the dataset of *trn*E-*psb*D and *trn*S-*trn*R both showed strong supports (bs = 89.9% and bs = 85%; Additional Files 8, 9: Figs. S4B, S5C) lower than those from concatenation of 79 unique PCGs regions, whole cp genomes and concatenation of 126 unique IGS regions (bs = 100%, 100%, 99.4%; Fig. 8; Additional File 5: Fig. S1A, B). Additionally, the concatenation of 20 highly divergent regions had nodes strong supports in clades I and II (Additional File 11: Fig. S7). These results indicated a well resolution of the whole complete cp genomes, concatenations of PCGs regions and IGS regions as well as the concatenations of highly divergent regions compared to the single divergent region, which may serve as a reliable proof to reconstruct the phylogenetic relationship in *Sanicula*.

## Discussion

### The chloroplast genomic features, sequence variation and the potential molecular markers in *Sanicula*

The genus of *Sanicula* L. could be easily distinguished by basal leaves orbicular, rounded-cordate or cordate-pentagonal, usually palmately lobed; flowers polygamous, umbels in racemous, cymous or corymbose inflorescences from other genera of Subfam. Saniculoideae in Apiaceae. However, to understand the taxonomy and phylogenetic relationships in *Sanicula* had been particularly difficult based on its varied morphological characters in rhizomes, leaves, inflorescences and fruits. As a result, the previously reported chloroplast genomes of certain *Sanicula* species, which have been associated with ambiguous or incorrect information and potential misidentifications, were not included in our analysis, including *S. astrantiifolia*, *S. chinensis*, *S. giraldii*, *S. lamelligera*, *S. orthacantha*. In this study, 13 *Sanicula* genomes (including four newly sequenced, five re-annotated, and four previously reported) representing nine species, one variety and two *Eryngium* species were used to clarify the phylogenetic relationship.

The structure, gene orders and GC content were highly conserved and nearly similar in the samples of *Sanicula* analyzed here, and were also identical to other cp genomes in other genera of Apiaceae and other angiosperms [2, 17, 20, 21, 23–26]. The size of the 13 cp genomes varied from 155,335 (*S. flavovirens*; NC_061752 and OP703176) to 155,764 bp (*S. lamelligera*; OP703174) (Table 1). The *Sanicula* cp genomes sequenced here all contained total 126 genes (including 103 unique genes) with the total GC content being 38.16% or 38.25% (Table 1). However, some species were found to contain different numbers of genes in different samples, for examples, *S. flavovirens* (NC_061752), *S. orthacantha* var. *stolonifera* (MT561028), *S. rubriflora* (MT528260) and *S. rubriflora* (NC_060324) were reported to contain 129, 133, 133, 130 genes, respectively, whereas all annotated here with 126 genes. To eliminate the influences of references used and annotation software, the 13 samples were re-annotated using Plastid Genome Annotator (PGA) and Geneious Prime 2020.0.5 with *Heteromorpha arborescens* (NC_053554), and their tRNA genes were verified by tRNA-SE. Unexpectedly, we examined all the 13 sequences re-annotated only with 126 genes and did not find any gene loss in this study (Table 2).

The variation of length in cp genomes usually hinted the IR region expansions, which were useful in evolutionary studies in some taxa [23–25, 31–33]. However, our findings indicated that there were only minor variations observed in the cp genomes of *Sanicula* examined, with no significant expansions or contractions. Among 13 *Sanicula* cp genome, the length of the IR region varied, with *S. rubriflora* (26,340 bp; MT528260) exhibiting the longest IR length, while *S. flavovirens* (26,217 bp; NC_061752) had the shortest. Only the *ndh*F gene, with an expansion length of 34 bp for *S. rubriflora* expanded to the IRb region, for remaining 12 *Sanicula* samples were entirely located within the SSC region. And the *rps*19 gene with contractions length of 27 bp away from IRb region only in *S. flavovirens* (NC_061752). These results were also similar to the expansion in the cp genome of other species in Apiaceae among IR regions [18, 34].

Genome composition, including the factors such as gene sequence length, tRNA abundance, GC distribution position, and other related features, along with natural selection, were the two major factors affecting codon usage bias [27, 28, 35–37]. The total number of 63 codons present across the *Sanicula* cp genomes encoding 20 amino acids and codon usage was biased towards A or U at the third codon position, which was in consistent with other Apiaceae taxa [2, 23, 29, 30].

Many works proved that the variation of SSRs in cp genomes were widely used in population genetic studies, species identification and evolutionary relationship [26, 34, 38]. In this study, the characteristics of SSRs and short dispersed repeats (SDRs) were also similar among these *Sanicula* cp genomes. Our results suggested that the mononucleotide (A/T) account for the most abundant repeat type, and the IR regions contained less SDRs and SSRs than LSC and SSC regions, which were consistent with the analyses in other Apiaceae taxa [2, 18, 34]. Therefore, this indicated that the LSC and SSC regions possessed high level of nucleotide variability, which could be used as potential polymorphic molecular markers for identification, phylogeny, and evolutionary study in *Sanicula*.

Our analysis of nucleotide diversity revealed that 19 IGS within the non-coding regions (17 in the LSC regions, including *atp*H-*atp*I, *ndh*C-*trn*M, *pet*B-*pet*D, *pet*D-*rpo*A, *pet*N-*psb*M, *psa*J-*rpl*33, *rbc*L-*acc*D, *rpo*B-*trn*C, *rps*16-*trn*Q, *trn*E-*psb*D, *trn*F-*ndh*J, *trn*H-*psb*A, *trn*S-*psb*Z, *trn*S-*trn*R, *trn*T-*trn*F, *ycf*3-*trn*S, *ycf*4-*cem*A; and one in the boundary between IRa and SSC region: *trn*N-*ndh*F; one in IR regions: *trn*V-*rps*12.), as well as one coding region (*ycf*1), exhibited high levels of divergence in *Sanicula* (Fig. 5). This finding was consistent with the diverse patterns typically observed in angiosperms, where nucleotide diversity tended to be higher in non-coding regions compared to coding regions [39]. However, among these variable sequences, the only one chloroplast marker, *rps*16-*trn*Q, which was applied in phylogenetic utility for subfam. Saniculoideae of Apiaceae [8, 13]. In this study, 18 hypervariable regions, including *atp*H-*atp*I, *ndh*C-*trn*M, *pet*B-*pet*D, *pet*D-*rpo*A, *pet*N-*psb*M, *psa*J-*rpl*33, *rbc*L-*acc*D, *rpo*B-*trn*C, *rps*16-*trn*Q, *trn*F-*ndh*J, *trn*H-*psb*A, *trn*S-*psb*Z, *trn*T-*trn*F, *ycf*3-*trn*S, *ycf*4-*cem*A, *trn*N-*ndh*F, *trn*V-*rps*12 and *ycf*1, had contributed to a certain degree of confusion in the topology (Additional Files 6, 7, 8, 9, 10: Figs. S2–S6). However, the phylogenetic analysis based on *trn*E-*psb*D (Additional File 8: Fig. S4B), *trn*S-*trn*R (Additional File 9: Fig. S5C) and the concatenation regions (Additional File 11: Fig. S7), resulted in similar topological trees as the whole cp genomes sequences (Additional File 5: Fig. S1A), concatenation of 126 unique IGS regions (Additional File 5: Fig. S1B), concatenation of 79 unique PCGs regions (Fig. 8) could well differentiate the two clades in *Sanicula*. Therefore, the two new highly variable chloroplast marker, *trn*E-*psb*D and *trn*S-*trn*R, might be the promising potential molecule makers in phylogeny reconstruction.

### Phylogenetic analysis

Based on the conservatism and heritance, cp genomes were effective in inferring the phylogenetic relationships at various taxonomic levels [40]. In our phylogenetic analysis using the 79 unique coding genes, the monophyly and infrageneric classification in Chinese *Sanicula* were investigated. The status of *S. chinenesis*, *S. hacquetiodes*, and *S. giraldii* was also re-evaluated.

The systematics of Chinese *Sanicula* had been discussed based on molecular phylogenetic research [2, 8, 13, 14, 17, 21, 41] and morphological study [6, 41]. The phylogenetic trees obtained here were found to be consistent with those reported by Vargas et al*.* [41] using nuclear ribosomal DNA internal transcribed spacer (ITS). For instance, *S. lamelligera* and *S. orthacantha* formed a monophyletic lineage within Sect. *Pseudopetagnia*, as proposed by Wolff [12]. However, the other two sections, including Sect. *Tuberculatae* and Sect. *Sanicla*, defined by Drude [42] and de Candolle [43], and relationship among species within these sections suggested by Shan & Constance [6] were not supported.

The samples in clade I had involucellate bracteoles small and shorter than umbellets, fertile flowers 1 to 3 per umbellule, fruits characterized by tuberculate, prickly, lamellate, squamosa [3, 6]. Two monophyletic subclades, including subclade A and B, were resolved in this clade. Subclade A contained six taxa belonged to Sect. *Pseudopetagnia*, which characterized by involucellate bracteoles small and shorter than umbellets, fertile flowers only one per umbellule and fruits squamosa, lamellate or with straightly spiculate spicules. Subclade B included two species, *S. giraldii* of Sect. *Sanicla* and *S. hacquetiodes* of Sect. *Tuberculatae*, with fully supported nested within clade I. Furthermore, Shan & Constance [6] noted that the noteworthy relationship of *S. hacquetiodes* with Sect. *Pseudopetagnia* based on the similar morphological characters, including the presence of generally one fertile flowers and tendency towards a subracemose inflorescence structure. However, two species in subclade B lack of a consistent morphological synapomophy except for involucellate bracteoles small and shorter than umbellets.

Species in clade II could be easily distinguished from taxa in clade I by involucellate bracteoles often longer than umbellets in flowering and fertile flowers often 3 or more per umbellule [3, 6]. In this study, clade II showed that *Sanicula chinensis* of Sect. *Sanicla* was nested within Sect. *Tuberculatae* (including *S. flavovirens* [5] and *S. rubriflora*), which formed the sister group of *S. flavovirens* in well supported. In accordance with previous publications [2], it was suggested that *S. chinensis* and *S. orthacantha* formed a strong-supported sister group to *S. lamelliegera*. However, upon conducting a critical examination, the sample of *S. chinensis* (MK208987) used in the referenced paper [2] might be a misidentification. Thus, it was advisable to exercise caution when utilizing the sequence in future, as the reliability of the results obtained from this data remained debatable. Within clade II, two subclades, namely subclades C and D, were fully supported. Morphologically, species belonging to subclade D exhibited considerably longer involucellate bracteoles length compared to those in subclade C. Subclade C encompassed two species, formerly assigned to two sections (Sect. *Tuberculatae* and Sect. *Sanicla*), which could be easily distinguished by flower characteristics. Subclade D contained two samples of *S. rubriflora*, a species that was previously classified within Sect. *Tuberculatae*. It was suggested by Shan & Constance [6] that *S. rubriflora* may potentially represent an ancestral species within the genus *Sanicula*. Notably, *S. rubriflora* was more closely related to *S. flavovirens* in having numerous staminate flowers with pedicels and base tuberculate fruits with stout uncinate prickles above, rather than to *S. chinensis*, which had bits of staminate flowers with deciduous pedicel and fruit only covered with uncinate prickles. Thus, to better address the issue of inconsistent classification of chloroplast (cp) genomes and morphology more effectively, it was crucial to obtain additional samples from other species in *Sanicula*. Particularly, the ones within Sect. *Tuberculatae* and Sect. *Sanicla* should be included to validate their placement within the Chinese *Sanicula*.

Taxonomic inconsistencies in the delimitation of taxa continue to pose a challenge within the genus *Sanicula*. For instance, *S. orthacantha* var. *brevispina* was treated as a synonym of *S. orthacantha* var. *orthacantha* by Shan & Constance [6] and Hiroe [7], while had been reinstated as a distinct variety by Liou [11], Fu [44], Wang [45], Sheh & Phillippe [3] and Pimenov [4]. Additionally, *S. orthacantha* var. stolonifera was only recognized by Sheh & Phillippe (2005) along with its publication. In previous study [10], we found that *S. orthacantha* var. *orthacantha* definitely differed from *S. orthacantha* var. *brevispina* only by short rhizome, oblique rootstock bearing elongated, fibrous roots, sometimes fleshy stoloniferous (vs. slender, elongate and lignified nodes stoloniferous), and *S. orthacantha* var. *stolonifera* was a synonym of *S. orthacantha* var. *brevispina.* In this study, the results strongly supported the clustering of *S. orthacantha* var. *brevispina* with *S. orthacantha* var. *stolonifera*, while weakly supporting the relationship between *S. orthacantha* var. *orthacantha* and *S. orthacantha* var. *brevispina.* Thus, our findings provided substantial support for the treatment proposed by Li et al. [10].

## Conclusion

This study reports four newly sequenced complete cp genomes of *Sanicula* taxa, i.e. *S. caerulescens*, *S. hacquetiodes*, *S. orthacantha* var. *brevispina*, *S. tienmuensis*, following the analysis of SSRs, codon usage, IR boundaries, sequence divergence estimates with other nine Chinese *Sanicula* samples. Insight into the interspecific relationships in the 11 Chinese *Sanicula* taxa (including 13 samples) verifies, in some degree, the traditional system based on morphology analysis. These results will help to understand the relationship and evolution clearly in *Sanicula* at the molecular level and benefit their identification, utilization, and protection as herbal medicinal genus.

## Methods

### Plant materials, DNA extraction and sequencing of the chloroplast genomes

Eight species and one variety of *Sanicula* L. and one species of *Eryngium* L. were collected from field observation in China (Table 3). Fresh and healthy leaf tissues were collected in field and stored in silica gel. Voucher specimens were deposited in the herbarium of Institute of Botany, Jiangsu Province and Chinese Academy of Sciences (NAS), and their deposition numbers were listed in the Additional file 12: Table S5. In addition, four complete chloroplast genomes of *Sanicula* species (Table 1) and one of *Eryngium* species (Table 1) that publicly available in NCBI GenBank were downloaded with annotations.

**Table 3:** Collecting information for the nine taxa of *Sanicula* L. and one species of *Eryngium* L. sequenced in the study

Genus	Species	Locality	Voucher	Longitude/Latitude
*Eryngium* L	*E. foetidum*	Kunming, Yunnan	H.M. Li 1175 (NAS)	102° 42′ 34″/25° 2′ 47″
*Sanicula* L	*S. caerulescens*	Jinyun Mountain, Beibei, Chongqing	H.M. Li & W. Zhou 1005 (NAS)	106°23′18"/29°50′22"
	*S. hacquetiodes*	Dêqên County, Dêqên, Yunnan	L. Wang, H.M. Li & T. Li 3785 (NAS)	98° 45′ 37"/28° 4′ 23"
	*S. orthacantha* var.* brevispina*	Mount Emei, Emeishan, Sichuan	H.M. Li & W. Zhou 1054 (NAS)	103°19′57″/29°31′11″
	*S. tienmuensis*	Mount Tianmu, Lin'an County, Hangzhou, Zhejiang	H.M. Li & L. Zhao 1116 (NAS)	119°25′/30°20′
	*S. chinensis*	Linggu Temple, Nanjing, Jiangsu	H.M. Li & M. Chen 1000 (NAS)	118° 52′ 48″/32° 3′ 18″
	*S. flavovirens*	Mount Dapan,Pan'an, Jinhua, Zhejiang	H.M. Li & L. Zhao 1118 (NAS)	120°32′8"/28°58′51"
	*S. giraldii*	Near Laoyu River, Zhouzhi, Xi'an, Shaanxi	H.M. Li & C.F. Song 1180 (NAS)	113°12′40"/34°0′5"
	*S. lamelligera*	Mount Tianmu, Lin'an County, Hangzhou, Zhejiang	H.M. Li & W. Zhou 1115 (NAS)	30°19′59.49"/119°27′0.91"
	*S. orthacantha*	Mount Lu, Jiujiang, Jiangxi	H.M. Li, Y.S. Zhang & Y. Xu 1109 (NAS)	115°52′/29°26′

Total genomic DNA was extracted from silica-dried leaf tissues following a modified CTAB method [46]. DNA integrity was examined by electrophoresis in 1% (w/v) agarose gel, and concentration was measured using a NanoDrop spectrophotometer 2000 (Thermo Scientific; Waltham, MA, USA), then accurate quantifications were completed by Qubit 2.0. High-quality DNA libraries constructed and sequenced at Novogene Bioinformatics Technology Co., Ltd. (https://www.novogene.com/, accessed on March 2011 Tianjin, China). The strategy of Nova-PE150 was selected for high-throughput sequencing, with an insert size of 350 bp.

### Complete chloroplast genomes assembly and annotation

The clean data of sequencing were directly assembled using the GetOrganelle pipeline [47–49]. Bandage v.5.6.0 [50] was used to visualize and manually correct the assembly results. The annotation of the chloroplast genomes was performed in PGA program [51]. Manual correction of start/stop codons and intron/exon boundaries was performed in Geneious Prime 2020.0.5 [52]. All genome maps were drawn by Organellar Genome DRAW v.1.3.1 [53]. The annotated chloroplast genomes were deposited in GenBank (Table 1).

### Genome comparison, codon usage analyses and simple sequence repeat analysis

We applied MAFFT v7.490 [54] to align the total 13 cp genomes sequences (Table 1) for examining the divergence regions among *Sanicula* species. The aligned sequences were performed in Shuffle-LAGAN model via mVISTA program (http://genome.lbl.gov/vista/ mvista/submit.shtml) with the annotated cp genome sequence of S. orthacantha* var. *stolonifera (GenBank accession no. MT561028) as a reference genome. DnaSP v6 [55] was applied to examine the sequence divergence hotspots with conducting a sliding window analysis to calculate pi values among the cp genomes, with windows size of 600 bp and step size of 200 bp.

IRscope software was used for the 13 cp genome sequences to visualize their IR/SC boundaries. CodonW [56] was implemented to analysis the codon usage bias for all PCGs. SSRs were identified by Web-based simple sequence repeats finder MISA-web (https://www.webblast.ipk-gatersleben.de/misa/), with minimum numbers of 10 repeat units for mono-, 6 repeat units for di-, 5 repeat units for tri-, tetra-, penta-, and hexa-nucleotide SSRs. The maximum length of a sequence between two SSRs was set as 10. REPuter was implemented to detect the SDRs [57], including forward, reverse, complement and palindromic, with the following parameters: a maximal repeat size of 5000, a minimal repeat size of 30, and hamming distance of 3.

### Phylogenetic analysis

A total of 24 datasets, including the 13 complete cp genome sequences of *Sanicula*, concatenation of 126 unique IGS regions, concatenation of 79 unique PCGs regions, 20 highly divergent regions (*atp*H-*atp*I, *ndh*C-*trn*M, *pet*B-*pet*D, *pet*D-*rpo*A, *pet*N-*psb*M, *psa*J-*rpl*33, *rbc*L-*acc*D, *rpo*B-*trn*C, *rps*16-*trn*Q, *trn*E-*psb*D, *trn*F-*ndh*J, *trn*H-*psb*A, *trn*N-*ndh*F, *trn*S-*psb*Z, *trn*S-*trn*R, *trn*T-*trn*F, *trn*V-*rps*12, *ycf*3-*trn*S, *ycf*4-*cem*A, and *ycf*1) and concatenation of 20 highly divergent regions, with two *Eryngium* species (including one newly reported taxon), i.e. *E. planum* L. and *E. foetidum*, selected as outgroup taxa, were used for phylogenetic analysis. Additionally, phylogenetic analyses were performed using BI, ML and MP based on concatenation of 79 unique PCGs included in the final alignment. BI and MP analyses were conducted on the CIPRES Science Gateway website [58]. BI analyses were run with MrBayes on XSEDE version 3.2.7a [59]. Models were selected among model analyzed by MrBayes using Bayesian model choice criteria (nst = mixed, rates = gamma). MP analyses were run with PAUP on XSEDE version 4.a168 [31] using the heuristic search option with 1000 random sequence additions. ML phylogenetic analyses were performed in the IQ-tree program [32, 33] with auto substitution model and 1000 bootstrap replicates for evaluating the node support. FigTree v 1.4 (http://tree.bio.ed.ac.uk/ software/ fgtree/) was used to visualize the resulting trees.

### Supplementary Information

**Additional file 1: Table S1.** The nucleotide variability (Pi) of 13* Sanicula* taxa in whole chloroplast genomes.

**Additional file 2: Table S2.** The comparison of SSRs among four newly sequenced *Sanicula* taxa chloroplast genomes.

**Additional file 3: Table S3.** Comparison of dispersed repeats among four newly sequenced *Sanicula* taxa chloroplast genomes.

**Additional file 4: Table S4.** Codon usage and relative synonymous codon usage (RSCU) values of protein-coding genes of the four newly sequenced *Sanicula *chloroplast genomes.

**Additional file 5:Fig. S1.** Collecting information, voucher specimen and identification for the nine taxa of *Sanicula* L. and one species of *Eryngium* L. in the study.

**Additional file 6: Fig. S2.** Phylogenetic relationships of 13 *Sanicula* samples and two *Eryngium* species inferred from maximum likelihood (ML) analysis. A. The whole cp genome. B. Concatenation of 126 unique IGS regions.

**Additional file 7: Fig. S3** Phylogenetic relationships of 13 *Sanicula* samples and two *Eryngium* species inferred from maximum likelihood (ML) analysis. A. *atp*H-*atp*I. B. *ndh*C-*trn*M. C. *pet*B-*pet*D. D. *pet*D-*rpo*A.

**Additional file 8: Fig. S4.** Phylogenetic relationships of 13 *Sanicula* samples and two *Eryngium* species inferred from maximum likelihood (ML) analysis. A. *pet*N-*psb*M. B. *psa*J-*rpl*33. C. *rbc*L-*acc*D. D. *rpo*B-*trn*C.

**Additional file 9: Fig. S5.** Phylogenetic relationships of 13 *Sanicula* samples and two *Eryngium* species inferred from maximum likelihood (ML) analysis. A. *rps*16-*trn*Q.  B. *trn*E-*psb*D. C. *trn*F-*ndh*J. D.* trn*H-*psb*A.

**Additional file 10: Fig. S6.** Phylogenetic relationships of 13 *Sanicula* samples and two *Eryngium* species inferred from maximum likelihood (ML) analysis. A. *trn*N-*ndh*F. B. *trn*S-*psb*Z. C. *trn*S-*trn*R. D. *trn*T-*trn*F.

**Additional file 11: Fig. S7.** Phylogenetic relationships of 13 *Sanicula* samples and two *Eryngium* species inferred from maximum likelihood (ML) analysis. A. *trn*V-*rps*12. B. *ycf*3-*trn*S. C. *ycf*4-*cem*A. D. *ycf*1.

**Additional file 12: Table S5.** Phylogenetic relationships based on the concatenation of 20 highly divergent regions (*atp*H-*atp*I, *ndh*C-*trn*M, *pet*B-*pet*D, *pet*D-*rpo*A, *pet*N-*psb*M, *psa*J-*rpl*33, *rbc*L-*acc*D, *rpo*B-*trn*C, *rps*16-*trn*Q, *trn*E-*psb*D, *trn*F-*ndh*J, *trn*H-*psb*A, *trn*N-*ndh*F, *trn*S-*psb*Z, *trn*S-*trn*R,*trn*T-*trn*F, *trn*V-*rps*12, *ycf*3-*trn*S, *ycf*4-*cem*A, and *ycf*1) in 13 *Sanicula* samples and two *Eryngium* species inferred from maximum likelihood (ML) analysis.

## Data Availability

Ten annotated plastomes, including four newly sequenced *Sanicula* taxa and one newly sequenced *Eryngium* species have been submitted into NCBI (https://www.ncbi.nlm.nih.gov) with accession numbers: OP696651; OP703171-OP703179, respectively.

## Abbreviations

BI: Bayesian inference
bs: Bootstrap support
cp: Chloroplast
CTAB: Cetyl trimethylammonium bromide
IGS: Intergenic spacers
IRs: Inverted repeats
ITS: Internal transcribed spacer of ribosomal DNA
LSC: Large single-copy
ML: Maximum-likelihood
MP: Maximum parsimony
NCBI: National Center for Biotechnology
PCGs: Protein-coding genes
PGA: Plastid genome annotator
PP: Posterior probability
Pi: Nucleotide diversity/polymorphism
rRNA: Ribosomal RNA
RSCU: Relative synonymous codon usage
SDR: Short dispersed repeats
SSC: Small single-copy
SSR: Simple sequence repeat
tRNA: Transfer RNA
