# Supplementary material for: Complete chloroplast of four Sanicula taxa (Apiaceae) endemic to China: lights into genome structure, comparative analysis, and phylogenetic relationships
Source: BMC Plant Biol. 2023 Sep 21;23:444. doi: 10.1186/s12870-023-04447-w (PMC10512634; doi:10.1186/s12870-023-04447-w)
Supplement: Supplementary file 1 — Additional file 1: Table S1. The nucleotide variability (Pi) of 13 Sanicula taxa in whole chloroplast genomes. [file 12870_2023_4447_MOESM1_ESM.docx]

**Table S1**. The nucleotide variability (Pi) of 13 *Sanicula* taxa in whole chloroplast genomes.

| Window | Midpoint | Pi | Theta | S |
| --- | --- | --- | --- | --- |
| 1-725 | 418 | 0.01256 | 0.0145 | 27 |
| 307-925 | 625 | 0.00761 | 0.00859 | 16 |
| 522-1125 | 825 | 0.00141 | 0.00161 | 3 |
| 726-1325 | 1025 | 0.00115 | 0.00107 | 2 |
| 926-1525 | 1225 | 0.00141 | 0.00161 | 3 |
| 1126-1725 | 1425 | 0.00278 | 0.00269 | 5 |
| 1326-1951 | 1625 | 0.00235 | 0.00269 | 5 |
| 1526-2153 | 1850 | 0.00611 | 0.00537 | 10 |
| 1726-2362 | 2052 | 0.00688 | 0.00698 | 13 |
| 1952-2562 | 2262 | 0.00615 | 0.00591 | 11 |
| 2154-2762 | 2462 | 0.00423 | 0.00483 | 9 |
| 2363-2962 | 2662 | 0.00303 | 0.00322 | 6 |
| 2563-3162 | 2862 | 0.00491 | 0.00537 | 10 |
| 2763-3362 | 3062 | 0.00427 | 0.00537 | 10 |
| 2963-3562 | 3262 | 0.00607 | 0.00698 | 13 |
| 3163-3762 | 3462 | 0.00466 | 0.00537 | 10 |
| 3363-3962 | 3662 | 0.00419 | 0.00483 | 9 |
| 3563-4164 | 3862 | 0.00256 | 0.00322 | 6 |
| 3763-4364 | 4064 | 0.00406 | 0.0043 | 8 |
| 3963-4570 | 4264 | 0.0038 | 0.00376 | 7 |
| 4165-4773 | 4464 | 0.00526 | 0.00537 | 10 |
| 4365-4983 | 4673 | 0.00355 | 0.0043 | 8 |
| 4571-5183 | 4873 | 0.00526 | 0.00537 | 10 |
| 4774-5394 | 5083 | 0.00457 | 0.00537 | 10 |
| 4984-5594 | 5286 | 0.00432 | 0.00483 | 9 |
| 5184-5794 | 5494 | 0.0035 | 0.00483 | 9 |
| 5395-5994 | 5694 | 0.00321 | 0.00376 | 7 |
| 5595-6194 | 5894 | 0.00368 | 0.0043 | 8 |
| 5795-6394 | 6094 | 0.00342 | 0.00322 | 6 |
| 5995-6594 | 6294 | 0.00368 | 0.00322 | 6 |
| 6195-6826 | 6494 | 0.00346 | 0.00322 | 6 |
| 6395-7037 | 6726 | 0.00491 | 0.00537 | 10 |
| 6595-7253 | 6926 | 0.00603 | 0.00644 | 12 |
| 6827-7702 | 7137 | 0.00624 | 0.00644 | 12 |
| 7038-7903 | 7602 | 0.00534 | 0.00591 | 11 |
| 7254-8112 | 7802 | 0.00359 | 0.00483 | 9 |
| 7703-8312 | 8012 | 0.00427 | 0.00537 | 10 |
| 7904-8512 | 8212 | 0.00325 | 0.00322 | 6 |
| 8113-8749 | 8412 | 0.00368 | 0.00322 | 6 |
| 8313-8949 | 8641 | 0.00603 | 0.00591 | 11 |
| 8513-9150 | 8849 | 0.00577 | 0.00591 | 11 |
| 8750-9354 | 9049 | 0.00598 | 0.00644 | 12 |
| 8950-9570 | 9250 | 0.0065 | 0.00644 | 12 |
| 9151-9790 | 9470 | 0.01231 | 0.01182 | 22 |
| 9355-9991 | 9689 | 0.01551 | 0.01504 | 28 |
| 9571-10191 | 9890 | 0.01526 | 0.01504 | 28 |
| 9791-10401 | 10091 | 0.01034 | 0.0102 | 19 |
| 9992-10601 | 10301 | 0.00624 | 0.00591 | 11 |
| 10192-10801 | 10501 | 0.00449 | 0.00376 | 7 |
| 10402-11054 | 10701 | 0.00923 | 0.00967 | 18 |
| 10602-11256 | 10916 | 0.01175 | 0.01235 | 23 |
| 10802-11456 | 11154 | 0.00974 | 0.01074 | 20 |
| 11055-11656 | 11356 | 0.00393 | 0.0043 | 8 |
| 11257-11856 | 11556 | 0.00047 | 0.00054 | 1 |
| 11457-12056 | 11756 | 0.00047 | 0.00054 | 1 |
| 11657-12256 | 11956 | 0 | 0 | 0 |
| 11857-12456 | 12156 | 0 | 0 | 0 |
| 12057-12656 | 12356 | 0 | 0 | 0 |
| 12257-12858 | 12556 | 0.00141 | 0.00161 | 3 |
| 12457-13058 | 12756 | 0.00188 | 0.00215 | 4 |
| 12657-13258 | 12958 | 0.0044 | 0.0043 | 8 |
| 12859-13465 | 13158 | 0.00466 | 0.00483 | 9 |
| 13059-13669 | 13358 | 0.00603 | 0.00591 | 11 |
| 13259-13869 | 13569 | 0.00615 | 0.00591 | 11 |
| 13466-14069 | 13769 | 0.00564 | 0.00483 | 9 |
| 13670-14278 | 13969 | 0.00406 | 0.00376 | 7 |
| 13870-14484 | 14178 | 0.00496 | 0.00591 | 11 |
| 14070-14684 | 14378 | 0.0038 | 0.00483 | 9 |
| 14279-14886 | 14584 | 0.00581 | 0.00591 | 11 |
| 14485-15086 | 14784 | 0.00299 | 0.00269 | 5 |
| 14685-15295 | 14986 | 0.00457 | 0.0043 | 8 |
| 14887-15510 | 15195 | 0.00577 | 0.00591 | 11 |
| 15087-15718 | 15402 | 0.00731 | 0.00698 | 13 |
| 15296-16005 | 15612 | 0.00838 | 0.00752 | 14 |
| 15511-16205 | 15832 | 0.00718 | 0.00591 | 11 |
| 15719-16405 | 16105 | 0.00491 | 0.00376 | 7 |
| 16006-16605 | 16305 | 0.00316 | 0.00269 | 5 |
| 16206-16805 | 16505 | 0.00235 | 0.00322 | 6 |
| 16406-17007 | 16705 | 0.00513 | 0.00537 | 10 |
| 16606-17207 | 16907 | 0.00517 | 0.00537 | 10 |
| 16806-17407 | 17107 | 0.00466 | 0.0043 | 8 |
| 17008-17607 | 17307 | 0.00188 | 0.00215 | 4 |
| 17208-17833 | 17507 | 0.0041 | 0.00376 | 7 |
| 17408-18033 | 17709 | 0.00701 | 0.00591 | 11 |
| 17608-18233 | 17933 | 0.00765 | 0.00644 | 12 |
| 17834-18433 | 18133 | 0.00534 | 0.0043 | 8 |
| 18034-18633 | 18333 | 0.0015 | 0.00107 | 2 |
| 18234-18833 | 18533 | 0.00085 | 0.00054 | 1 |
| 18434-19033 | 18733 | 0.00359 | 0.00376 | 7 |
| 18634-19233 | 18933 | 0.00406 | 0.0043 | 8 |
| 18834-19433 | 19133 | 0.00479 | 0.00537 | 10 |
| 19034-19633 | 19333 | 0.00427 | 0.00376 | 7 |
| 19234-19833 | 19533 | 0.00598 | 0.00483 | 9 |
| 19434-20033 | 19733 | 0.00526 | 0.00376 | 7 |
| 19634-20233 | 19933 | 0.00312 | 0.00269 | 5 |
| 19834-20433 | 20133 | 0.00274 | 0.00269 | 5 |
| 20034-20633 | 20333 | 0.00436 | 0.00483 | 9 |
| 20234-20833 | 20533 | 0.00432 | 0.0043 | 8 |
| 20434-21033 | 20733 | 0.00316 | 0.00322 | 6 |
| 20634-21233 | 20933 | 0.00179 | 0.00161 | 3 |
| 20834-21433 | 21133 | 0.0009 | 0.00107 | 2 |
| 21034-21633 | 21333 | 0.00098 | 0.00161 | 3 |
| 21234-21833 | 21533 | 0.00145 | 0.00215 | 4 |
| 21434-22033 | 21733 | 0.00192 | 0.00269 | 5 |
| 21634-22233 | 21933 | 0.00415 | 0.0043 | 8 |
| 21834-22433 | 22133 | 0.00479 | 0.0043 | 8 |
| 22034-22633 | 22333 | 0.00543 | 0.00483 | 9 |
| 22234-22833 | 22533 | 0.00274 | 0.00269 | 5 |
| 22434-23033 | 22733 | 0.00209 | 0.00269 | 5 |
| 22634-23233 | 22933 | 0.00188 | 0.00215 | 4 |
| 22834-23433 | 23133 | 0.00188 | 0.00215 | 4 |
| 23034-23633 | 23333 | 0.00252 | 0.00269 | 5 |
| 23234-23833 | 23533 | 0.00162 | 0.00215 | 4 |
| 23434-24038 | 23733 | 0.00303 | 0.00376 | 7 |
| 23634-24238 | 23933 | 0.00444 | 0.00483 | 9 |
| 23834-24438 | 24138 | 0.00718 | 0.00752 | 14 |
| 24039-24638 | 24338 | 0.00577 | 0.00591 | 11 |
| 24239-24838 | 24538 | 0.00372 | 0.0043 | 8 |
| 24439-25038 | 24738 | 0.00124 | 0.00215 | 4 |
| 24639-25238 | 24938 | 0.00192 | 0.00269 | 5 |
| 24839-25438 | 25138 | 0.0012 | 0.00161 | 3 |
| 25039-25638 | 25338 | 0.00094 | 0.00107 | 2 |
| 25239-25838 | 25538 | 0 | 0 | 0 |
| 25439-26038 | 25738 | 0.00047 | 0.00054 | 1 |
| 25639-26238 | 25938 | 0.00073 | 0.00107 | 2 |
| 25839-26438 | 26138 | 0.00252 | 0.00215 | 4 |
| 26039-26638 | 26338 | 0.00295 | 0.00215 | 4 |
| 26239-26838 | 26538 | 0.00346 | 0.00322 | 6 |
| 26439-27038 | 26738 | 0.00218 | 0.00322 | 6 |
| 26639-27238 | 26938 | 0.00175 | 0.00322 | 6 |
| 26839-27438 | 27138 | 0.00124 | 0.00215 | 4 |
| 27039-27638 | 27338 | 0.0012 | 0.00161 | 3 |
| 27239-27838 | 27538 | 0.00073 | 0.00107 | 2 |
| 27439-28038 | 27738 | 0.00098 | 0.00161 | 3 |
| 27639-28238 | 27938 | 0.00051 | 0.00107 | 2 |
| 27839-28438 | 28138 | 0.00321 | 0.00269 | 5 |
| 28039-28638 | 28338 | 0.0056 | 0.00376 | 7 |
| 28239-28838 | 28538 | 0.01179 | 0.00913 | 17 |
| 28439-29113 | 28738 | 0.01231 | 0.01074 | 20 |
| 28639-29313 | 29013 | 0.01658 | 0.01558 | 29 |
| 28839-29514 | 29213 | 0.01124 | 0.01074 | 20 |
| 29114-29714 | 29413 | 0.01064 | 0.01074 | 20 |
| 29314-29924 | 29614 | 0.0053 | 0.00591 | 11 |
| 29515-30124 | 29814 | 0.00838 | 0.01074 | 20 |
| 29715-30332 | 30024 | 0.00735 | 0.00913 | 17 |
| 29925-30532 | 30232 | 0.00902 | 0.00913 | 17 |
| 30125-30732 | 30432 | 0.00607 | 0.00537 | 10 |
| 30333-30933 | 30632 | 0.0065 | 0.00537 | 10 |
| 30533-31148 | 30833 | 0.00603 | 0.00698 | 13 |
| 30733-31348 | 31040 | 0.01192 | 0.01128 | 21 |
| 30934-31555 | 31248 | 0.01265 | 0.01235 | 23 |
| 31149-31770 | 31455 | 0.01009 | 0.00913 | 17 |
| 31349-31976 | 31655 | 0.00594 | 0.00537 | 10 |
| 31556-32176 | 31870 | 0.00547 | 0.0043 | 8 |
| 31771-32376 | 32076 | 0.00641 | 0.00537 | 10 |
| 31977-32576 | 32276 | 0.0044 | 0.0043 | 8 |
| 32177-32781 | 32476 | 0.00261 | 0.00322 | 6 |
| 32377-33423 | 32676 | 0.00643 | 0.00537 | 10 |
| 32577-33623 | 33314 | 0.00759 | 0.00644 | 12 |
| 32782-33823 | 33523 | 0.00712 | 0.00591 | 11 |
| 33424-34025 | 33723 | 0.00252 | 0.00269 | 5 |
| 33624-34240 | 33925 | 0.00577 | 0.0043 | 8 |
| 33824-34440 | 34125 | 0.00603 | 0.00483 | 9 |
| 34026-34640 | 34340 | 0.00538 | 0.0043 | 8 |
| 34241-34840 | 34540 | 0.00111 | 0.00107 | 2 |
| 34441-35040 | 34740 | 0.00085 | 0.00054 | 1 |
| 34641-35240 | 34940 | 0.00085 | 0.00054 | 1 |
| 34841-35440 | 35140 | 0.00047 | 0.00054 | 1 |
| 35041-35640 | 35340 | 0.00047 | 0.00054 | 1 |
| 35241-35840 | 35540 | 0.00141 | 0.00161 | 3 |
| 35441-36040 | 35740 | 0.00094 | 0.00107 | 2 |
| 35641-36240 | 35940 | 0.0012 | 0.00161 | 3 |
| 35841-36440 | 36140 | 0.00137 | 0.00161 | 3 |
| 36041-36640 | 36340 | 0.00162 | 0.00215 | 4 |
| 36241-36854 | 36540 | 0.00226 | 0.00215 | 4 |
| 36441-37171 | 36740 | 0.00141 | 0.00161 | 3 |
| 36641-37375 | 37025 | 0.00141 | 0.00161 | 3 |
| 36855-37581 | 37271 | 0.00214 | 0.00269 | 5 |
| 37172-37781 | 37481 | 0.00235 | 0.00269 | 5 |
| 37376-37999 | 37681 | 0.00872 | 0.0102 | 19 |
| 37582-38204 | 37881 | 0.01051 | 0.01182 | 22 |
| 37782-38404 | 38100 | 0.01103 | 0.01289 | 24 |
| 38000-38604 | 38304 | 0.00624 | 0.00644 | 12 |
| 38205-38804 | 38504 | 0.0044 | 0.00483 | 9 |
| 38405-39004 | 38704 | 0.00342 | 0.00322 | 6 |
| 38605-39204 | 38904 | 0.00158 | 0.00161 | 3 |
| 38805-39404 | 39104 | 0 | 0 | 0 |
| 39005-39604 | 39304 | 0.00047 | 0.00054 | 1 |
| 39205-39804 | 39504 | 0.00094 | 0.00107 | 2 |
| 39405-40004 | 39704 | 0.00094 | 0.00107 | 2 |
| 39605-40204 | 39904 | 0.00047 | 0.00054 | 1 |
| 39805-40404 | 40104 | 0 | 0 | 0 |
| 40005-40604 | 40304 | 0.00085 | 0.00054 | 1 |
| 40205-40804 | 40504 | 0.00132 | 0.00107 | 2 |
| 40405-41004 | 40704 | 0.00269 | 0.00215 | 4 |
| 40605-41204 | 40904 | 0.00209 | 0.00215 | 4 |
| 40805-41404 | 41104 | 0.00162 | 0.00161 | 3 |
| 41005-41604 | 41304 | 0.00026 | 0.00054 | 1 |
| 41205-41804 | 41504 | 0.0009 | 0.00054 | 1 |
| 41405-42004 | 41704 | 0.00115 | 0.00107 | 2 |
| 41605-42204 | 41904 | 0.00115 | 0.00107 | 2 |
| 41805-42404 | 42104 | 0.00026 | 0.00054 | 1 |
| 42005-42604 | 42304 | 0.00026 | 0.00054 | 1 |
| 42205-42804 | 42504 | 0.00103 | 0.00215 | 4 |
| 42405-43004 | 42704 | 0.00192 | 0.00269 | 5 |
| 42605-43204 | 42904 | 0.00333 | 0.0043 | 8 |
| 42805-43404 | 43104 | 0.00256 | 0.00269 | 5 |
| 43005-43604 | 43304 | 0.00192 | 0.00269 | 5 |
| 43205-43804 | 43504 | 0.00167 | 0.00269 | 5 |
| 43405-44032 | 43704 | 0.00265 | 0.0043 | 8 |
| 43605-44247 | 43915 | 0.00517 | 0.00698 | 13 |
| 43805-44447 | 44132 | 0.00402 | 0.00537 | 10 |
| 44033-44647 | 44347 | 0.00329 | 0.0043 | 8 |
| 44248-44850 | 44547 | 0.00226 | 0.0043 | 8 |
| 44448-45050 | 44750 | 0.00353 | 0.00537 | 10 |
| 44648-45250 | 44950 | 0.00378 | 0.00591 | 11 |
| 44851-45450 | 45150 | 0.00229 | 0.00322 | 6 |
| 45051-45650 | 45350 | 0.00103 | 0.00215 | 4 |
| 45251-45855 | 45550 | 0.00235 | 0.00269 | 5 |
| 45451-46056 | 45750 | 0.00474 | 0.00644 | 12 |
| 45651-46256 | 45955 | 0.00534 | 0.00644 | 12 |
| 45856-46456 | 46156 | 0.00534 | 0.00698 | 13 |
| 46057-46736 | 46356 | 0.01047 | 0.00967 | 18 |
| 46257-46938 | 46556 | 0.01077 | 0.0102 | 19 |
| 46457-47138 | 46838 | 0.0112 | 0.0102 | 19 |
| 46737-47338 | 47038 | 0.00457 | 0.00483 | 9 |
| 46939-47538 | 47238 | 0.00368 | 0.0043 | 8 |
| 47139-47738 | 47438 | 0.00218 | 0.00376 | 7 |
| 47339-47938 | 47638 | 0.00103 | 0.00215 | 4 |
| 47539-48139 | 47838 | 0.00175 | 0.00322 | 6 |
| 47739-48339 | 48038 | 0.00346 | 0.00376 | 7 |
| 47939-48569 | 48239 | 0.00603 | 0.00537 | 10 |
| 48140-48823 | 48462 | 0.00748 | 0.00752 | 14 |
| 48340-49023 | 48669 | 0.00744 | 0.00806 | 15 |
| 48570-49239 | 48923 | 0.00615 | 0.00806 | 15 |
| 48824-49440 | 49139 | 0.00556 | 0.00644 | 12 |
| 49024-49640 | 49339 | 0.00312 | 0.00376 | 7 |
| 49240-49900 | 49540 | 0.00342 | 0.00376 | 7 |
| 49441-50100 | 49800 | 0.00158 | 0.00161 | 3 |
| 49641-50300 | 50000 | 0.00406 | 0.00376 | 7 |
| 49901-50500 | 50200 | 0.00333 | 0.00269 | 5 |
| 50101-50742 | 50400 | 0.01124 | 0.00913 | 17 |
| 50301-50942 | 50604 | 0.00966 | 0.00752 | 14 |
| 50501-51142 | 50842 | 0.00927 | 0.00752 | 14 |
| 50743-51342 | 51042 | 0.00209 | 0.00215 | 4 |
| 50943-51542 | 51242 | 0.00205 | 0.00215 | 4 |
| 51143-51742 | 51442 | 0.00338 | 0.00322 | 6 |
| 51343-51942 | 51642 | 0.00312 | 0.00269 | 5 |
| 51543-52142 | 51842 | 0.00316 | 0.00269 | 5 |
| 51743-52344 | 52042 | 0.00226 | 0.00215 | 4 |
| 51943-52544 | 52244 | 0.00205 | 0.00215 | 4 |
| 52143-52751 | 52444 | 0.00162 | 0.00215 | 4 |
| 52345-52998 | 52644 | 0.00476 | 0.00537 | 10 |
| 52545-53207 | 52874 | 0.00656 | 0.00644 | 12 |
| 52752-53584 | 53098 | 0.0081 | 0.00752 | 14 |
| 52999-53792 | 53471 | 0.00855 | 0.00913 | 17 |
| 53208-54001 | 53684 | 0.00769 | 0.00913 | 17 |
| 53585-54201 | 53901 | 0.00701 | 0.00859 | 16 |
| 53793-54402 | 54101 | 0.00509 | 0.00537 | 10 |
| 54002-54602 | 54301 | 0.00389 | 0.00376 | 7 |
| 54202-54802 | 54502 | 0.00303 | 0.00322 | 6 |
| 54403-55002 | 54702 | 0.00179 | 0.00161 | 3 |
| 54603-55202 | 54902 | 0.00179 | 0.00161 | 3 |
| 54803-55402 | 55102 | 0.00179 | 0.00161 | 3 |
| 55003-55602 | 55302 | 0.00047 | 0.00054 | 1 |
| 55203-55802 | 55502 | 0.0012 | 0.00161 | 3 |
| 55403-56002 | 55702 | 0.00209 | 0.00215 | 4 |
| 55603-56202 | 55902 | 0.00235 | 0.00269 | 5 |
| 55803-56402 | 56102 | 0.00162 | 0.00161 | 3 |
| 56003-56602 | 56302 | 0.00073 | 0.00107 | 2 |
| 56203-56802 | 56502 | 0.00184 | 0.00161 | 3 |
| 56403-57004 | 56702 | 0.00278 | 0.00269 | 5 |
| 56603-57221 | 56904 | 0.00376 | 0.0043 | 8 |
| 56803-57430 | 57104 | 0.0047 | 0.00537 | 10 |
| 57005-57630 | 57321 | 0.00402 | 0.00483 | 9 |
| 57222-57830 | 57530 | 0.00256 | 0.00269 | 5 |
| 57431-58030 | 57730 | 0.00444 | 0.00376 | 7 |
| 57631-58230 | 57930 | 0.00483 | 0.00376 | 7 |
| 57831-58430 | 58130 | 0.00752 | 0.00537 | 10 |
| 58031-58630 | 58330 | 0.00496 | 0.00376 | 7 |
| 58231-58830 | 58530 | 0.00521 | 0.00376 | 7 |
| 58431-59030 | 58730 | 0.00325 | 0.00322 | 6 |
| 58631-59256 | 58930 | 0.00389 | 0.00322 | 6 |
| 58831-59456 | 59155 | 0.0059 | 0.00537 | 10 |
| 59031-59666 | 59356 | 0.00842 | 0.00806 | 15 |
| 59257-59866 | 59566 | 0.00752 | 0.00806 | 15 |
| 59458-60066 | 59766 | 0.00513 | 0.00644 | 12 |
| 59667-60266 | 59966 | 0.00714 | 0.00644 | 12 |
| 59867-60481 | 60166 | 0.00718 | 0.00644 | 12 |
| 60067-60681 | 60366 | 0.00714 | 0.00591 | 11 |
| 60267-60881 | 60581 | 0.00214 | 0.00269 | 5 |
| 60482-61081 | 60781 | 0.0012 | 0.00161 | 3 |
| 60682-61281 | 60981 | 0.00171 | 0.00269 | 5 |
| 60882-61516 | 61181 | 0.00442 | 0.00537 | 10 |
| 61082-61721 | 61397 | 0.00442 | 0.00537 | 10 |
| 61282-61921 | 61616 | 0.00459 | 0.00483 | 9 |
| 61517-62121 | 61821 | 0.00415 | 0.00376 | 7 |
| 61722-62321 | 62021 | 0.00415 | 0.00376 | 7 |
| 61922-62521 | 62221 | 0.00385 | 0.00322 | 6 |
| 62122-62721 | 62421 | 0.00363 | 0.00322 | 6 |
| 62322-62983 | 62621 | 0.00654 | 0.00591 | 11 |
| 62522-63537 | 62821 | 0.00662 | 0.00644 | 12 |
| 62722-63737 | 63083 | 0.00799 | 0.00752 | 14 |
| 62984-63937 | 63637 | 0.00684 | 0.00591 | 11 |
| 63538-64137 | 63837 | 0.0059 | 0.00483 | 9 |
| 63738-64342 | 64037 | 0.00658 | 0.00591 | 11 |
| 63938-64565 | 64237 | 0.00838 | 0.00752 | 14 |
| 64138-64765 | 64465 | 0.01009 | 0.00859 | 16 |
| 64343-64965 | 64665 | 0.00662 | 0.00483 | 9 |
| 64566-65165 | 64865 | 0.00415 | 0.00269 | 5 |
| 64766-65365 | 65065 | 0.00423 | 0.00269 | 5 |
| 64966-65565 | 65265 | 0.00423 | 0.00322 | 6 |
| 65166-65772 | 65465 | 0.00538 | 0.0043 | 8 |
| 65366-65975 | 65665 | 0.00709 | 0.00698 | 13 |
| 65566-66195 | 65872 | 0.01056 | 0.00967 | 18 |
| 65773-66420 | 66095 | 0.00859 | 0.00859 | 16 |
| 65976-66620 | 66313 | 0.00581 | 0.00591 | 11 |
| 66196-66820 | 66520 | 0.00145 | 0.00215 | 4 |
| 66421-67020 | 66720 | 0.00167 | 0.00215 | 4 |
| 66621-67220 | 66920 | 0.00094 | 0.00107 | 2 |
| 66821-67420 | 67120 | 0.00094 | 0.00107 | 2 |
| 67021-67620 | 67320 | 0.00162 | 0.00215 | 4 |
| 67221-67822 | 67520 | 0.00731 | 0.00752 | 14 |
| 67421-68025 | 67721 | 0.01145 | 0.01182 | 22 |
| 67621-68264 | 67924 | 0.01124 | 0.01074 | 20 |
| 67823-68472 | 68155 | 0.00825 | 0.00806 | 15 |
| 68026-68672 | 68370 | 0.0041 | 0.00376 | 7 |
| 68265-68872 | 68572 | 0.00363 | 0.00376 | 7 |
| 68473-69072 | 68772 | 0.00209 | 0.00215 | 4 |
| 68673-69272 | 68972 | 0.00209 | 0.00215 | 4 |
| 68873-69494 | 69172 | 0.00462 | 0.00483 | 9 |
| 69073-69696 | 69376 | 0.00786 | 0.00752 | 14 |
| 69273-69896 | 69594 | 0.00927 | 0.00967 | 18 |
| 69495-70131 | 69796 | 0.00679 | 0.00752 | 14 |
| 69697-70341 | 70031 | 0.0044 | 0.00591 | 11 |
| 69897-70541 | 70241 | 0.0044 | 0.00537 | 10 |
| 70132-70741 | 70441 | 0.00342 | 0.00376 | 7 |
| 70342-70941 | 70641 | 0.00141 | 0.00161 | 3 |
| 70542-71142 | 70841 | 0.00226 | 0.00215 | 4 |
| 70742-71342 | 71041 | 0.00274 | 0.00269 | 5 |
| 70942-71542 | 71242 | 0.00513 | 0.00537 | 10 |
| 71143-71742 | 71442 | 0.00312 | 0.00376 | 7 |
| 71343-71953 | 71642 | 0.00534 | 0.00591 | 11 |
| 71543-72153 | 71842 | 0.00427 | 0.0043 | 8 |
| 71743-72353 | 72053 | 0.00491 | 0.0043 | 8 |
| 71954-72553 | 72253 | 0.00248 | 0.00215 | 4 |
| 72154-72760 | 72453 | 0.00346 | 0.00376 | 7 |
| 72354-72962 | 72660 | 0.00303 | 0.00376 | 7 |
| 72554-73163 | 72860 | 0.00329 | 0.0043 | 8 |
| 72761-73368 | 73063 | 0.00449 | 0.00537 | 10 |
| 72963-73571 | 73268 | 0.00491 | 0.00537 | 10 |
| 73164-73771 | 73471 | 0.00504 | 0.00483 | 9 |
| 73369-73971 | 73671 | 0.00252 | 0.00269 | 5 |
| 73572-74173 | 73871 | 0.00504 | 0.00483 | 9 |
| 73772-74373 | 74073 | 0.0044 | 0.0043 | 8 |
| 73972-74573 | 74273 | 0.00415 | 0.00376 | 7 |
| 74174-74782 | 74473 | 0.00073 | 0.00107 | 2 |
| 74374-74984 | 74673 | 0.00299 | 0.00322 | 6 |
| 74574-75185 | 74884 | 0.00316 | 0.00269 | 5 |
| 74783-75385 | 75084 | 0.00316 | 0.00269 | 5 |
| 74985-75585 | 75285 | 0.00137 | 0.00107 | 2 |
| 75186-75785 | 75485 | 0.00047 | 0.00054 | 1 |
| 75386-75985 | 75685 | 0.00047 | 0.00054 | 1 |
| 75586-76185 | 75885 | 0 | 0 | 0 |
| 75786-76385 | 76085 | 0 | 0 | 0 |
| 75986-76585 | 76285 | 0 | 0 | 0 |
| 76186-76815 | 76485 | 0.00124 | 0.00215 | 4 |
| 76386-77015 | 76685 | 0.00197 | 0.00322 | 6 |
| 76586-77215 | 76915 | 0.00385 | 0.00537 | 10 |
| 76816-77415 | 77115 | 0.00436 | 0.0043 | 8 |
| 77016-77615 | 77315 | 0.00453 | 0.00376 | 7 |
| 77216-77815 | 77515 | 0.00291 | 0.00215 | 4 |
| 77416-78024 | 77715 | 0.00517 | 0.0043 | 8 |
| 77616-78251 | 77915 | 0.00491 | 0.0043 | 8 |
| 77816-78465 | 78124 | 0.00594 | 0.00483 | 9 |
| 78025-78665 | 78365 | 0.00282 | 0.00215 | 4 |
| 78252-78865 | 78565 | 0.00303 | 0.00215 | 4 |
| 78466-79065 | 78765 | 0.00175 | 0.00107 | 2 |
| 78666-79272 | 78965 | 0.00402 | 0.00322 | 6 |
| 78866-79472 | 79165 | 0.00389 | 0.00376 | 7 |
| 79066-79672 | 79372 | 0.00795 | 0.00752 | 14 |
| 79273-79872 | 79572 | 0.00748 | 0.00644 | 12 |
| 79473-80102 | 79772 | 0.00838 | 0.00752 | 14 |
| 79673-80302 | 79984 | 0.00432 | 0.00376 | 7 |
| 79873-80502 | 80202 | 0.00162 | 0.00215 | 4 |
| 80103-80702 | 80402 | 0.00226 | 0.00215 | 4 |
| 80303-80923 | 80602 | 0.01299 | 0.01182 | 22 |
| 80503-81123 | 80823 | 0.01436 | 0.01289 | 24 |
| 80703-81323 | 81023 | 0.01256 | 0.01128 | 21 |
| 80924-81523 | 81223 | 0.00184 | 0.00161 | 3 |
| 81124-81723 | 81423 | 0.00184 | 0.00161 | 3 |
| 81324-81923 | 81623 | 0.00209 | 0.00215 | 4 |
| 81524-82123 | 81823 | 0.00376 | 0.0043 | 8 |
| 81724-82323 | 82023 | 0.00286 | 0.00376 | 7 |
| 81924-82523 | 82223 | 0.00239 | 0.00322 | 6 |
| 82124-82723 | 82423 | 0.00303 | 0.00376 | 7 |
| 82324-82923 | 82623 | 0.00346 | 0.0043 | 8 |
| 82524-83124 | 82823 | 0.0041 | 0.00483 | 9 |
| 82724-83324 | 83023 | 0.00274 | 0.00322 | 6 |
| 82924-83524 | 83224 | 0.00303 | 0.00376 | 7 |
| 83125-83725 | 83424 | 0.00265 | 0.00376 | 7 |
| 83325-83925 | 83624 | 0.00218 | 0.00322 | 6 |
| 83525-84125 | 83825 | 0.00171 | 0.00269 | 5 |
| 83726-84325 | 84025 | 0.00419 | 0.00483 | 9 |
| 83926-84525 | 84225 | 0.00372 | 0.0043 | 8 |
| 84126-84725 | 84425 | 0.00521 | 0.00483 | 9 |
| 84326-84934 | 84625 | 0.0062 | 0.00591 | 11 |
| 84526-85134 | 84834 | 0.00709 | 0.00698 | 13 |
| 84726-85335 | 85034 | 0.00607 | 0.00698 | 13 |
| 84935-85551 | 85235 | 0.00637 | 0.00644 | 12 |
| 85135-85754 | 85435 | 0.00821 | 0.00806 | 15 |
| 85336-85954 | 85654 | 0.00748 | 0.00698 | 13 |
| 85552-86154 | 85854 | 0.00368 | 0.00376 | 7 |
| 85755-86354 | 86054 | 0.00226 | 0.00215 | 4 |
| 85955-86554 | 86254 | 0.00474 | 0.0043 | 8 |
| 86155-86754 | 86454 | 0.00585 | 0.00537 | 10 |
| 86355-87017 | 86654 | 0.00684 | 0.00644 | 12 |
| 86555-87217 | 86854 | 0.00436 | 0.0043 | 8 |
| 86755-87420 | 87117 | 0.00393 | 0.00376 | 7 |
| 87018-87620 | 87318 | 0.00162 | 0.00161 | 3 |
| 87218-87820 | 87520 | 0.00115 | 0.00107 | 2 |
| 87421-88020 | 87720 | 0.00064 | 0.00054 | 1 |
| 87621-88220 | 87920 | 0.00064 | 0.00054 | 1 |
| 87821-88420 | 88120 | 0.00064 | 0.00054 | 1 |
| 88021-88620 | 88320 | 0 | 0 | 0 |
| 88221-88820 | 88520 | 0 | 0 | 0 |
| 88421-89020 | 88720 | 0 | 0 | 0 |
| 88621-89220 | 88920 | 0.00188 | 0.00161 | 3 |
| 88821-89420 | 89120 | 0.00188 | 0.00161 | 3 |
| 89021-89620 | 89320 | 0.00235 | 0.00215 | 4 |
| 89221-89820 | 89520 | 0.00162 | 0.00161 | 3 |
| 89421-90020 | 89720 | 0.00162 | 0.00161 | 3 |
| 89621-90220 | 89920 | 0.00115 | 0.00107 | 2 |
| 89821-90420 | 90120 | 0.00026 | 0.00054 | 1 |
| 90021-90620 | 90320 | 0.00026 | 0.00054 | 1 |
| 90221-90820 | 90520 | 0.00026 | 0.00054 | 1 |
| 90421-91020 | 90720 | 0.00158 | 0.00161 | 3 |
| 90621-91220 | 90920 | 0.00158 | 0.00161 | 3 |
| 90821-91420 | 91120 | 0.00158 | 0.00161 | 3 |
| 91021-91620 | 91320 | 0 | 0 | 0 |
| 91221-91820 | 91520 | 0 | 0 | 0 |
| 91421-92020 | 91720 | 0.00085 | 0.00054 | 1 |
| 91621-92220 | 91920 | 0.00132 | 0.00107 | 2 |
| 91821-92420 | 92120 | 0.00158 | 0.00161 | 3 |
| 92021-92620 | 92320 | 0.00073 | 0.00107 | 2 |
| 92221-92835 | 92520 | 0.00103 | 0.00215 | 4 |
| 92421-93035 | 92735 | 0.00124 | 0.00215 | 4 |
| 92621-93235 | 92935 | 0.00274 | 0.00322 | 6 |
| 92836-93435 | 93135 | 0.00244 | 0.00215 | 4 |
| 93036-93635 | 93335 | 0.00197 | 0.00161 | 3 |
| 93236-93835 | 93535 | 0.00047 | 0.00054 | 1 |
| 93436-94065 | 93735 | 0 | 0 | 0 |
| 93636-94265 | 93935 | 0.0009 | 0.00054 | 1 |
| 93836-94465 | 94165 | 0.00192 | 0.00161 | 3 |
| 94066-94665 | 94365 | 0.00192 | 0.00161 | 3 |
| 94266-94865 | 94565 | 0.00103 | 0.00107 | 2 |
| 94466-95065 | 94765 | 0 | 0 | 0 |
| 94666-95265 | 94965 | 0 | 0 | 0 |
| 94866-95486 | 95165 | 0 | 0 | 0 |
| 95066-95686 | 95386 | 0 | 0 | 0 |
| 95266-95886 | 95586 | 0.00047 | 0.00054 | 1 |
| 95487-96086 | 95786 | 0.00047 | 0.00054 | 1 |
| 95687-96286 | 95986 | 0.00047 | 0.00054 | 1 |
| 95887-96486 | 96186 | 0.00342 | 0.00215 | 4 |
| 96087-96686 | 96386 | 0.00342 | 0.00215 | 4 |
| 96287-96886 | 96586 | 0.00342 | 0.00215 | 4 |
| 96487-97086 | 96786 | 0 | 0 | 0 |
| 96687-97286 | 96986 | 0 | 0 | 0 |
| 96887-97486 | 97186 | 0.00047 | 0.00054 | 1 |
| 97087-97686 | 97386 | 0.00094 | 0.00107 | 2 |
| 97287-97886 | 97586 | 0.00094 | 0.00107 | 2 |
| 97487-98086 | 97786 | 0.00047 | 0.00054 | 1 |
| 97687-98286 | 97986 | 0 | 0 | 0 |
| 97887-98486 | 98186 | 0 | 0 | 0 |
| 98087-98686 | 98386 | 0.00064 | 0.00054 | 1 |
| 98287-98886 | 98586 | 0.00064 | 0.00054 | 1 |
| 98487-99086 | 98786 | 0.00064 | 0.00054 | 1 |
| 98687-99286 | 98986 | 0.00026 | 0.00054 | 1 |
| 98887-99486 | 99186 | 0.00026 | 0.00054 | 1 |
| 99087-99686 | 99386 | 0.00128 | 0.00269 | 5 |
| 99287-99886 | 99586 | 0.00103 | 0.00215 | 4 |
| 99487-100086 | 99786 | 0.00103 | 0.00215 | 4 |
| 99687-100286 | 99986 | 0.00026 | 0.00054 | 1 |
| 99887-100486 | 100186 | 0.00026 | 0.00054 | 1 |
| 100087-100686 | 100386 | 0.00026 | 0.00054 | 1 |
| 100287-100886 | 100586 | 0 | 0 | 0 |
| 100487-101086 | 100786 | 0 | 0 | 0 |
| 100687-101286 | 100986 | 0 | 0 | 0 |
| 100887-101496 | 101186 | 0.0009 | 0.00054 | 1 |
| 101087-101696 | 101386 | 0.00137 | 0.00107 | 2 |
| 101287-101896 | 101596 | 0.00137 | 0.00107 | 2 |
| 101497-102096 | 101796 | 0.00141 | 0.00161 | 3 |
| 101697-102296 | 101996 | 0.00094 | 0.00107 | 2 |
| 101897-102497 | 102196 | 0.00094 | 0.00107 | 2 |
| 102097-102792 | 102397 | 0 | 0 | 0 |
| 102297-102992 | 102692 | 0.00064 | 0.00054 | 1 |
| 102498-103192 | 102892 | 0.00128 | 0.00107 | 2 |
| 102793-103392 | 103092 | 0.00128 | 0.00107 | 2 |
| 102993-103592 | 103292 | 0.00064 | 0.00054 | 1 |
| 103193-103792 | 103492 | 0 | 0 | 0 |
| 103393-103992 | 103692 | 0 | 0 | 0 |
| 103593-104192 | 103892 | 0 | 0 | 0 |
| 103793-104392 | 104092 | 0 | 0 | 0 |
| 103993-104592 | 104292 | 0.0009 | 0.00054 | 1 |
| 104193-104792 | 104492 | 0.0009 | 0.00054 | 1 |
| 104393-104992 | 104692 | 0.00179 | 0.00107 | 2 |
| 104593-105192 | 104892 | 0.0009 | 0.00054 | 1 |
| 104793-105392 | 105092 | 0.0009 | 0.00054 | 1 |
| 104993-105593 | 105292 | 0 | 0 | 0 |
| 105193-105793 | 105493 | 0 | 0 | 0 |
| 105393-105993 | 105693 | 0 | 0 | 0 |
| 105594-106193 | 105893 | 0 | 0 | 0 |
| 105794-106393 | 106093 | 0 | 0 | 0 |
| 105994-106593 | 106293 | 0 | 0 | 0 |
| 106194-106796 | 106493 | 0 | 0 | 0 |
| 106394-106996 | 106696 | 0.00085 | 0.00054 | 1 |
| 106594-107196 | 106896 | 0.00085 | 0.00054 | 1 |
| 106797-107396 | 107096 | 0.00085 | 0.00054 | 1 |
| 106997-107596 | 107296 | 0 | 0 | 0 |
| 107197-107796 | 107496 | 0 | 0 | 0 |
| 107397-107996 | 107696 | 0 | 0 | 0 |
| 107597-108196 | 107896 | 0 | 0 | 0 |
| 107797-108396 | 108096 | 0 | 0 | 0 |
| 107997-108596 | 108296 | 0 | 0 | 0 |
| 108197-108796 | 108496 | 0 | 0 | 0 |
| 108397-108996 | 108696 | 0 | 0 | 0 |
| 108597-109196 | 108896 | 0 | 0 | 0 |
| 108797-109396 | 109096 | 0 | 0 | 0 |
| 108997-109596 | 109296 | 0 | 0 | 0 |
| 109197-109796 | 109496 | 0 | 0 | 0 |
| 109397-109996 | 109696 | 0 | 0 | 0 |
| 109597-110196 | 109896 | 0 | 0 | 0 |
| 109797-110396 | 110096 | 0 | 0 | 0 |
| 109997-110596 | 110296 | 0 | 0 | 0 |
| 110197-110796 | 110496 | 0 | 0 | 0 |
| 110397-110996 | 110696 | 0.00274 | 0.00269 | 5 |
| 110597-111196 | 110896 | 0.00274 | 0.00269 | 5 |
| 110797-111396 | 111096 | 0.00397 | 0.00376 | 7 |
| 110997-111596 | 111296 | 0.00124 | 0.00107 | 2 |
| 111197-111796 | 111496 | 0.00124 | 0.00107 | 2 |
| 111397-111996 | 111696 | 0 | 0 | 0 |
| 111597-112196 | 111896 | 0.00085 | 0.00054 | 1 |
| 111797-112396 | 112096 | 0.00321 | 0.00322 | 6 |
| 111997-112596 | 112296 | 0.00368 | 0.00376 | 7 |
| 112197-112805 | 112496 | 0.00419 | 0.00483 | 9 |
| 112397-113005 | 112705 | 0.00248 | 0.00269 | 5 |
| 112597-113205 | 112905 | 0.00338 | 0.00322 | 6 |
| 112806-113405 | 113105 | 0.00201 | 0.00161 | 3 |
| 113006-113625 | 113305 | 0.00491 | 0.0043 | 8 |
| 113206-113825 | 113505 | 0.00547 | 0.00591 | 11 |
| 113406-114025 | 113725 | 0.00658 | 0.00698 | 13 |
| 113626-114225 | 113925 | 0.00436 | 0.00483 | 9 |
| 113826-114425 | 114125 | 0.0056 | 0.00483 | 9 |
| 114026-114625 | 114325 | 0.00645 | 0.00537 | 10 |
| 114226-114825 | 114525 | 0.0056 | 0.00483 | 9 |
| 114426-115025 | 114725 | 0.00333 | 0.00322 | 6 |
| 114626-115225 | 114925 | 0.00269 | 0.00269 | 5 |
| 114826-115425 | 115125 | 0.00308 | 0.00269 | 5 |
| 115026-115625 | 115325 | 0.00312 | 0.00269 | 5 |
| 115226-115825 | 115525 | 0.00179 | 0.00161 | 3 |
| 115426-116025 | 115725 | 0.00325 | 0.00376 | 7 |
| 115626-116239 | 115925 | 0.00393 | 0.0043 | 8 |
| 115826-116442 | 116134 | 0.00645 | 0.00698 | 13 |
| 116035-116642 | 116342 | 0.00462 | 0.00483 | 9 |
| 116240-116848 | 116542 | 0.00504 | 0.00537 | 10 |
| 116443-117069 | 116746 | 0.00483 | 0.00537 | 10 |
| 116643-117274 | 116956 | 0.0053 | 0.00591 | 11 |
| 116849-117484 | 117174 | 0.00697 | 0.00859 | 16 |
| 117070-117684 | 117375 | 0.00697 | 0.00806 | 15 |
| 117275-117884 | 117584 | 0.0065 | 0.00752 | 14 |
| 117485-118084 | 117784 | 0.0035 | 0.00376 | 7 |
| 117685-118284 | 117984 | 0.00184 | 0.00215 | 4 |
| 117885-118484 | 118184 | 0.00308 | 0.00322 | 6 |
| 118085-118684 | 118384 | 0.00321 | 0.00269 | 5 |
| 118285-118884 | 118584 | 0.00423 | 0.0043 | 8 |
| 118485-119084 | 118784 | 0.00825 | 0.00859 | 16 |
| 118685-119284 | 118984 | 0.00876 | 0.00967 | 18 |
| 118885-119484 | 119184 | 0.00756 | 0.00806 | 15 |
| 119085-119684 | 119384 | 0.00209 | 0.00269 | 5 |
| 119285-119884 | 119584 | 0.00145 | 0.00215 | 4 |
| 119485-120084 | 119784 | 0.00278 | 0.00322 | 6 |
| 119685-120284 | 119984 | 0.00252 | 0.00269 | 5 |
| 119885-120484 | 120184 | 0.0041 | 0.00376 | 7 |
| 120085-120684 | 120384 | 0.00278 | 0.00269 | 5 |
| 120285-120884 | 120584 | 0.00278 | 0.00269 | 5 |
| 120485-121084 | 120784 | 0.00577 | 0.0043 | 8 |
| 120685-121284 | 120984 | 0.00603 | 0.00483 | 9 |
| 120885-121484 | 121184 | 0.00675 | 0.00591 | 11 |
| 121085-121684 | 121384 | 0.0044 | 0.00537 | 10 |
| 121285-121884 | 121584 | 0.00368 | 0.0043 | 8 |
| 121485-122084 | 121784 | 0.00321 | 0.00376 | 7 |
| 121685-122300 | 121984 | 0.00363 | 0.00376 | 7 |
| 121885-122522 | 122186 | 0.00556 | 0.00644 | 12 |
| 122085-122722 | 122406 | 0.00556 | 0.00644 | 12 |
| 122301-122922 | 122622 | 0.00218 | 0.00322 | 6 |
| 122523-123122 | 122822 | 0.00205 | 0.00161 | 3 |
| 122723-123322 | 123022 | 0.00269 | 0.00161 | 3 |
| 122923-123522 | 123222 | 0.00517 | 0.00376 | 7 |
| 123123-123722 | 123422 | 0.00453 | 0.00376 | 7 |
| 123323-123923 | 123622 | 0.00662 | 0.00537 | 10 |
| 123523-124146 | 123823 | 0.00624 | 0.00591 | 11 |
| 123723-124346 | 124041 | 0.0062 | 0.00591 | 11 |
| 123924-124550 | 124246 | 0.00709 | 0.00752 | 14 |
| 124147-124752 | 124450 | 0.00526 | 0.00537 | 10 |
| 124347-124952 | 124650 | 0.00415 | 0.0043 | 8 |
| 124551-125152 | 124852 | 0.00073 | 0.00107 | 2 |
| 124753-125352 | 125052 | 0.00111 | 0.00107 | 2 |
| 124953-125552 | 125252 | 0.00231 | 0.00269 | 5 |
| 125153-125752 | 125452 | 0.00282 | 0.00376 | 7 |
| 125353-125952 | 125652 | 0.00282 | 0.00376 | 7 |
| 125553-126152 | 125852 | 0.00209 | 0.00269 | 5 |
| 125753-126352 | 126052 | 0.00222 | 0.00215 | 4 |
| 125953-126553 | 126252 | 0.00158 | 0.00161 | 3 |
| 126153-126753 | 126452 | 0.00479 | 0.0043 | 8 |
| 126353-126959 | 126653 | 0.00504 | 0.0043 | 8 |
| 126554-127159 | 126853 | 0.01128 | 0.01074 | 20 |
| 126754-127378 | 127059 | 0.0097 | 0.00967 | 18 |
| 126960-127599 | 127263 | 0.0115 | 0.01128 | 21 |
| 127160-127799 | 127478 | 0.00573 | 0.00537 | 10 |
| 127379-127999 | 127699 | 0.00812 | 0.00752 | 14 |
| 127600-128199 | 127899 | 0.00611 | 0.00591 | 11 |
| 127800-128399 | 128099 | 0.00701 | 0.00698 | 13 |
| 128000-128599 | 128299 | 0.0056 | 0.00537 | 10 |
| 128200-128799 | 128499 | 0.00962 | 0.00859 | 16 |
| 128400-128999 | 128699 | 0.01077 | 0.00967 | 18 |
| 128600-129205 | 128899 | 0.01235 | 0.01074 | 20 |
| 128800-129405 | 129099 | 0.01308 | 0.01182 | 22 |
| 129000-129605 | 129305 | 0.01316 | 0.01235 | 23 |
| 129206-129856 | 129505 | 0.01415 | 0.01396 | 26 |
| 129406-130056 | 129756 | 0.01389 | 0.01343 | 25 |
| 129606-130256 | 129956 | 0.01299 | 0.01182 | 22 |
| 129857-130483 | 130156 | 0.01333 | 0.01289 | 24 |
| 130057-130683 | 130383 | 0.01024 | 0.00967 | 18 |
| 130257-130883 | 130583 | 0.01024 | 0.01074 | 20 |
| 130484-131083 | 130783 | 0.00733 | 0.00698 | 13 |
| 130684-131283 | 130983 | 0.00526 | 0.00591 | 11 |
| 130884-131483 | 131183 | 0.00444 | 0.00376 | 7 |
| 131084-131683 | 131383 | 0.00201 | 0.00161 | 3 |
| 131284-131892 | 131583 | 0.00291 | 0.00269 | 5 |
| 131484-132092 | 131783 | 0.00483 | 0.00537 | 10 |
| 131684-132292 | 131992 | 0.00419 | 0.00483 | 9 |
| 131893-132492 | 132192 | 0.00368 | 0.00376 | 7 |
| 132093-132692 | 132392 | 0.00085 | 0.00054 | 1 |
| 132293-132892 | 132592 | 0.00085 | 0.00054 | 1 |
| 132493-133092 | 132792 | 0.00077 | 0.00054 | 1 |
| 132693-133292 | 132992 | 0.00124 | 0.00107 | 2 |
| 132893-133492 | 133192 | 0.00171 | 0.00161 | 3 |
| 133093-133692 | 133392 | 0.00321 | 0.00322 | 6 |
| 133293-133892 | 133592 | 0.00274 | 0.00269 | 5 |
| 133493-134092 | 133792 | 0.00226 | 0.00215 | 4 |
| 133693-134292 | 133992 | 0 | 0 | 0 |
| 133893-134492 | 134192 | 0 | 0 | 0 |
| 134093-134692 | 134392 | 0 | 0 | 0 |
| 134293-134892 | 134592 | 0 | 0 | 0 |
| 134493-135092 | 134792 | 0 | 0 | 0 |
| 134693-135292 | 134992 | 0 | 0 | 0 |
| 134893-135492 | 135192 | 0 | 0 | 0 |
| 135093-135692 | 135392 | 0 | 0 | 0 |
| 135293-135892 | 135592 | 0 | 0 | 0 |
| 135493-136092 | 135792 | 0 | 0 | 0 |
| 135693-136292 | 135992 | 0 | 0 | 0 |
| 135893-136492 | 136192 | 0 | 0 | 0 |
| 136093-136692 | 136392 | 0 | 0 | 0 |
| 136293-136892 | 136592 | 0 | 0 | 0 |
| 136493-137092 | 136792 | 0 | 0 | 0 |
| 136693-137292 | 136992 | 0 | 0 | 0 |
| 136893-137492 | 137192 | 0 | 0 | 0 |
| 137093-137692 | 137392 | 0.00085 | 0.00054 | 1 |
| 137293-137895 | 137592 | 0.00085 | 0.00054 | 1 |
| 137493-138095 | 137795 | 0.00085 | 0.00054 | 1 |
| 137693-138295 | 137995 | 0 | 0 | 0 |
| 137896-138495 | 138195 | 0 | 0 | 0 |
| 138096-138695 | 138395 | 0 | 0 | 0 |
| 138296-138895 | 138595 | 0 | 0 | 0 |
| 138496-139096 | 138795 | 0 | 0 | 0 |
| 138696-139296 | 138995 | 0 | 0 | 0 |
| 138896-139496 | 139196 | 0 | 0 | 0 |
| 139097-139696 | 139396 | 0.0009 | 0.00054 | 1 |
| 139297-139896 | 139596 | 0.0009 | 0.00054 | 1 |
| 139497-140096 | 139796 | 0.00179 | 0.00107 | 2 |
| 139697-140296 | 139996 | 0.0009 | 0.00054 | 1 |
| 139897-140496 | 140196 | 0.0009 | 0.00054 | 1 |
| 140097-140696 | 140396 | 0 | 0 | 0 |
| 140297-140896 | 140596 | 0 | 0 | 0 |
| 140497-141096 | 140796 | 0 | 0 | 0 |
| 140697-141296 | 140996 | 0 | 0 | 0 |
| 140897-141496 | 141196 | 0.00128 | 0.00107 | 2 |
| 141097-141696 | 141396 | 0.00128 | 0.00107 | 2 |
| 141297-141991 | 141596 | 0.00128 | 0.00107 | 2 |
| 141497-142192 | 141796 | 0 | 0 | 0 |
| 141697-142392 | 142092 | 0 | 0 | 0 |
| 141992-142592 | 142292 | 0.00094 | 0.00107 | 2 |
| 142193-142792 | 142492 | 0.00094 | 0.00107 | 2 |
| 142393-143002 | 142692 | 0.00231 | 0.00215 | 4 |
| 142593-143202 | 142892 | 0.00137 | 0.00107 | 2 |
| 142793-143402 | 143102 | 0.00137 | 0.00107 | 2 |
| 143003-143602 | 143302 | 0 | 0 | 0 |
| 143203-143802 | 143502 | 0 | 0 | 0 |
| 143403-144002 | 143702 | 0 | 0 | 0 |
| 143603-144202 | 143902 | 0 | 0 | 0 |
| 143803-144402 | 144102 | 0.00026 | 0.00054 | 1 |
| 144003-144602 | 144302 | 0.00026 | 0.00054 | 1 |
| 144203-144802 | 144502 | 0.00026 | 0.00054 | 1 |
| 144403-145002 | 144702 | 0.00103 | 0.00215 | 4 |
| 144603-145202 | 144902 | 0.00128 | 0.00269 | 5 |
| 144803-145402 | 145102 | 0.00128 | 0.00269 | 5 |
| 145003-145602 | 145302 | 0.00026 | 0.00054 | 1 |
| 145203-145802 | 145502 | 0 | 0 | 0 |
| 145403-146002 | 145702 | 0.00064 | 0.00054 | 1 |
| 145603-146202 | 145902 | 0.00064 | 0.00054 | 1 |
| 145803-146402 | 146102 | 0.00064 | 0.00054 | 1 |
| 146003-146602 | 146302 | 0 | 0 | 0 |
| 146203-146802 | 146502 | 0 | 0 | 0 |
| 146403-147002 | 146702 | 0.00094 | 0.00107 | 2 |
| 146603-147202 | 146902 | 0.00094 | 0.00107 | 2 |
| 146803-147402 | 147102 | 0.00094 | 0.00107 | 2 |
| 147003-147602 | 147302 | 0 | 0 | 0 |
| 147203-147802 | 147502 | 0 | 0 | 0 |
| 147403-148002 | 147702 | 0.00342 | 0.00215 | 4 |
| 147603-148202 | 147902 | 0.00342 | 0.00215 | 4 |
| 147803-148402 | 148102 | 0.00342 | 0.00215 | 4 |
| 148003-148602 | 148302 | 0 | 0 | 0 |
| 148203-148802 | 148502 | 0.00047 | 0.00054 | 1 |
| 148403-149002 | 148702 | 0.00047 | 0.00054 | 1 |
| 148603-149223 | 148902 | 0.00047 | 0.00054 | 1 |
| 148803-149423 | 149102 | 0 | 0 | 0 |
| 149003-149623 | 149323 | 0 | 0 | 0 |
| 149224-149823 | 149523 | 0 | 0 | 0 |
| 149424-150023 | 149723 | 0 | 0 | 0 |
| 149624-150223 | 149923 | 0.00103 | 0.00107 | 2 |
| 149824-150423 | 150123 | 0.00192 | 0.00161 | 3 |
| 150024-150653 | 150323 | 0.00192 | 0.00161 | 3 |
| 150224-150853 | 150553 | 0.0009 | 0.00054 | 1 |
| 150424-151053 | 150753 | 0 | 0 | 0 |
| 150654-151253 | 150953 | 0.00047 | 0.00054 | 1 |
| 150854-151453 | 151153 | 0.00244 | 0.00215 | 4 |
| 151054-151653 | 151353 | 0.00244 | 0.00215 | 4 |
| 151254-151868 | 151553 | 0.00248 | 0.00269 | 5 |
| 151454-152068 | 151753 | 0.00077 | 0.00161 | 3 |
| 151654-152268 | 151968 | 0.00077 | 0.00161 | 3 |
| 151869-152468 | 152168 | 0.00073 | 0.00107 | 2 |
| 152069-152668 | 152368 | 0.00132 | 0.00107 | 2 |
| 152269-152868 | 152568 | 0.00132 | 0.00107 | 2 |
| 152469-153068 | 152768 | 0.00085 | 0.00054 | 1 |
| 152669-153268 | 152968 | 0 | 0 | 0 |
| 152869-153468 | 153168 | 0 | 0 | 0 |
| 153069-153668 | 153368 | 0.00158 | 0.00161 | 3 |
| 153269-153868 | 153568 | 0.00158 | 0.00161 | 3 |
| 153469-154068 | 153768 | 0.00184 | 0.00215 | 4 |
| 153669-154268 | 153968 | 0.00026 | 0.00054 | 1 |
| 153869-154468 | 154168 | 0.00026 | 0.00054 | 1 |
| 154069-154668 | 154368 | 0.00115 | 0.00107 | 2 |
| 154269-154868 | 154568 | 0.00162 | 0.00161 | 3 |
| 154469-155068 | 154768 | 0.00162 | 0.00161 | 3 |
| 154669-155268 | 154968 | 0.00047 | 0.00054 | 1 |
| 154869-155468 | 155168 | 0.00188 | 0.00161 | 3 |
| 155069-155668 | 155368 | 0.00188 | 0.00161 | 3 |
| 155269-155868 | 155568 | 0.00188 | 0.00161 | 3 |
| 155469-156068 | 155768 | 0 | 0 | 0 |
| 155669-156268 | 155968 | 0 | 0 | 0 |
| 155869-156468 | 156168 | 0.00064 | 0.00054 | 1 |
| 156069-156668 | 156368 | 0.00064 | 0.00054 | 1 |
| 156269-156868 | 156568 | 0.00064 | 0.00054 | 1 |
| 156469-157068 | 156768 | 0 | 0 | 0 |
| 156669-157193 | 156888 | 0.00158 | 0.00147 | 2 |
