# Supplementary material for: Complete chloroplast of four Sanicula taxa (Apiaceae) endemic to China: lights into genome structure, comparative analysis, and phylogenetic relationships
Source: BMC Plant Biol. 2023 Sep 21;23:444. doi: 10.1186/s12870-023-04447-w (PMC10512634; doi:10.1186/s12870-023-04447-w)
Supplement: Supplementary file 2 — Additional file 2: Table S2. The comparison of SSRs among four newly sequenced Sanicula taxa chloroplast genomes. [file 12870_2023_4447_MOESM2_ESM.docx]

Table S2. The comparison of SSRs among four newly sequenced *Sanicula* taxa chloroplast genomes.

| **ID** | **Species name** | **SSR nr.** | **SSR type** | **SSR** | **size** | **start** | **end** |
| --- | --- | --- | --- | --- | --- | --- | --- |
| LHM1005 | *Sanicula_caerulescens* | 1 | p1 | (A)10 | 10 | 3915 | 3924 |
| LHM1005 | *Sanicula_caerulescens* | 2 | p1 | (A)10 | 10 | 4512 | 4521 |
| LHM1005 | *Sanicula_caerulescens* | 3 | p2 | (AT)9 | 18 | 10591 | 10608 |
| LHM1005 | *Sanicula_caerulescens* | 4 | p1 | (A)10 | 10 | 12502 | 12511 |
| LHM1005 | *Sanicula_caerulescens* | 5 | p1 | (T)11 | 11 | 13212 | 13222 |
| LHM1005 | *Sanicula_caerulescens* | 6 | p1 | (A)11 | 11 | 13842 | 13852 |
| LHM1005 | *Sanicula_caerulescens* | 7 | p1 | (T)11 | 11 | 16515 | 16525 |
| LHM1005 | *Sanicula_caerulescens* | 8 | p1 | (A)10 | 10 | 17356 | 17365 |
| LHM1005 | *Sanicula_caerulescens* | 9 | p1 | (T)11 | 11 | 19588 | 19598 |
| LHM1005 | *Sanicula_caerulescens* | 10 | p1 | (T)12 | 12 | 27322 | 27333 |
| LHM1005 | *Sanicula_caerulescens* | 11 | p1 | (A)13 | 13 | 32489 | 32501 |
| LHM1005 | *Sanicula_caerulescens* | 12 | p2 | (AT)6 | 12 | 33754 | 33765 |
| LHM1005 | *Sanicula_caerulescens* | 13 | p1 | (T)10 | 10 | 45550 | 45559 |
| LHM1005 | *Sanicula_caerulescens* | 14 | p2 | (TA)7 | 14 | 48191 | 48204 |
| LHM1005 | *Sanicula_caerulescens* | 15 | p1 | (T)10 | 10 | 56057 | 56066 |
| LHM1005 | *Sanicula_caerulescens* | 16 | p2 | (TA)8 | 16 | 63569 | 63584 |
| LHM1005 | *Sanicula_caerulescens* | 17 | p1 | (A)11 | 11 | 65136 | 65146 |
| LHM1005 | *Sanicula_caerulescens* | 18 | p2 | (TA)7 | 14 | 65248 | 65261 |
| LHM1005 | *Sanicula_caerulescens* | 19 | p1 | (T)10 | 10 | 67199 | 67208 |
| LHM1005 | *Sanicula_caerulescens* | 20 | p1 | (A)10 | 10 | 67572 | 67581 |
| LHM1005 | *Sanicula_caerulescens* | 21 | p2 | (TA)7 | 14 | 68493 | 68506 |
| LHM1005 | *Sanicula_caerulescens* | 22 | p1 | (A)11 | 11 | 72060 | 72070 |
| LHM1005 | *Sanicula_caerulescens* | 23 | p1 | (T)13 | 13 | 72330 | 72342 |
| LHM1005 | *Sanicula_caerulescens* | 24 | p1 | (A)11 | 11 | 72496 | 72506 |
| LHM1005 | *Sanicula_caerulescens* | 25 | p1 | (A)11 | 11 | 73137 | 73147 |
| LHM1005 | *Sanicula_caerulescens* | 26 | p1 | (T)10 | 10 | 80148 | 80157 |
| LHM1005 | *Sanicula_caerulescens* | 27 | p1 | (T)12 | 12 | 82080 | 82091 |
| LHM1005 | *Sanicula_caerulescens* | 28 | p2 | (AT)9 | 18 | 85955 | 85972 |
| LHM1005 | *Sanicula_caerulescens* | 29 | p1 | (T)14 | 14 | 86280 | 86293 |
| LHM1005 | *Sanicula_caerulescens* | 30 | p1 | (T)15 | 15 | 100352 | 100366 |
| LHM1005 | *Sanicula_caerulescens* | 31 | p1 | (T)10 | 10 | 104240 | 104249 |
| LHM1005 | *Sanicula_caerulescens* | 32 | p1 | (G)10 | 10 | 105456 | 105465 |
| LHM1005 | *Sanicula_caerulescens* | 33 | p2 | (TA)6 | 12 | 120928 | 120939 |
| LHM1005 | *Sanicula_caerulescens* | 34 | p2 | (AT)8 | 16 | 121163 | 121178 |
| LHM1005 | *Sanicula_caerulescens* | 35 | p1 | (T)13 | 13 | 127903 | 127915 |
| LHM1005 | *Sanicula_caerulescens* | 36 | p1 | (T)11 | 11 | 128181 | 128191 |
| LHM1005 | *Sanicula_caerulescens* | 37 | p1 | (C)10 | 10 | 136502 | 136511 |
| LHM1005 | *Sanicula_caerulescens* | 38 | p1 | (A)10 | 10 | 137718 | 137727 |
| LHM1005 | *Sanicula_caerulescens* | 39 | p1 | (A)15 | 15 | 141601 | 141615 |
| LHM1005 | *Sanicula_caerulescens* | 40 | p1 | (A)14 | 14 | 155674 | 155687 |
| WL3785 | *Sanicula_hacquetiodes* | 1 | p1 | (A)11 | 11 | 3919 | 3929 |
| WL3785 | *Sanicula_hacquetiodes* | 2 | p1 | (A)10 | 10 | 4517 | 4526 |
| WL3785 | *Sanicula_hacquetiodes* | 3 | p2 | (TA)6 | 12 | 9119 | 9130 |
| WL3785 | *Sanicula_hacquetiodes* | 4 | p2 | (AT)9 | 18 | 10553 | 10570 |
| WL3785 | *Sanicula_hacquetiodes* | 5 | p1 | (T)10 | 10 | 10853 | 10862 |
| WL3785 | *Sanicula_hacquetiodes* | 6 | p1 | (A)10 | 10 | 12429 | 12438 |
| WL3785 | *Sanicula_hacquetiodes* | 7 | p1 | (T)10 | 10 | 13139 | 13148 |
| WL3785 | *Sanicula_hacquetiodes* | 8 | p1 | (A)12 | 12 | 13766 | 13777 |
| WL3785 | *Sanicula_hacquetiodes* | 9 | p1 | (A)12 | 12 | 17264 | 17275 |
| WL3785 | *Sanicula_hacquetiodes* | 10 | p1 | (T)11 | 11 | 19498 | 19508 |
| WL3785 | *Sanicula_hacquetiodes* | 11 | p1 | (C)11 | 11 | 23575 | 23585 |
| WL3785 | *Sanicula_hacquetiodes* | 12 | p1 | (T)12 | 12 | 27231 | 27242 |
| WL3785 | *Sanicula_hacquetiodes* | 13 | p2 | (TA)7 | 14 | 28497 | 28510 |
| WL3785 | *Sanicula_hacquetiodes* | 14 | p2 | (AT)6 | 12 | 30614 | 30625 |
| WL3785 | *Sanicula_hacquetiodes* | 15 | p2 | (AT)6 | 12 | 33375 | 33386 |
| WL3785 | *Sanicula_hacquetiodes* | 16 | p2 | (AT)6 | 12 | 33615 | 33626 |
| WL3785 | *Sanicula_hacquetiodes* | 17 | p2 | (TA)7 | 14 | 48006 | 48019 |
| WL3785 | *Sanicula_hacquetiodes* | 18 | p1 | (T)10 | 10 | 55892 | 55901 |
| WL3785 | *Sanicula_hacquetiodes* | 19 | p1 | (T)13 | 13 | 61958 | 61970 |
| WL3785 | *Sanicula_hacquetiodes* | 20 | p1 | (A)10 | 10 | 64965 | 64974 |
| WL3785 | *Sanicula_hacquetiodes* | 21 | p2 | (AT)8 | 16 | 65081 | 65096 |
| WL3785 | *Sanicula_hacquetiodes* | 22 | p1 | (T)10 | 10 | 67029 | 67038 |
| WL3785 | *Sanicula_hacquetiodes* | 23 | p1 | (A)11 | 11 | 67390 | 67400 |
| WL3785 | *Sanicula_hacquetiodes* | 24 | p1 | (A)10 | 10 | 70023 | 70032 |
| WL3785 | *Sanicula_hacquetiodes* | 25 | p1 | (A)11 | 11 | 72287 | 72297 |
| WL3785 | *Sanicula_hacquetiodes* | 26 | p1 | (T)10 | 10 | 79887 | 79896 |
| WL3785 | *Sanicula_hacquetiodes* | 27 | p1 | (T)12 | 12 | 81819 | 81830 |
| WL3785 | *Sanicula_hacquetiodes* | 28 | p2 | (AT)6 | 12 | 85685 | 85696 |
| WL3785 | *Sanicula_hacquetiodes* | 29 | p1 | (T)14 | 14 | 86004 | 86017 |
| WL3785 | *Sanicula_hacquetiodes* | 30 | p1 | (T)10 | 10 | 100091 | 100100 |
| WL3785 | *Sanicula_hacquetiodes* | 31 | p1 | (T)10 | 10 | 104072 | 104081 |
| WL3785 | *Sanicula_hacquetiodes* | 32 | p2 | (AT)7 | 14 | 120980 | 120993 |
| WL3785 | *Sanicula_hacquetiodes* | 33 | p1 | (T)10 | 10 | 122999 | 123008 |
| WL3785 | *Sanicula_hacquetiodes* | 34 | p2 | (TA)6 | 12 | 123280 | 123291 |
| WL3785 | *Sanicula_hacquetiodes* | 35 | p1 | (T)15 | 15 | 127719 | 127733 |
| WL3785 | *Sanicula_hacquetiodes* | 36 | p1 | (T)11 | 11 | 128003 | 128013 |
| WL3785 | *Sanicula_hacquetiodes* | 37 | p1 | (A)10 | 10 | 137539 | 137548 |
| WL3785 | *Sanicula_hacquetiodes* | 38 | p1 | (A)10 | 10 | 141520 | 141529 |
| WL3785 | *Sanicula_hacquetiodes* | 39 | p1 | (A)14 | 14 | 155603 | 155616 |
| LHM1054 | *Sanicula_orthacantha_var._brevispina* | 1 | p2 | (TA)8 | 16 | 148 | 163 |
| LHM1054 | *Sanicula_orthacantha_var._brevispina* | 2 | p1 | (A)10 | 10 | 12245 | 12254 |
| LHM1054 | *Sanicula_orthacantha_var._brevispina* | 3 | p1 | (T)11 | 11 | 12955 | 12965 |
| LHM1054 | *Sanicula_orthacantha_var._brevispina* | 4 | p1 | (A)12 | 12 | 13584 | 13595 |
| LHM1054 | *Sanicula_orthacantha_var._brevispina* | 5 | p1 | (T)11 | 11 | 16258 | 16268 |
| LHM1054 | *Sanicula_orthacantha_var._brevispina* | 6 | p1 | (A)10 | 10 | 17099 | 17108 |
| LHM1054 | *Sanicula_orthacantha_var._brevispina* | 7 | p1 | (T)11 | 11 | 19331 | 19341 |
| LHM1054 | *Sanicula_orthacantha_var._brevispina* | 8 | p1 | (T)12 | 12 | 27065 | 27076 |
| LHM1054 | *Sanicula_orthacantha_var._brevispina* | 9 | p2 | (AT)8 | 16 | 30464 | 30479 |
| LHM1054 | *Sanicula_orthacantha_var._brevispina* | 10 | p1 | (A)12 | 12 | 32231 | 32242 |
| LHM1054 | *Sanicula_orthacantha_var._brevispina* | 11 | p2 | (AT)6 | 12 | 33455 | 33466 |
| LHM1054 | *Sanicula_orthacantha_var._brevispina* | 12 | p1 | (T)10 | 10 | 45214 | 45223 |
| LHM1054 | *Sanicula_orthacantha_var._brevispina* | 13 | p2 | (TA)8 | 16 | 47853 | 47868 |
| LHM1054 | *Sanicula_orthacantha_var._brevispina* | 14 | p1 | (T)10 | 10 | 55721 | 55730 |
| LHM1054 | *Sanicula_orthacantha_var._brevispina* | 15 | p2 | (TA)8 | 16 | 63223 | 63238 |
| LHM1054 | *Sanicula_orthacantha_var._brevispina* | 16 | p1 | (A)12 | 12 | 64790 | 64801 |
| LHM1054 | *Sanicula_orthacantha_var._brevispina* | 17 | p2 | (TA)7 | 14 | 64903 | 64916 |
| LHM1054 | *Sanicula_orthacantha_var._brevispina* | 18 | p1 | (T)10 | 10 | 66855 | 66864 |
| LHM1054 | *Sanicula_orthacantha_var._brevispina* | 19 | p1 | (A)11 | 11 | 67214 | 67224 |
| LHM1054 | *Sanicula_orthacantha_var._brevispina* | 20 | p2 | (TA)7 | 14 | 68136 | 68149 |
| LHM1054 | *Sanicula_orthacantha_var._brevispina* | 21 | p1 | (T)11 | 11 | 71960 | 71970 |
| LHM1054 | *Sanicula_orthacantha_var._brevispina* | 22 | p1 | (A)11 | 11 | 72129 | 72139 |
| LHM1054 | *Sanicula_orthacantha_var._brevispina* | 23 | p1 | (A)11 | 11 | 72770 | 72780 |
| LHM1054 | *Sanicula_orthacantha_var._brevispina* | 24 | p1 | (T)10 | 10 | 79757 | 79766 |
| LHM1054 | *Sanicula_orthacantha_var._brevispina* | 25 | p1 | (T)12 | 12 | 81689 | 81700 |
| LHM1054 | *Sanicula_orthacantha_var._brevispina* | 26 | p2 | (AT)9 | 18 | 85564 | 85581 |
| LHM1054 | *Sanicula_orthacantha_var._brevispina* | 27 | p1 | (T)15 | 15 | 85889 | 85903 |
| LHM1054 | *Sanicula_orthacantha_var._brevispina* | 28 | p1 | (T)15 | 15 | 99977 | 99991 |
| LHM1054 | *Sanicula_orthacantha_var._brevispina* | 29 | p1 | (T)10 | 10 | 103865 | 103874 |
| LHM1054 | *Sanicula_orthacantha_var._brevispina* | 30 | p1 | (G)11 | 11 | 105081 | 105091 |
| LHM1054 | *Sanicula_orthacantha_var._brevispina* | 31 | p2 | (TA)6 | 12 | 120548 | 120559 |
| LHM1054 | *Sanicula_orthacantha_var._brevispina* | 32 | p2 | (AT)6 | 12 | 120783 | 120794 |
| LHM1054 | *Sanicula_orthacantha_var._brevispina* | 33 | p1 | (T)14 | 14 | 127519 | 127532 |
| LHM1054 | *Sanicula_orthacantha_var._brevispina* | 34 | p1 | (T)11 | 11 | 127803 | 127813 |
| LHM1054 | *Sanicula_orthacantha_var._brevispina* | 35 | p1 | (C)11 | 11 | 136124 | 136134 |
| LHM1054 | *Sanicula_orthacantha_var._brevispina* | 36 | p1 | (A)10 | 10 | 137341 | 137350 |
| LHM1054 | *Sanicula_orthacantha_var._brevispina* | 37 | p1 | (A)15 | 15 | 141224 | 141238 |
| LHM1054 | *Sanicula_orthacantha_var._brevispina* | 38 | p1 | (A)15 | 15 | 155312 | 155326 |
| LHM1116 | *Sanicula_tienmuensis* | 1 | p2 | (TA)8 | 16 | 148 | 163 |
| LHM1116 | *Sanicula_tienmuensis* | 2 | p1 | (A)10 | 10 | 3947 | 3956 |
| LHM1116 | *Sanicula_tienmuensis* | 3 | p1 | (A)10 | 10 | 12505 | 12514 |
| LHM1116 | *Sanicula_tienmuensis* | 4 | p1 | (T)11 | 11 | 13208 | 13218 |
| LHM1116 | *Sanicula_tienmuensis* | 5 | p1 | (A)12 | 12 | 13836 | 13847 |
| LHM1116 | *Sanicula_tienmuensis* | 6 | p1 | (A)11 | 11 | 17341 | 17351 |
| LHM1116 | *Sanicula_tienmuensis* | 7 | p1 | (T)11 | 11 | 19574 | 19584 |
| LHM1116 | *Sanicula_tienmuensis* | 8 | p1 | (T)12 | 12 | 27308 | 27319 |
| LHM1116 | *Sanicula_tienmuensis* | 9 | p2 | (AT)8 | 16 | 30692 | 30707 |
| LHM1116 | *Sanicula_tienmuensis* | 10 | p1 | (A)10 | 10 | 32459 | 32468 |
| LHM1116 | *Sanicula_tienmuensis* | 11 | p2 | (AT)10 | 20 | 33721 | 33740 |
| LHM1116 | *Sanicula_tienmuensis* | 12 | p1 | (T)10 | 10 | 45526 | 45535 |
| LHM1116 | *Sanicula_tienmuensis* | 13 | p2 | (TA)7 | 14 | 48153 | 48166 |
| LHM1116 | *Sanicula_tienmuensis* | 14 | p1 | (T)10 | 10 | 55999 | 56008 |
| LHM1116 | *Sanicula_tienmuensis* | 15 | p1 | (T)10 | 10 | 60820 | 60829 |
| LHM1116 | *Sanicula_tienmuensis* | 16 | p2 | (TA)8 | 16 | 63491 | 63506 |
| LHM1116 | *Sanicula_tienmuensis* | 17 | p1 | (A)10 | 10 | 65058 | 65067 |
| LHM1116 | *Sanicula_tienmuensis* | 18 | p1 | (T)10 | 10 | 67115 | 67124 |
| LHM1116 | *Sanicula_tienmuensis* | 19 | p1 | (A)10 | 10 | 67480 | 67489 |
| LHM1116 | *Sanicula_tienmuensis* | 20 | p2 | (TA)7 | 14 | 68401 | 68414 |
| LHM1116 | *Sanicula_tienmuensis* | 21 | p1 | (A)12 | 12 | 72391 | 72402 |
| LHM1116 | *Sanicula_tienmuensis* | 22 | p1 | (A)10 | 10 | 73033 | 73042 |
| LHM1116 | *Sanicula_tienmuensis* | 23 | p1 | (T)10 | 10 | 80013 | 80022 |
| LHM1116 | *Sanicula_tienmuensis* | 24 | p1 | (T)13 | 13 | 81945 | 81957 |
| LHM1116 | *Sanicula_tienmuensis* | 25 | p2 | (AT)9 | 18 | 85821 | 85838 |
| LHM1116 | *Sanicula_tienmuensis* | 26 | p1 | (T)13 | 13 | 86146 | 86158 |
| LHM1116 | *Sanicula_tienmuensis* | 27 | p1 | (T)15 | 15 | 100262 | 100276 |
| LHM1116 | *Sanicula_tienmuensis* | 28 | p1 | (T)10 | 10 | 104150 | 104159 |
| LHM1116 | *Sanicula_tienmuensis* | 29 | p2 | (TA)6 | 12 | 120838 | 120849 |
| LHM1116 | *Sanicula_tienmuensis* | 30 | p2 | (AT)8 | 16 | 121073 | 121088 |
| LHM1116 | *Sanicula_tienmuensis* | 31 | p1 | (T)15 | 15 | 127814 | 127828 |
| LHM1116 | *Sanicula_tienmuensis* | 32 | p1 | (T)11 | 11 | 128098 | 128108 |
| LHM1116 | *Sanicula_tienmuensis* | 33 | p1 | (A)10 | 10 | 137634 | 137643 |
| LHM1116 | *Sanicula_tienmuensis* | 34 | p1 | (A)15 | 15 | 141517 | 141531 |
| LHM1116 | *Sanicula_tienmuensis* | 35 | p1 | (A)13 | 13 | 155635 | 155647 |
