# Supplementary material for: Complete chloroplast of four Sanicula taxa (Apiaceae) endemic to China: lights into genome structure, comparative analysis, and phylogenetic relationships
Source: BMC Plant Biol. 2023 Sep 21;23:444. doi: 10.1186/s12870-023-04447-w (PMC10512634; doi:10.1186/s12870-023-04447-w)
Supplement: Supplementary file 3 — Additional file 3: Table S3. Comparison of dispersed repeats among four newly sequenced Sanicula taxa chloroplast genomes. [file 12870_2023_4447_MOESM3_ESM.docx]

**Table S3**. Comparison of dispersed repeats among four newly sequenced *Sanicula* taxa chloroplast genomes.

| ID | Speceis name | repeat length of the first part | starting position of the first part | match direction | repeat length of the second part | starting position of the second part | distance of this repeat | calculated e-value of this repeat |
| --- | --- | --- | --- | --- | --- | --- | --- | --- |
| LHM1054 | Sanicula orthacantha var. brevispina | 26242 | 85818 | P | 26242 | 129154 | 0 | 0 |
| LHM1054 | Sanicula orthacantha var. brevispina | 50 | 92497 | F | 50 | 92512 | -2 | 5.91E-17 |
| LHM1054 | Sanicula orthacantha var. brevispina | 50 | 92497 | P | 50 | 148652 | -2 | 5.91E-17 |
| LHM1054 | Sanicula orthacantha var. brevispina | 50 | 92512 | P | 50 | 148667 | -2 | 5.91E-17 |
| LHM1054 | Sanicula orthacantha var. brevispina | 50 | 148652 | F | 50 | 148667 | -2 | 5.91E-17 |
| LHM1054 | Sanicula orthacantha var. brevispina | 45 | 75766 | P | 45 | 75766 | -3 | 2.1E-12 |
| LHM1054 | Sanicula orthacantha var. brevispina | 42 | 99592 | F | 42 | 122101 | -1 | 4.42E-14 |
| LHM1054 | Sanicula orthacantha var. brevispina | 42 | 122101 | P | 42 | 141580 | -1 | 4.42E-14 |
| LHM1054 | Sanicula orthacantha var. brevispina | 39 | 44621 | F | 39 | 122103 | -1 | 2.63E-12 |
| LHM1054 | Sanicula orthacantha var. brevispina | 39 | 28323 | R | 39 | 28323 | -2 | 1.5E-10 |
| LHM1054 | Sanicula orthacantha var. brevispina | 39 | 44621 | F | 39 | 99594 | -2 | 1.5E-10 |
| LHM1054 | Sanicula orthacantha var. brevispina | 39 | 44621 | P | 39 | 141581 | -2 | 1.5E-10 |
| LHM1054 | Sanicula orthacantha var. brevispina | 36 | 30663 | P | 36 | 30663 | -2 | 8.15E-09 |
| LHM1054 | Sanicula orthacantha var. brevispina | 35 | 44624 | F | 35 | 96525 | -3 | 1.02E-06 |
| LHM1054 | Sanicula orthacantha var. brevispina | 35 | 44624 | P | 35 | 144654 | -3 | 1.02E-06 |
| LHM1054 | Sanicula orthacantha var. brevispina | 34 | 99600 | F | 34 | 122109 | 0 | 2.3E-11 |
| LHM1054 | Sanicula orthacantha var. brevispina | 34 | 122109 | P | 34 | 141580 | 0 | 2.3E-11 |
| LHM1054 | Sanicula orthacantha var. brevispina | 34 | 108573 | F | 34 | 108605 | -1 | 2.35E-09 |
| LHM1054 | Sanicula orthacantha var. brevispina | 34 | 108573 | P | 34 | 132575 | -1 | 2.35E-09 |
| LHM1054 | Sanicula orthacantha var. brevispina | 34 | 108605 | P | 34 | 132607 | -1 | 2.35E-09 |
| LHM1054 | Sanicula orthacantha var. brevispina | 34 | 132575 | F | 34 | 132607 | -1 | 2.35E-09 |
| LHM1054 | Sanicula orthacantha var. brevispina | 33 | 92499 | F | 33 | 92529 | -1 | 9.11E-09 |
| LHM1054 | Sanicula orthacantha var. brevispina | 33 | 92499 | P | 33 | 148652 | -1 | 9.11E-09 |
| LHM1054 | Sanicula orthacantha var. brevispina | 33 | 92514 | F | 33 | 92529 | -1 | 9.11E-09 |
| LHM1054 | Sanicula orthacantha var. brevispina | 33 | 92514 | P | 33 | 148652 | -1 | 9.11E-09 |
| LHM1054 | Sanicula orthacantha var. brevispina | 33 | 92529 | P | 33 | 148667 | -1 | 9.11E-09 |
| LHM1054 | Sanicula orthacantha var. brevispina | 33 | 92529 | P | 33 | 148682 | -1 | 9.11E-09 |
| LHM1054 | Sanicula orthacantha var. brevispina | 33 | 148652 | F | 33 | 148682 | -1 | 9.11E-09 |
| LHM1054 | Sanicula orthacantha var. brevispina | 31 | 148671 | F | 31 | 148686 | -1 | 1.37E-07 |
| LHM1054 | Sanicula orthacantha var. brevispina | 31 | 47412 | F | 31 | 77911 | -3 | 0.000179 |
| LHM1054 | Sanicula orthacantha var. brevispina | 31 | 78243 | P | 31 | 78264 | -3 | 0.000179 |
| LHM1054 | Sanicula orthacantha var. brevispina | 31 | 108921 | P | 31 | 108921 | -3 | 0.000179 |
| LHM1054 | Sanicula orthacantha var. brevispina | 31 | 108921 | F | 31 | 132262 | -3 | 0.000179 |
| LHM1054 | Sanicula orthacantha var. brevispina | 31 | 132262 | P | 31 | 132262 | -3 | 0.000179 |
| LHM1054 | Sanicula orthacantha var. brevispina | 30 | 8728 | P | 30 | 46350 | 0 | 5.89E-09 |
| LHM1054 | Sanicula orthacantha var. brevispina | 30 | 91209 | F | 30 | 91224 | -1 | 5.3E-07 |
| LHM1054 | Sanicula orthacantha var. brevispina | 30 | 91209 | P | 30 | 149960 | -1 | 5.3E-07 |
| LHM1054 | Sanicula orthacantha var. brevispina | 30 | 91224 | P | 30 | 149975 | -1 | 5.3E-07 |
| LHM1054 | Sanicula orthacantha var. brevispina | 30 | 149960 | F | 30 | 149975 | -1 | 5.3E-07 |
| LHM1054 | Sanicula orthacantha var. brevispina | 30 | 8725 | F | 30 | 36349 | -2 | 2.31E-05 |
| LHM1054 | Sanicula orthacantha var. brevispina | 30 | 68685 | P | 30 | 68728 | -2 | 2.31E-05 |
| LHM1054 | Sanicula orthacantha var. brevispina | 30 | 21454 | F | 30 | 21503 | -3 | 0.000646 |
| LHM1054 | Sanicula orthacantha var. brevispina | 30 | 36269 | F | 30 | 43918 | -3 | 0.000646 |
| LHM1054 | Sanicula orthacantha var. brevispina | 30 | 36352 | P | 30 | 46350 | -3 | 0.000646 |
| LHM1054 | Sanicula orthacantha var. brevispina | 30 | 39566 | F | 30 | 41790 | -3 | 0.000646 |
| LHM1054 | Sanicula orthacantha var. brevispina | 30 | 44622 | P | 30 | 77103 | -3 | 0.000646 |
| WL3785 | Sanicula hacquetiodes | 26332 | 85933 | P | 26332 | 129354 | 0 | 0 |
| WL3785 | Sanicula hacquetiodes | 52 | 78403 | P | 52 | 78403 | -2 | 4.01E-18 |
| WL3785 | Sanicula hacquetiodes | 50 | 92611 | F | 50 | 92626 | -2 | 5.93E-17 |
| WL3785 | Sanicula hacquetiodes | 50 | 92611 | P | 50 | 148943 | -2 | 5.93E-17 |
| WL3785 | Sanicula hacquetiodes | 50 | 92626 | P | 50 | 148958 | -2 | 5.93E-17 |
| WL3785 | Sanicula hacquetiodes | 50 | 148943 | F | 50 | 148958 | -2 | 5.93E-17 |
| WL3785 | Sanicula hacquetiodes | 45 | 75931 | P | 45 | 75931 | -3 | 2.11E-12 |
| WL3785 | Sanicula hacquetiodes | 42 | 99706 | F | 42 | 122300 | -1 | 4.44E-14 |
| WL3785 | Sanicula hacquetiodes | 42 | 122300 | P | 42 | 141871 | -1 | 4.44E-14 |
| WL3785 | Sanicula hacquetiodes | 39 | 44791 | F | 39 | 122302 | -1 | 2.64E-12 |
| WL3785 | Sanicula hacquetiodes | 39 | 44791 | F | 39 | 99708 | -2 | 1.5E-10 |
| WL3785 | Sanicula hacquetiodes | 39 | 44791 | P | 39 | 141872 | -2 | 1.5E-10 |
| WL3785 | Sanicula hacquetiodes | 36 | 30809 | P | 36 | 30809 | -2 | 8.18E-09 |
| WL3785 | Sanicula hacquetiodes | 35 | 44794 | F | 35 | 96639 | -3 | 1.02E-06 |
| WL3785 | Sanicula hacquetiodes | 35 | 44794 | P | 35 | 144945 | -3 | 1.02E-06 |
| WL3785 | Sanicula hacquetiodes | 34 | 99714 | F | 34 | 122308 | 0 | 2.31E-11 |
| WL3785 | Sanicula hacquetiodes | 34 | 122308 | P | 34 | 141871 | 0 | 2.31E-11 |
| WL3785 | Sanicula hacquetiodes | 34 | 108778 | F | 34 | 108810 | -1 | 2.36E-09 |
| WL3785 | Sanicula hacquetiodes | 34 | 108778 | P | 34 | 132775 | -1 | 2.36E-09 |
| WL3785 | Sanicula hacquetiodes | 34 | 108810 | P | 34 | 132807 | -1 | 2.36E-09 |
| WL3785 | Sanicula hacquetiodes | 34 | 132775 | F | 34 | 132807 | -1 | 2.36E-09 |
| WL3785 | Sanicula hacquetiodes | 33 | 92613 | F | 33 | 92643 | -1 | 9.15E-09 |
| WL3785 | Sanicula hacquetiodes | 33 | 92613 | P | 33 | 148943 | -1 | 9.15E-09 |
| WL3785 | Sanicula hacquetiodes | 33 | 92628 | F | 33 | 92643 | -1 | 9.15E-09 |
| WL3785 | Sanicula hacquetiodes | 33 | 92628 | P | 33 | 148943 | -1 | 9.15E-09 |
| WL3785 | Sanicula hacquetiodes | 33 | 92643 | P | 33 | 148958 | -1 | 9.15E-09 |
| WL3785 | Sanicula hacquetiodes | 33 | 92643 | P | 33 | 148973 | -1 | 9.15E-09 |
| WL3785 | Sanicula hacquetiodes | 33 | 148943 | F | 33 | 148973 | -1 | 9.15E-09 |
| WL3785 | Sanicula hacquetiodes | 32 | 48008 | F | 32 | 48040 | 0 | 3.7E-10 |
| WL3785 | Sanicula hacquetiodes | 31 | 148962 | F | 31 | 148977 | -1 | 1.37E-07 |
| WL3785 | Sanicula hacquetiodes | 31 | 47568 | F | 31 | 78071 | -3 | 0.000179 |
| WL3785 | Sanicula hacquetiodes | 31 | 48020 | P | 31 | 48043 | -3 | 0.000179 |
| WL3785 | Sanicula hacquetiodes | 31 | 109126 | P | 31 | 109126 | -3 | 0.000179 |
| WL3785 | Sanicula hacquetiodes | 31 | 109126 | F | 31 | 132462 | -3 | 0.000179 |
| WL3785 | Sanicula hacquetiodes | 31 | 132462 | P | 31 | 132462 | -3 | 0.000179 |
| WL3785 | Sanicula hacquetiodes | 30 | 8943 | P | 30 | 46507 | 0 | 5.91E-09 |
| WL3785 | Sanicula hacquetiodes | 30 | 91323 | F | 30 | 91338 | -1 | 5.32E-07 |
| WL3785 | Sanicula hacquetiodes | 30 | 91323 | P | 30 | 150251 | -1 | 5.32E-07 |
| WL3785 | Sanicula hacquetiodes | 30 | 91338 | P | 30 | 150266 | -1 | 5.32E-07 |
| WL3785 | Sanicula hacquetiodes | 30 | 150251 | F | 30 | 150266 | -1 | 5.32E-07 |
| WL3785 | Sanicula hacquetiodes | 30 | 8940 | F | 30 | 36536 | -2 | 2.31E-05 |
| WL3785 | Sanicula hacquetiodes | 30 | 36539 | P | 30 | 46507 | -3 | 0.000648 |
| WL3785 | Sanicula hacquetiodes | 30 | 39744 | F | 30 | 41968 | -3 | 0.000648 |
| WL3785 | Sanicula hacquetiodes | 30 | 44792 | P | 30 | 77263 | -3 | 0.000648 |
| LHM1005 | Sanicla caeruelscens | 30 | 8958 | P | 30 | 46686 | 0 | 5.92E-09 |
| LHM1005 | Sanicla caeruelscens | 30 | 8955 | F | 30 | 36685 | -2 | 2.32E-05 |
| LHM1005 | Sanicla caeruelscens | 30 | 69042 | P | 30 | 69085 | -2 | 2.32E-05 |
| LHM1005 | Sanicla caeruelscens | 30 | 21711 | F | 30 | 21760 | -3 | 0.000649 |
| LHM1005 | Sanicla caeruelscens | 30 | 36688 | P | 30 | 46686 | -3 | 0.000649 |
| LHM1005 | Sanicla caeruelscens | 30 | 39902 | F | 30 | 42126 | -3 | 0.000649 |
| LHM1005 | Sanicla caeruelscens | 30 | 44958 | P | 30 | 77494 | -3 | 0.000649 |
| LHM1005 | Sanicla caeruelscens | 31 | 149048 | F | 31 | 149063 | -1 | 1.38E-07 |
| LHM1005 | Sanicla caeruelscens | 31 | 47748 | F | 31 | 78302 | -3 | 0.00018 |
| LHM1005 | Sanicla caeruelscens | 31 | 78634 | P | 31 | 78655 | -3 | 0.00018 |
| LHM1005 | Sanicla caeruelscens | 31 | 109295 | P | 31 | 109295 | -3 | 0.00018 |
| LHM1005 | Sanicla caeruelscens | 31 | 109295 | F | 31 | 132640 | -3 | 0.00018 |
| LHM1005 | Sanicla caeruelscens | 31 | 132640 | P | 31 | 132640 | -3 | 0.00018 |
| LHM1005 | Sanicla caeruelscens | 33 | 92874 | F | 33 | 92904 | -1 | 9.15E-09 |
| LHM1005 | Sanicla caeruelscens | 33 | 92874 | P | 33 | 149029 | -1 | 9.15E-09 |
| LHM1005 | Sanicla caeruelscens | 33 | 92889 | F | 33 | 92904 | -1 | 9.15E-09 |
| LHM1005 | Sanicla caeruelscens | 33 | 92889 | P | 33 | 149029 | -1 | 9.15E-09 |
| LHM1005 | Sanicla caeruelscens | 33 | 92904 | P | 33 | 149044 | -1 | 9.15E-09 |
| LHM1005 | Sanicla caeruelscens | 33 | 92904 | P | 33 | 149059 | -1 | 9.15E-09 |
| LHM1005 | Sanicla caeruelscens | 33 | 149029 | F | 33 | 149059 | -1 | 9.15E-09 |
| LHM1005 | Sanicla caeruelscens | 34 | 99975 | F | 34 | 122493 | 0 | 2.31E-11 |
| LHM1005 | Sanicla caeruelscens | 34 | 122493 | P | 34 | 141957 | 0 | 2.31E-11 |
| LHM1005 | Sanicla caeruelscens | 34 | 108947 | F | 34 | 108979 | -1 | 2.36E-09 |
| LHM1005 | Sanicla caeruelscens | 34 | 108947 | P | 34 | 132953 | -1 | 2.36E-09 |
| LHM1005 | Sanicla caeruelscens | 34 | 108979 | P | 34 | 132985 | -1 | 2.36E-09 |
| LHM1005 | Sanicla caeruelscens | 34 | 132953 | F | 34 | 132985 | -1 | 2.36E-09 |
| LHM1005 | Sanicla caeruelscens | 35 | 44960 | F | 35 | 96900 | -3 | 1.02E-06 |
| LHM1005 | Sanicla caeruelscens | 35 | 44960 | P | 35 | 145031 | -3 | 1.02E-06 |
| LHM1005 | Sanicla caeruelscens | 36 | 30906 | P | 36 | 30906 | -2 | 8.19E-09 |
| LHM1005 | Sanicla caeruelscens | 39 | 44957 | F | 39 | 122487 | -1 | 2.64E-12 |
| LHM1005 | Sanicla caeruelscens | 39 | 28580 | R | 39 | 28580 | -2 | 1.51E-10 |
| LHM1005 | Sanicla caeruelscens | 39 | 44957 | F | 39 | 99969 | -2 | 1.51E-10 |
| LHM1005 | Sanicla caeruelscens | 39 | 44957 | P | 39 | 141958 | -2 | 1.51E-10 |
| LHM1005 | Sanicla caeruelscens | 40 | 32816 | F | 40 | 32856 | 0 | 5.64E-15 |
| LHM1005 | Sanicla caeruelscens | 42 | 99967 | F | 42 | 122485 | -1 | 4.44E-14 |
| LHM1005 | Sanicla caeruelscens | 42 | 122485 | P | 42 | 141957 | -1 | 4.44E-14 |
| LHM1005 | Sanicla caeruelscens | 45 | 76157 | P | 45 | 76157 | -3 | 2.11E-12 |
| LHM1005 | Sanicla caeruelscens | 50 | 92872 | F | 50 | 92887 | -2 | 5.93E-17 |
| LHM1005 | Sanicla caeruelscens | 50 | 92872 | P | 50 | 149029 | -2 | 5.93E-17 |
| LHM1005 | Sanicla caeruelscens | 50 | 92887 | P | 50 | 149044 | -2 | 5.93E-17 |
| LHM1005 | Sanicla caeruelscens | 50 | 149029 | F | 50 | 149044 | -2 | 5.93E-17 |
| LHM1005 | Sanicla caeruelscens | 26225 | 86209 | P | 26225 | 129532 | 0 | 0 |
| LHM1116 | Sanicula tienmuensis | 30 | 8981 | P | 30 | 46648 | 0 | 5.92E-09 |
| LHM1116 | Sanicula tienmuensis | 30 | 8978 | F | 30 | 36664 | -2 | 2.32E-05 |
| LHM1116 | Sanicula tienmuensis | 30 | 68950 | P | 30 | 68993 | -2 | 2.32E-05 |
| LHM1116 | Sanicula tienmuensis | 30 | 91464 | F | 30 | 91479 | -2 | 2.32E-05 |
| LHM1116 | Sanicula tienmuensis | 30 | 91464 | P | 30 | 150283 | -2 | 2.32E-05 |
| LHM1116 | Sanicula tienmuensis | 30 | 91479 | P | 30 | 150298 | -2 | 2.32E-05 |
| LHM1116 | Sanicula tienmuensis | 30 | 150283 | F | 30 | 150298 | -2 | 2.32E-05 |
| LHM1116 | Sanicula tienmuensis | 30 | 145 | R | 30 | 121060 | -3 | 0.000648 |
| LHM1116 | Sanicula tienmuensis | 30 | 147 | C | 30 | 148 | -3 | 0.000648 |
| LHM1116 | Sanicula tienmuensis | 30 | 21697 | F | 30 | 21746 | -3 | 0.000648 |
| LHM1116 | Sanicula tienmuensis | 30 | 36538 | F | 30 | 44225 | -3 | 0.000648 |
| LHM1116 | Sanicula tienmuensis | 30 | 36667 | P | 30 | 46648 | -3 | 0.000648 |
| LHM1116 | Sanicula tienmuensis | 30 | 39873 | F | 30 | 42097 | -3 | 0.000648 |
| LHM1116 | Sanicula tienmuensis | 30 | 44929 | P | 30 | 77377 | -3 | 0.000648 |
| LHM1116 | Sanicula tienmuensis | 30 | 114995 | R | 30 | 115003 | -3 | 0.000648 |
| LHM1116 | Sanicula tienmuensis | 31 | 148964 | F | 31 | 149009 | -1 | 1.38E-07 |
| LHM1116 | Sanicula tienmuensis | 31 | 148994 | F | 31 | 149009 | -1 | 1.38E-07 |
| LHM1116 | Sanicula tienmuensis | 31 | 47710 | F | 31 | 78185 | -3 | 0.000179 |
| LHM1116 | Sanicula tienmuensis | 31 | 78517 | P | 31 | 78538 | -3 | 0.000179 |
| LHM1116 | Sanicula tienmuensis | 31 | 109204 | P | 31 | 109204 | -3 | 0.000179 |
| LHM1116 | Sanicula tienmuensis | 31 | 109204 | F | 31 | 132557 | -3 | 0.000179 |
| LHM1116 | Sanicula tienmuensis | 31 | 132557 | P | 31 | 132557 | -3 | 0.000179 |
| LHM1116 | Sanicula tienmuensis | 33 | 92754 | F | 33 | 92814 | -1 | 9.15E-09 |
| LHM1116 | Sanicula tienmuensis | 33 | 92754 | P | 33 | 148945 | -1 | 9.15E-09 |
| LHM1116 | Sanicula tienmuensis | 33 | 92769 | F | 33 | 92814 | -1 | 9.15E-09 |
| LHM1116 | Sanicula tienmuensis | 33 | 92769 | P | 33 | 148945 | -1 | 9.15E-09 |
| LHM1116 | Sanicula tienmuensis | 33 | 92799 | F | 33 | 92814 | -1 | 9.15E-09 |
| LHM1116 | Sanicula tienmuensis | 33 | 92799 | P | 33 | 148945 | -1 | 9.15E-09 |
| LHM1116 | Sanicula tienmuensis | 33 | 92814 | P | 33 | 148960 | -1 | 9.15E-09 |
| LHM1116 | Sanicula tienmuensis | 33 | 92814 | P | 33 | 148990 | -1 | 9.15E-09 |
| LHM1116 | Sanicula tienmuensis | 33 | 92814 | P | 33 | 149005 | -1 | 9.15E-09 |
| LHM1116 | Sanicula tienmuensis | 33 | 148945 | F | 33 | 149005 | -1 | 9.15E-09 |
| LHM1116 | Sanicula tienmuensis | 34 | 99885 | F | 34 | 122403 | 0 | 2.31E-11 |
| LHM1116 | Sanicula tienmuensis | 34 | 122403 | P | 34 | 141873 | 0 | 2.31E-11 |
| LHM1116 | Sanicula tienmuensis | 34 | 108856 | F | 34 | 108888 | -1 | 2.36E-09 |
| LHM1116 | Sanicula tienmuensis | 34 | 108856 | P | 34 | 132870 | -1 | 2.36E-09 |
| LHM1116 | Sanicula tienmuensis | 34 | 108888 | P | 34 | 132902 | -1 | 2.36E-09 |
| LHM1116 | Sanicula tienmuensis | 34 | 132870 | F | 34 | 132902 | -1 | 2.36E-09 |
| LHM1116 | Sanicula tienmuensis | 35 | 44931 | F | 35 | 96810 | -3 | 1.02E-06 |
| LHM1116 | Sanicula tienmuensis | 35 | 44931 | P | 35 | 144947 | -3 | 1.02E-06 |
| LHM1116 | Sanicula tienmuensis | 36 | 30891 | P | 36 | 30891 | -2 | 8.19E-09 |
| LHM1116 | Sanicula tienmuensis | 39 | 44928 | F | 39 | 122397 | -1 | 2.64E-12 |
| LHM1116 | Sanicula tienmuensis | 39 | 44928 | F | 39 | 99879 | -2 | 1.5E-10 |
| LHM1116 | Sanicula tienmuensis | 39 | 44928 | P | 39 | 141874 | -2 | 1.5E-10 |
| LHM1116 | Sanicula tienmuensis | 40 | 32783 | F | 40 | 32823 | 0 | 5.64E-15 |
| LHM1116 | Sanicula tienmuensis | 42 | 99877 | F | 42 | 122395 | -1 | 4.44E-14 |
| LHM1116 | Sanicula tienmuensis | 42 | 122395 | P | 42 | 141873 | -1 | 4.44E-14 |
| LHM1116 | Sanicula tienmuensis | 46 | 148979 | F | 46 | 148994 | -2 | 1.28E-14 |
| LHM1116 | Sanicula tienmuensis | 48 | 92784 | F | 48 | 92799 | -2 | 8.74E-16 |
| LHM1116 | Sanicula tienmuensis | 48 | 92784 | P | 48 | 148945 | -2 | 8.74E-16 |
| LHM1116 | Sanicula tienmuensis | 48 | 92799 | P | 48 | 148960 | -2 | 8.74E-16 |
| LHM1116 | Sanicula tienmuensis | 50 | 92752 | F | 50 | 92797 | -2 | 5.93E-17 |
| LHM1116 | Sanicula tienmuensis | 50 | 92752 | P | 50 | 148945 | -2 | 5.93E-17 |
| LHM1116 | Sanicula tienmuensis | 50 | 92797 | P | 50 | 148990 | -2 | 5.93E-17 |
| LHM1116 | Sanicula tienmuensis | 50 | 148945 | F | 50 | 148990 | -2 | 5.93E-17 |
| LHM1116 | Sanicula tienmuensis | 59 | 92754 | F | 59 | 92784 | 0 | 2.05E-26 |
| LHM1116 | Sanicula tienmuensis | 59 | 92754 | P | 59 | 148949 | 0 | 2.05E-26 |
| LHM1116 | Sanicula tienmuensis | 59 | 92784 | P | 59 | 148979 | 0 | 2.05E-26 |
| LHM1116 | Sanicula tienmuensis | 59 | 148949 | F | 59 | 148979 | 0 | 2.05E-26 |
| LHM1116 | Sanicula tienmuensis | 61 | 92752 | F | 61 | 92767 | -3 | 1.25E-21 |
| LHM1116 | Sanicula tienmuensis | 61 | 92752 | P | 61 | 148964 | -3 | 1.25E-21 |
| LHM1116 | Sanicula tienmuensis | 61 | 92767 | P | 61 | 148979 | -3 | 1.25E-21 |
| LHM1116 | Sanicula tienmuensis | 61 | 148964 | F | 61 | 148979 | -3 | 1.25E-21 |
| LHM1116 | Sanicula tienmuensis | 63 | 92769 | F | 63 | 92784 | -3 | 8.6E-23 |
| LHM1116 | Sanicula tienmuensis | 63 | 92769 | P | 63 | 148945 | -3 | 8.6E-23 |
| LHM1116 | Sanicula tienmuensis | 63 | 92784 | P | 63 | 148960 | -3 | 8.6E-23 |
| LHM1116 | Sanicula tienmuensis | 63 | 148945 | F | 63 | 148960 | -3 | 8.6E-23 |
| LHM1116 | Sanicula tienmuensis | 26268 | 86075 | P | 26268 | 129449 | 0 | 0 |
