# Supplementary material for: Complete chloroplast of four Sanicula taxa (Apiaceae) endemic to China: lights into genome structure, comparative analysis, and phylogenetic relationships
Source: BMC Plant Biol. 2023 Sep 21;23:444. doi: 10.1186/s12870-023-04447-w (PMC10512634; doi:10.1186/s12870-023-04447-w)
Supplement: Supplementary file 4 — Additional file 4: Table S4. Codon usage and relative synonymous codon usage (RSCU) values of protein-coding genes of the four newly sequenced Sanicula chloroplast genomes. [file 12870_2023_4447_MOESM4_ESM.docx]

**Table S4**. Codon usage and relative synonymous codon usage (RSCU) values of protein-coding genes of the 4 *Sanicula* chloroplast genomes.

|  | **Codon** | ***Sanicula caerulescens*_LHM1005** | | ***Sanicula hacquetiodes*_WL3785** | | ***Sanicula orthacantha* var. *brevispina*_LHM1054** | | ***Sanicula tienmuensis*_LHM1116** | |
| --- | --- | --- | --- | --- | --- | --- | --- | --- | --- |
|  |  | **Count** | **RSCU** | **Count** | **RSCU** | **Count** | **RSCU** | **Count** | **RSCU** |
| Stop | TAA | 38 | 1.443038 | 39 | 1.481013 | 38 | 1.443038 | 38 | 1.443038 |
|  | TAG | 20 | 0.759494 | 20 | 0.759494 | 20 | 0.759494 | 20 | 0.759494 |
|  | TGA | 21 | 0.797468 | 20 | 0.759494 | 21 | 0.797468 | 21 | 0.797468 |
| Ala | GCT | 567 | 1.759503 | 568 | 1.757154 | 567 | 1.759503 | 568 | 1.762607 |
|  | GCC | 229 | 0.710628 | 229 | 0.70843 | 229 | 0.710628 | 229 | 0.710628 |
|  | GCA | 341 | 1.058185 | 341 | 1.054911 | 340 | 1.055081 | 339 | 1.051978 |
|  | GCG | 152 | 0.471683 | 155 | 0.479505 | 153 | 0.474787 | 153 | 0.474787 |
| Arg | CGT | 313 | 1.387001 | 314 | 1.391433 | 313 | 1.387001 | 313 | 1.387001 |
|  | CGC | 86 | 0.381093 | 83 | 0.367799 | 86 | 0.381093 | 87 | 0.385524 |
|  | CGA | 314 | 1.391433 | 318 | 1.409158 | 314 | 1.391433 | 314 | 1.391433 |
|  | CGG | 105 | 0.465288 | 104 | 0.460857 | 105 | 0.465288 | 104 | 0.460857 |
|  | AGA | 397 | 1.759232 | 394 | 1.745938 | 397 | 1.759232 | 397 | 1.759232 |
|  | AGG | 139 | 0.615953 | 141 | 0.624815 | 139 | 0.615953 | 139 | 0.615953 |
| Asn | AAT | 782 | 1.512573 | 784 | 1.52381 | 783 | 1.513043 | 781 | 1.513566 |
|  | AAC | 252 | 0.487427 | 245 | 0.47619 | 252 | 0.486957 | 251 | 0.486434 |
| Asp | GAT | 703 | 1.597727 | 700 | 1.589103 | 704 | 1.598184 | 710 | 1.600902 |
|  | GAC | 177 | 0.402273 | 181 | 0.410897 | 177 | 0.401816 | 177 | 0.399098 |
| Cys | TGT | 183 | 1.544304 | 185 | 1.548117 | 184 | 1.546218 | 183 | 1.544304 |
|  | TGC | 54 | 0.455696 | 54 | 0.451883 | 54 | 0.453782 | 54 | 0.455696 |
| Gln | CAA | 613 | 1.51358 | 609 | 1.511166 | 613 | 1.51358 | 614 | 1.516049 |
|  | CAG | 197 | 0.48642 | 197 | 0.488834 | 197 | 0.48642 | 196 | 0.483951 |
| Glu | GAA | 888 | 1.50381 | 888 | 1.505085 | 889 | 1.50423 | 888 | 1.50381 |
|  | GAG | 293 | 0.49619 | 292 | 0.494915 | 293 | 0.49577 | 293 | 0.49619 |
| Gly | GGT | 544 | 1.34404 | 544 | 1.340727 | 544 | 1.34487 | 544 | 1.34404 |
|  | GGC | 192 | 0.474367 | 192 | 0.473198 | 192 | 0.47466 | 192 | 0.474367 |
|  | GGA | 600 | 1.482397 | 606 | 1.49353 | 600 | 1.483313 | 601 | 1.484867 |
|  | GGG | 283 | 0.699197 | 281 | 0.692545 | 282 | 0.697157 | 282 | 0.696726 |
| His | CAT | 396 | 1.488722 | 399 | 1.486034 | 396 | 1.485929 | 397 | 1.489681 |
|  | CAC | 136 | 0.511278 | 138 | 0.513966 | 137 | 0.514071 | 136 | 0.510319 |
| Ile | ATT | 912 | 1.433211 | 911 | 1.429393 | 912 | 1.430962 | 915 | 1.431178 |
|  | ATC | 399 | 0.62703 | 396 | 0.621339 | 401 | 0.629184 | 400 | 0.625652 |
|  | ATA | 598 | 0.939759 | 605 | 0.949268 | 599 | 0.939854 | 603 | 0.94317 |
| Leu | TTA | 764 | 1.918794 | 758 | 1.90932 | 763 | 1.915481 | 762 | 1.913771 |
|  | TTG | 493 | 1.238175 | 491 | 1.236776 | 495 | 1.242678 | 494 | 1.240686 |
|  | CTT | 510 | 1.280871 | 511 | 1.287154 | 509 | 1.277824 | 508 | 1.275848 |
|  | CTC | 153 | 0.384261 | 155 | 0.390428 | 153 | 0.3841 | 153 | 0.384261 |
|  | CTA | 320 | 0.803684 | 319 | 0.803526 | 322 | 0.808368 | 323 | 0.811218 |
|  | CTG | 149 | 0.374215 | 148 | 0.372796 | 148 | 0.371548 | 149 | 0.374215 |
| Lys | AAA | 877 | 1.486441 | 883 | 1.490295 | 878 | 1.488136 | 878 | 1.485618 |
|  | AAG | 303 | 0.513559 | 302 | 0.509705 | 302 | 0.511864 | 304 | 0.514382 |
| Met | TTG | 0 | 0 | 0 | 0 | 0 | 0 | 0 | 0 |
|  | CTG | 1 | 0.012891 | 1 | 0.012963 | 1 | 0.012891 | 1 | 0.012939 |
|  | ATT | 0 | 0 | 0 | 0 | 0 | 0 | 0 | 0 |
|  | ATC | 0 | 0 | 0 | 0 | 0 | 0 | 0 | 0 |
|  | ATA | 0 | 0 | 0 | 0 | 0 | 0 | 0 | 0 |
|  | ATG | 541 | 6.974217 | 538 | 6.974074 | 541 | 6.974217 | 539 | 6.974122 |
|  | GTG | 1 | 0.012891 | 1 | 0.012963 | 1 | 0.012891 | 1 | 0.012939 |
| Phe | TTT | 830 | 1.30094 | 832 | 1.3 | 829 | 1.299373 | 829 | 1.301413 |
|  | TTC | 446 | 0.69906 | 448 | 0.7 | 447 | 0.700627 | 445 | 0.698587 |
| Pro | CCT | 386 | 1.593395 | 385 | 1.587629 | 386 | 1.591753 | 385 | 1.587629 |
|  | CCC | 170 | 0.701754 | 171 | 0.705155 | 170 | 0.701031 | 171 | 0.705155 |
|  | CCA | 268 | 1.106295 | 267 | 1.101031 | 268 | 1.105155 | 268 | 1.105155 |
|  | CCG | 145 | 0.598555 | 147 | 0.606186 | 146 | 0.602062 | 146 | 0.602062 |
| Ser | TCT | 498 | 1.758682 | 497 | 1.759292 | 499 | 1.762213 | 499 | 1.761176 |
|  | TCC | 263 | 0.928782 | 259 | 0.916814 | 263 | 0.928782 | 264 | 0.931765 |
|  | TCA | 322 | 1.137139 | 322 | 1.139823 | 322 | 1.137139 | 323 | 1.14 |
|  | TCG | 170 | 0.600353 | 171 | 0.60531 | 169 | 0.596822 | 167 | 0.589412 |
|  | AGT | 347 | 1.225427 | 346 | 1.224779 | 347 | 1.225427 | 348 | 1.228235 |
|  | AGC | 99 | 0.349617 | 100 | 0.353982 | 99 | 0.349617 | 99 | 0.349412 |
| Thr | ACT | 490 | 1.647059 | 491 | 1.653199 | 490 | 1.648444 | 490 | 1.644295 |
|  | ACC | 221 | 0.742857 | 220 | 0.740741 | 221 | 0.743482 | 221 | 0.741611 |
|  | ACA | 348 | 1.169748 | 345 | 1.161616 | 346 | 1.164003 | 347 | 1.16443 |
|  | ACG | 131 | 0.440336 | 132 | 0.444444 | 132 | 0.444071 | 134 | 0.449664 |
| Trp | TGG | 406 | 1 | 406 | 1 | 406 | 1 | 405 | 1 |
| Tyr | TAT | 682 | 1.619952 | 685 | 1.623223 | 681 | 1.619501 | 681 | 1.617577 |
|  | TAC | 160 | 0.380048 | 159 | 0.376777 | 160 | 0.380499 | 161 | 0.382423 |
| Val | GTT | 465 | 1.482072 | 465 | 1.482072 | 465 | 1.482072 | 466 | 1.484076 |
|  | GTC | 151 | 0.481275 | 151 | 0.481275 | 151 | 0.481275 | 151 | 0.480892 |
|  | GTA | 455 | 1.450199 | 455 | 1.450199 | 455 | 1.450199 | 455 | 1.449045 |
|  | GTG | 184 | 0.586454 | 184 | 0.586454 | 184 | 0.586454 | 184 | 0.585987 |
| Total |  | 22673 |  | 22678 |  | 22680 |  | 22690 |  |
