# Supplementary figures and images for: Complete chloroplast of four Sanicula taxa (Apiaceae) endemic to China: lights into genome structure, comparative analysis, and phylogenetic relationships
Source: BMC Plant Biol. 2023 Sep 21;23:444. doi: 10.1186/s12870-023-04447-w (PMC10512634; doi:10.1186/s12870-023-04447-w)

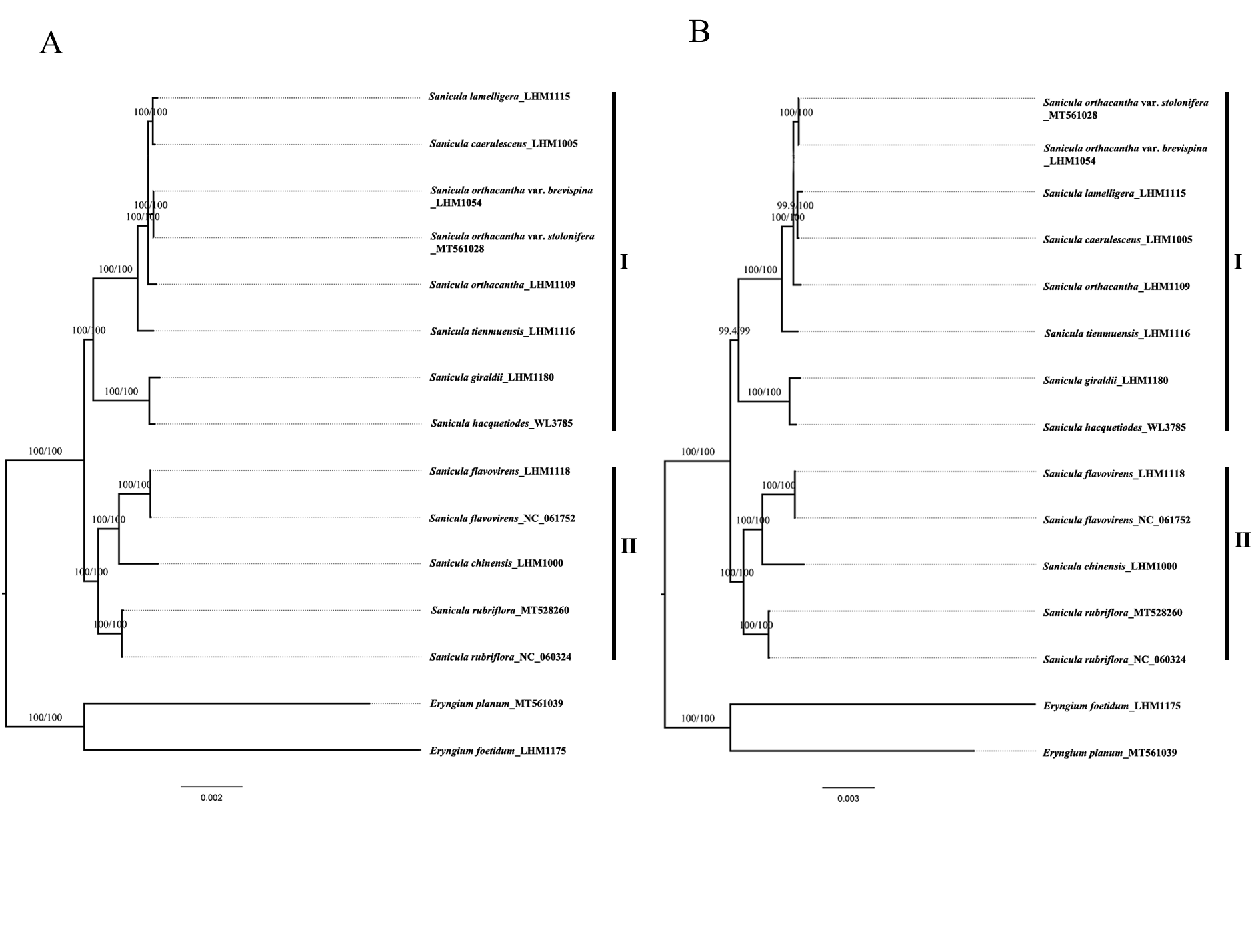

Supplement: Supplementary file 5 — Additional file 5:Fig. S1. Collecting information, voucher specimen and identification for the nine taxa of Sanicula L. and one species of Eryngium L. in the study. [file 12870_2023_4447_MOESM5_ESM.tif]

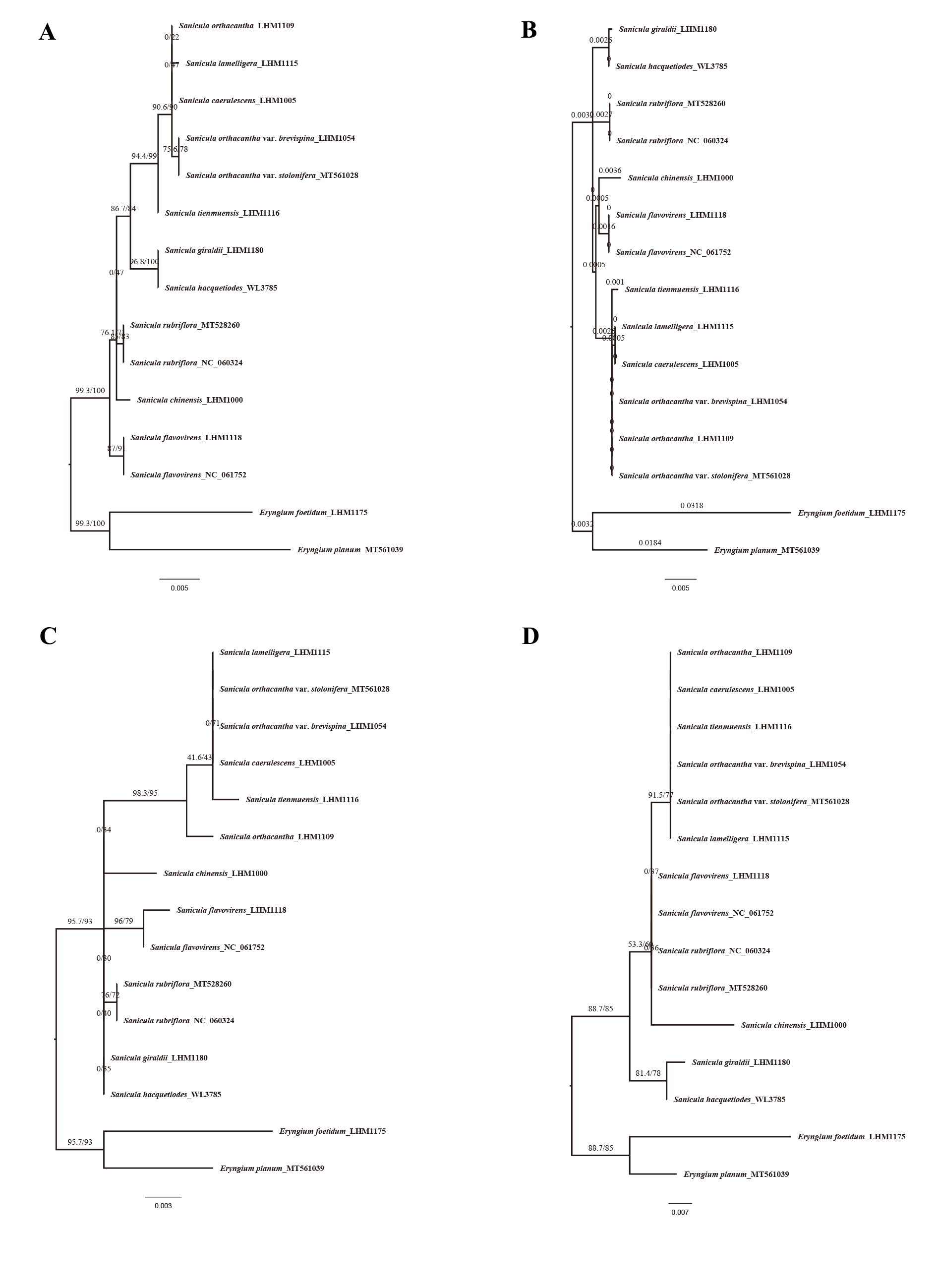

Supplement: Supplementary file 6 — Additional file 6: Fig. S2. Phylogenetic relationships of 13 Sanicula samples and two Eryngium species inferred from maximum likelihood (ML) analysis. A. The whole cp genome. B. Concatenation of 126 unique IGS regions. [file 12870_2023_4447_MOESM6_ESM.tif]

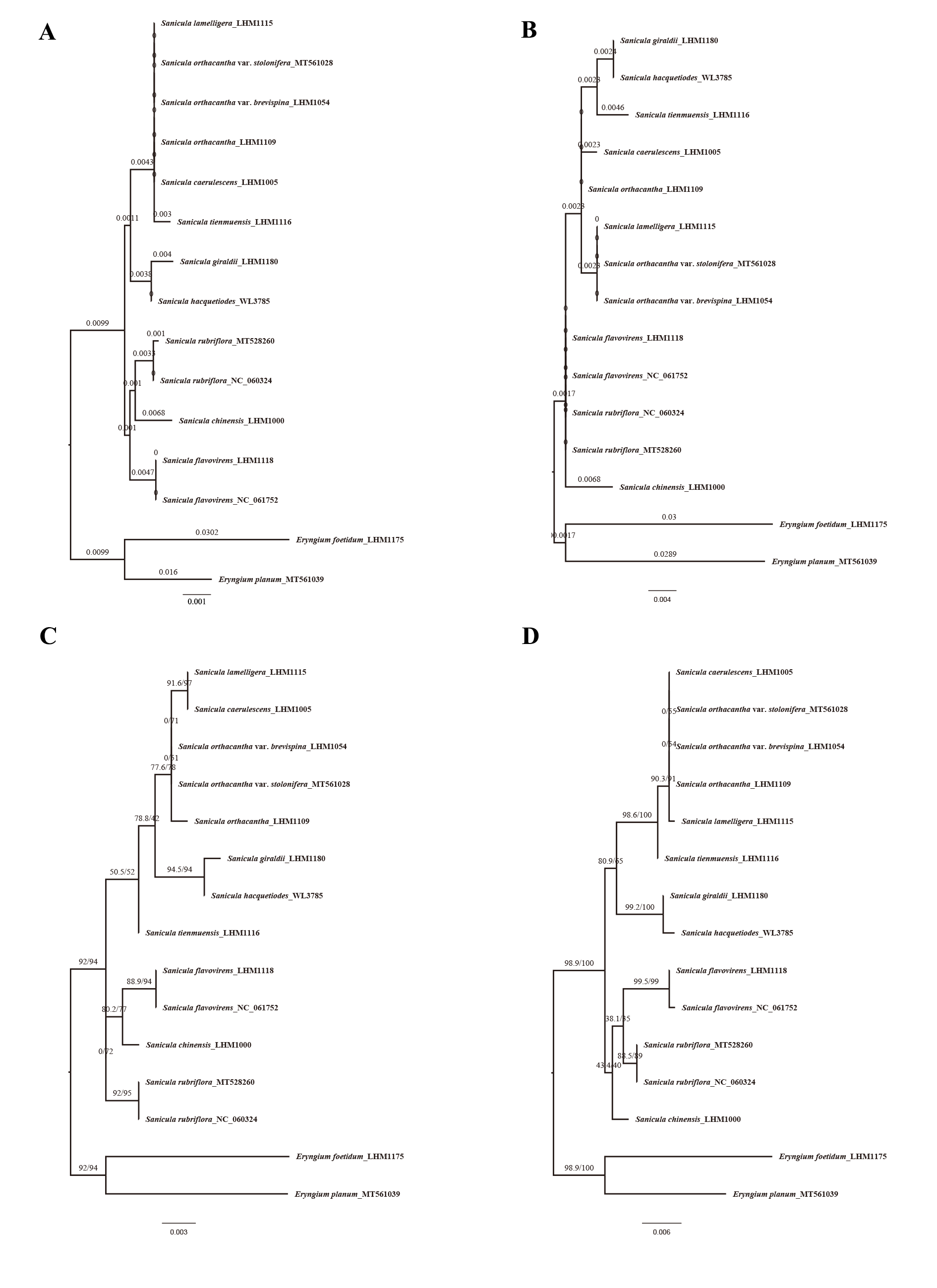

Supplement: Supplementary file 7 — Additional file 7: Fig. S3 Phylogenetic relationships of 13 Sanicula samples and two Eryngium species inferred from maximum likelihood (ML) analysis. A. atpH-atpI. B. ndhC-trnM. C. petB-petD. D. petD-rpoA. [file 12870_2023_4447_MOESM7_ESM.tif]

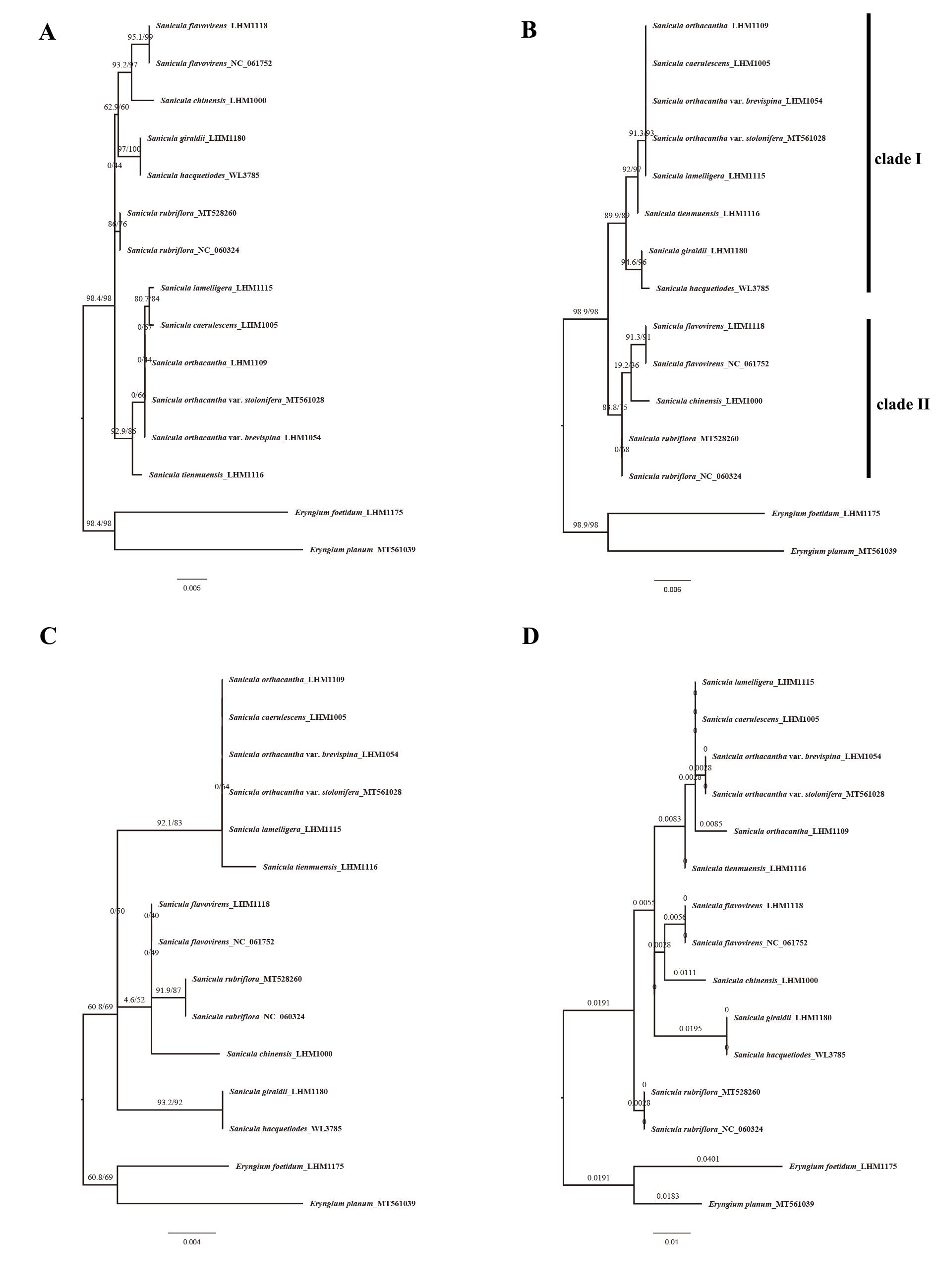

Supplement: Supplementary file 8 — Additional file 8: Fig. S4. Phylogenetic relationships of 13 Sanicula samples and two Eryngium species inferred from maximum likelihood (ML) analysis. A. petN-psbM. B. psaJ-rpl33. C. rbcL-accD. D. rpoB-trnC. [file 12870_2023_4447_MOESM8_ESM.tif]

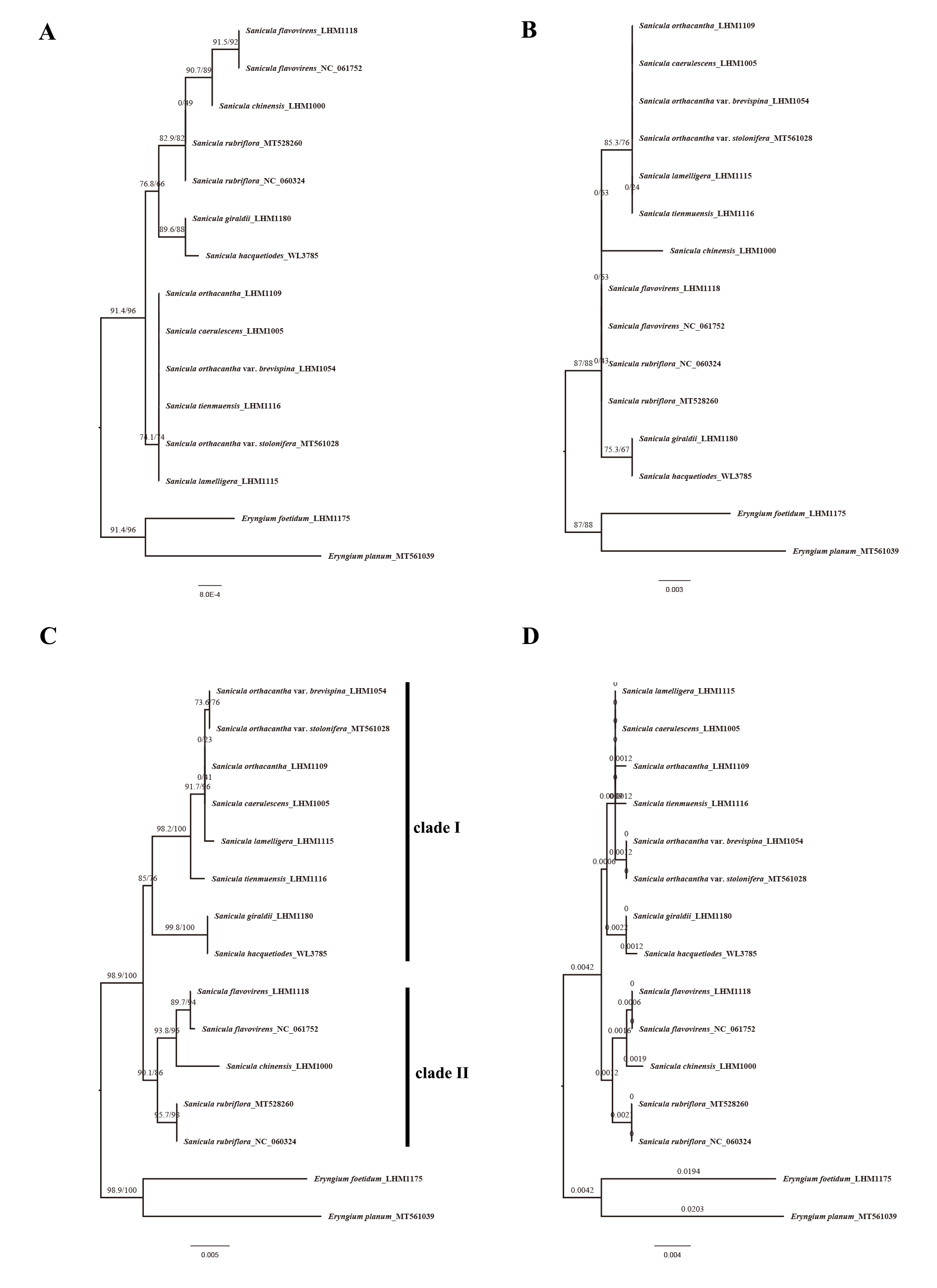

Supplement: Supplementary file 9 — Additional file 9: Fig. S5. Phylogenetic relationships of 13 Sanicula samples and two Eryngium species inferred from maximum likelihood (ML) analysis. A. rps16-trnQ. B. trnE-psbD. C. trnF-ndhJ. D. trnH-psbA. [file 12870_2023_4447_MOESM9_ESM.tif]

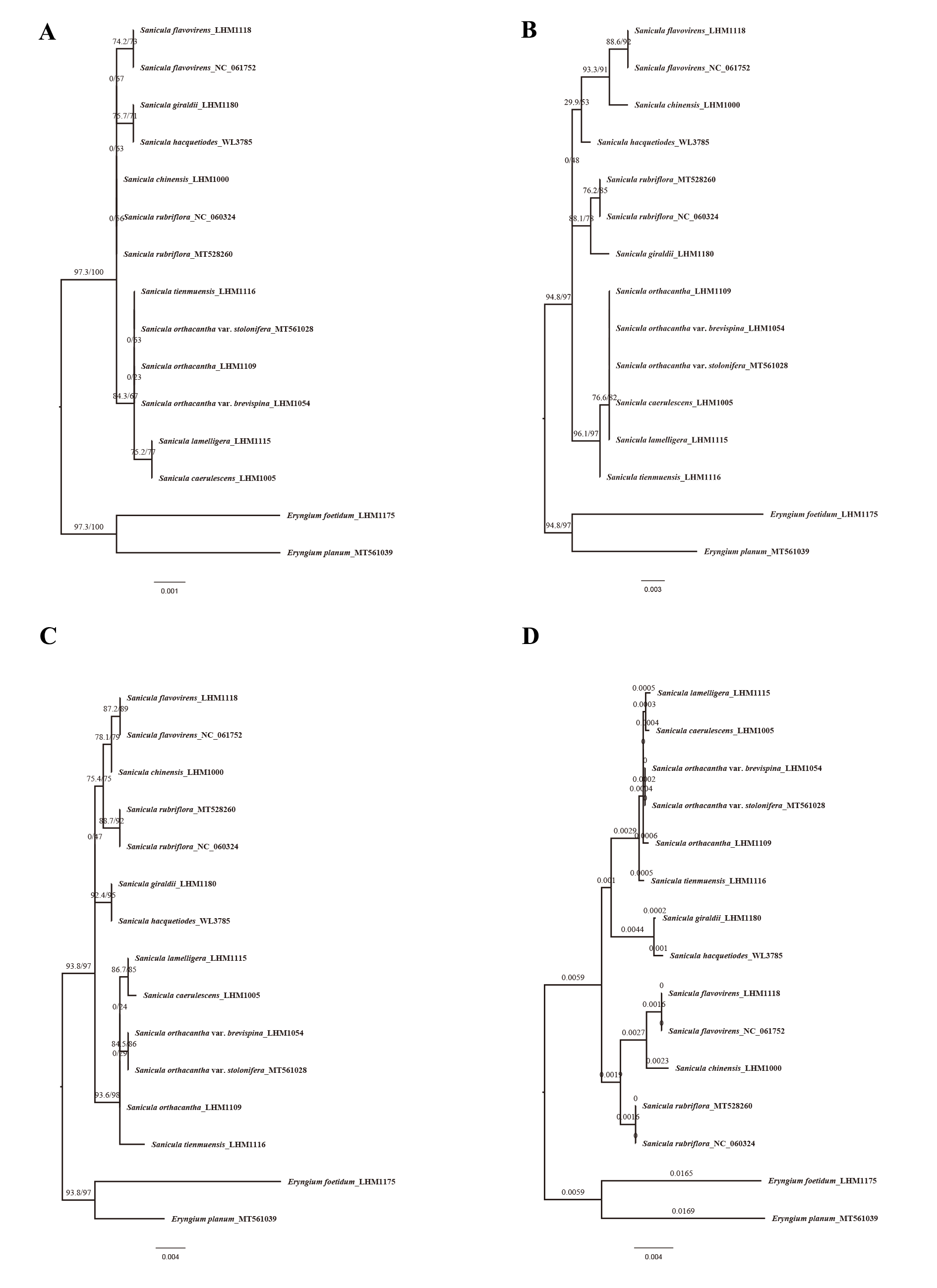

Supplement: Supplementary file 10 — Additional file 10: Fig. S6. Phylogenetic relationships of 13 Sanicula samples and two Eryngium species inferred from maximum likelihood (ML) analysis. A. trnN-ndhF. B. trnS-psbZ. C. trnS-trnR. D. trnT-trnF. [file 12870_2023_4447_MOESM10_ESM.tif]

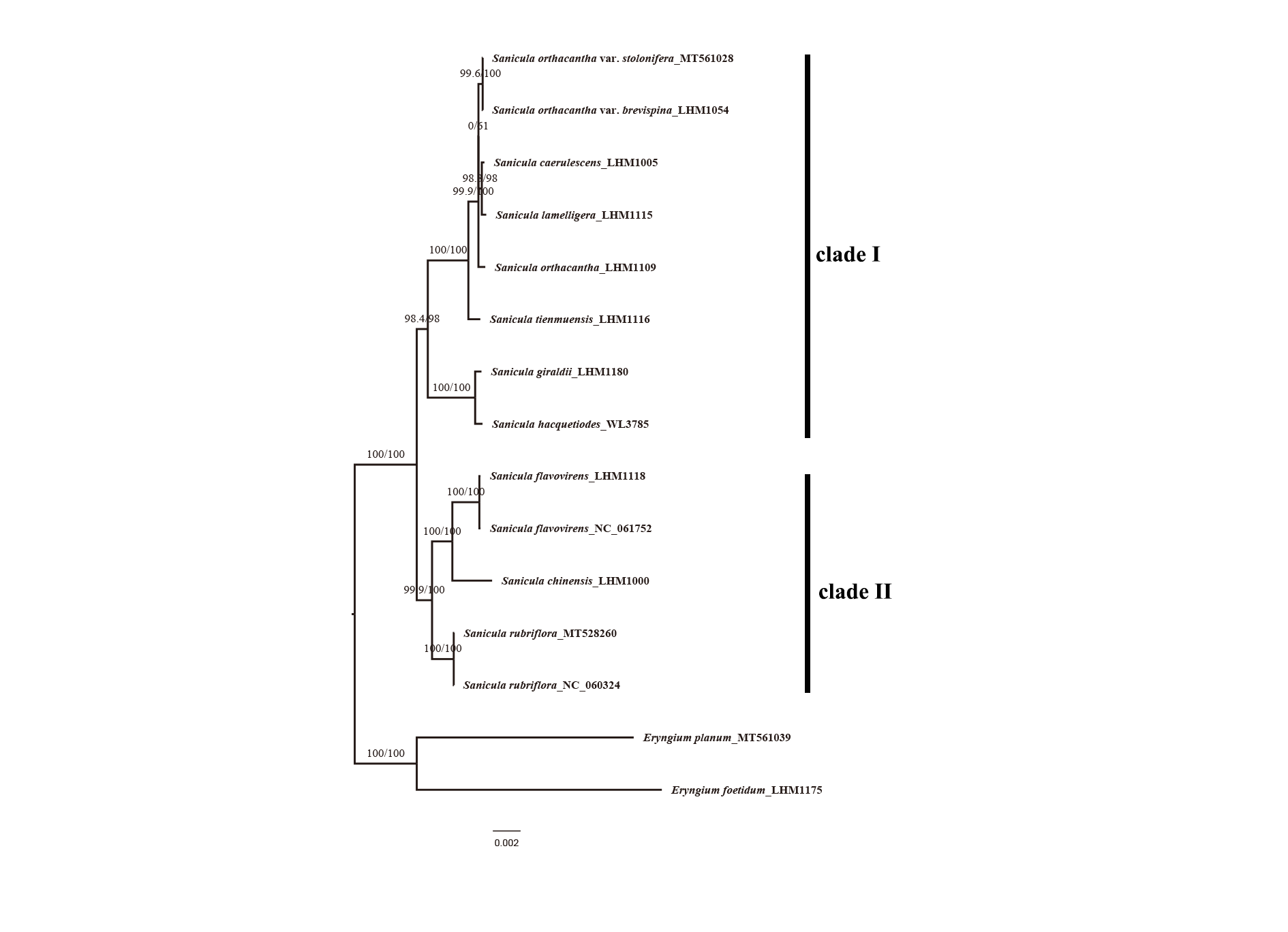

Supplement: Supplementary file 11 — Additional file 11: Fig. S7. Phylogenetic relationships of 13 Sanicula samples and two Eryngium species inferred from maximum likelihood (ML) analysis. A. trnV-rps12. B. ycf3-trnS. C. ycf4-cemA. D. ycf1. [file 12870_2023_4447_MOESM11_ESM.tif]
