# Supplementary material for: Complete chloroplast of four Sanicula taxa (Apiaceae) endemic to China: lights into genome structure, comparative analysis, and phylogenetic relationships
Source: BMC Plant Biol. 2023 Sep 21;23:444. doi: 10.1186/s12870-023-04447-w (PMC10512634; doi:10.1186/s12870-023-04447-w)
Supplement: Supplementary file 12 — Additional file 12: Table S5. Phylogenetic relationships based on the concatenation of 20 highly divergent regions (atpH-atpI, ndhC-trnM, petB-petD, petD-rpoA, petN-psbM, psaJ-rpl33, rbcL-accD, rpoB-trnC, rps16-trnQ, trnE-psbD, trnF-ndhJ, trnH-psbA, trnN-ndhF, trnS-psbZ, trnS-trnR,trnT-trnF, trnV-rps12, ycf3-trnS, ycf4-cemA, and ycf1) in 13 Sanicula samples and two Eryngium species inferred from maximum likelihood (ML) analysis. [file 12870_2023_4447_MOESM12_ESM.docx]

**Table S5**. Collecting information, voucher specimen and identification for the nine taxa of *Sanicula* L. and one species of *Eryngium* L. in the study.

| **Genus** | **Species** | **Locality** | **Voucher and Identifier** | **Longitude/Latitude** |
| --- | --- | --- | --- | --- |
| *Eryngium* L. | *E. foetidum* | Kunming, Yunnan | Huimin Li 1175 (NAS); Huimin Li | 102° 42′ 34″/25° 2′ 47″ |
| *Sanicula* L. | *S. caerulescens* | Beibei, Chongqing | H.M. Li & W. Zhou 1005 (NAS); Huimin Li | 106°23'18"/29°50'22" |
|  | *S. hacquetiodes* | Dêqên County, Dêqên, Yunnan | Long Wang, Huimin Li & Tian Li 3785 (NAS); Huimin Li | 98° 45' 37"/28° 4' 23" |
|  | *S. orthacantha* var. *brevispina* | Emeishan, Sichuan | Huimin Li & Wei Zhou 1054 (NAS); Huimin Li | 103°19′57″/29°31′11″ |
|  | *S. tienmuensis* | Lin'an County, Hangzhou, Zhejiang | Huimin Li & Lei Zhao 1116 (NAS); Huimin Li | 119°25′/30°20′ |
|  | *S. chinensis* | Nanjing, Jiangsu | Huimin Li & Min Chen 1000 (NAS); Huimin Li | 118° 52′ 48″/32° 3′ 18″ |
|  | *S. flavovirens* | Pan'an, Jinhua, Zhejiang | Huimin Li & Lei Zhao 1118 (NAS); Huimin Li | 120°32'8"/28°58'51" |
|  | *S. giraldii* | Zhouzhi, Xi'an, Shaanxi | Huimin Li & Chunfeng Song 1180 (NAS); Huimin Li | 113°12'40"/34°0'5" |
|  | *S. lamelligera* | Lin'an County, Hangzhou, Zhejiang | Huimin Li & Wei Zhou 1115 (NAS); Huimin Li | 30°19'59.49"/119°27'0.91" |
|  | *S. orthacantha* | Jiujiang, Jiangxi | Huimin Li, Yongshen Zhang & Ying Xu 1109 (NAS); Huimin Li | 115°52′/29°26′ |
